# Supplementary material for: Risk preference as an outcome of evolutionarily adaptive learning mechanisms: An evolutionary simulation under diverse risky environments
Source: PLoS One. 2024 Aug 1;19(8):e0307991. doi: 10.1371/journal.pone.0307991 (PMC11293680; doi:10.1371/journal.pone.0307991)
Supplement: S1 Text — (PDF) [file pone.0307991.s001.pdf]

S1 Text

Detail of the single-task simulation.

Here, we report the detail of the setting of three parameters and task structures. The parameters α_p, α_n, and β were assumed to be genetically inherited. The initial value of the parameters were sampled from a uniform distribution (Uniform[0, 1] for α_p and α_n; Uniform[0, 0.5] for β) and assigned to each agent. Although the maximum β is usually unlimited, we set it to 0.5 because any β larger than that leads to almost the same choice probability in our simulation (i.e., suppose that the difference between two values, $V_t(1)$– $V_t(2)$, is 10. When $\beta = 0.5$, the choice probability is 0.993 and when $\beta = 0.55$, it is 0.996).

In a task, an agent chooses one of two options and obtain payoffs randomly from a normal distribution $N(\mu_1, \sigma_1)$ or $N(\mu_2, \sigma_2)$, whose mean (μ) and standard deviation (σ) are fixed for each option and across trials. We denote a task by $N(\mu_1, \sigma_1)$ vs $N(\mu_2, \sigma_2)$, where the former is risky option and the latter is safe option, and the difference between the means of the two options ($\mu_1 - \mu_2$) by D. We fixed the standard deviation of safe option (σ_2) to 5 and varied the mean (μ_2) from 30, 20, 10, 0, –10, –20, to –30. For each of the seven safe options, we prepared 20 different risky options by combining the different means (μ_1) and standard deviations (σ_1). The μ_1 was set as such that D was +20, +10 (risk-seeking task), –10, and –20 (risk-aversion task), while σ_1 was 30, 25, 20, 15, and 10. In total, 140 tasks (all combinations of seven safe options and 20 risky options) were generated.
