# Supplementary material for: Risk preference as an outcome of evolutionarily adaptive learning mechanisms: An evolutionary simulation under diverse risky environments
Source: PLoS One. 2024 Aug 1;19(8):e0307991. doi: 10.1371/journal.pone.0307991 (PMC11293680; doi:10.1371/journal.pone.0307991)
Supplement: S2 Text — (PDF) [file pone.0307991.s002.pdf]

S2 Text

<u>Detail of multiple-task simulation</u>

We ran 100 multiple-task simulations in which four tasks were randomly determined in a specific protocol for each of the 5 conditions (the number of risk-seeking tasks that the agents experience in their life). Here we delineate the method to generate the tasks.

We generated a task group consisting of 100 tasks for each condition, half of which were risk-seeking tasks and the rest were risk-averse tasks. The risk-seeking tasks are generated as follows: first, μ_1 was randomly sampled from $N(0, 20)$, and the σ_1 was randomly determined from Uniform[5, 30]. Next, μ_2 was sampled from $N(0, 20)$ and σ_2 was fixed to 5. This procedure was repeated until the effect size of the task was more than 0 ($\mu_1 > \mu_2$) and less than 5. The effect size of the task reflects the discriminability of the two options. We set the upper bound of the effect size to exclude tasks with extremely high discriminability, which makes it easy for agents to learn the more rewarding option. The risk-aversion tasks were created by inverting the two options of the risk-seeking tasks by the vertical axis (x = 0). This method creates a symmetric distribution of the effect size near zero (S6 Fig.a). Moreover, we confirmed that in the task group generated by this procedure, the number of tasks whose distributions were positively and negatively biased was almost the same (S6 Fig.b). For each simulation, four tasks were randomly sampled from the task set depending on the simulation conditions. For example, in the condition where two risk-seeking tasks were performed, two tasks were randomly sampled from the 50 risk-seeking tasks and the rest were sampled from the 50 risk-aversion tasks.

We used Cohen's *d* for the effect size, which indicates the degree to which two normal distributions overlapped. If the difference between the expected values was large or the SD of the distribution was small, the absolute value of the effect size was large. We calculated Cohen's *d* by setting the sample size to be identical for the two groups,

$$d = \frac{\mu_1 - \mu_2}{\sigma_p} \quad (S1)$$

$$\sigma_p = \sqrt{\frac{\sigma_1^2 + \sigma_2^2}{2}} \quad (S2)$$

where μ_1 and μ_2 are the expected values for the risky and safe option, respectively, and σ_p is the pooled SD calculated from the SD of the risky (σ_1) and safe option (σ_2). Thus, a positive *d* indicates a risk-seeking task and a negative *d* indicates a risk-aversion task.

The negative area rate was calculated as the averaged cumulative density of the two normal distributions $[P_1(X \leq 0) + P_2(X \leq 0)]/2$ where P_1 and P_2 represents the probability functions of the risky and safe options, respectively. The negative area ratio represents the probability that a negative outcome was sampled during the task.
