# Supplementary material for: Risk preference as an outcome of evolutionarily adaptive learning mechanisms: An evolutionary simulation under diverse risky environments
Source: PLoS One. 2024 Aug 1;19(8):e0307991. doi: 10.1371/journal.pone.0307991 (PMC11293680; doi:10.1371/journal.pone.0307991)
Supplement: S3 Text — (PDF) [file pone.0307991.s003.pdf]

S3 Text

Reinforcement learning involving the perseverance parameter

To test whether the evolved relationship $\alpha_p > \alpha_n$ disappears if considering a perseverance parameter, we simulated the reinforcement learning model involving two learning rates and the perseverance parameter (the Hybrid model) [1]. We omit the counterfactual outcomes included in Sugawara and Katahira's (2021) model.

In the Hybrid model, the value update rule was the same as that in the asymmetric reinforcement learning model. The probability of choosing option a in trial t is calculated as follows:

$$p_t(a) = \frac{1}{1 + \exp\left(-\beta\left(V_t(a) - V_t(\bar{a})\right) - \varphi\left(C_t(a) - C_t(\bar{a})\right)\right)} \tag{S3}$$

Here, $\bar{a}$ denotes the other option while C_a and $C_{\bar{a}}$ represent the choice traces. The perseverance parameter (φ) regulates the degree of repeating or avoiding the previous choice. Hereafter, we consider $\varphi > 0$ for considering only the effect of repeating the same choice. The choice trace is updated according to the selected option.

$$C_{t+1}(\text{chosen}) = C_t(\text{chosen}) + \tau\left(1 - C_t(\text{chosen})\right) \tag{S4}$$
$$C_{t+1}(\text{unchosen}) = C_t(\text{unchosen}) + \tau\left(0 - C_t(\text{unchosen})\right)$$

The decay rate parameter (τ) regulates the number of previous trials influencing the current choice probability. For simplification, we fixed $\tau = 1$, considering only the effect of the

immediately preceding choice. In summary, when the choice in trial $t-1$ is 1, the probability of choosing option 1 in trial t is calculated as follows:

$$p_t(a=1) = \frac{1}{1+\exp\left(-\beta\left(V_t(1)-V_t(2)\right)-\varphi\right)} \tag{S5}$$

When the choice in trial $t-1$ is 2, the probability is calculated as follows:

$$p_t(a=1) = \frac{1}{1+\exp\left(-\beta\left(V_t(1)-V_t(2)\right)+\varphi\right)} \tag{S6}$$

In the multiple-task simulations, we simulated the Hybrid model by setting α_p, α_n, β, and φ as genes. The perseverance parameter (φ) in the first generation was randomly set from Uniform[0, 10]. In the mutation, Gaussian noise was added from $N(0, 0.1)$. The initial settings of the other parameters and the evolutionary algorithm were the same as those in the asymmetric reinforcement learning model (described in the main text). We used the same task group for the multiple-task simulations as in the asymmetric reinforcement learning model (see S6 for detailed features of the task group in each simulation condition).

Reference

1. Sugawara M, Katahira K. Dissociation between asymmetric value updating and perseverance in human reinforcement learning. Sci Rep. 2021;11: 3574. doi:10.1038/s41598-020-80593-7
