# Supplementary material for: Risk preference as an outcome of evolutionarily adaptive learning mechanisms: An evolutionary simulation under diverse risky environments
Source: PLoS One. 2024 Aug 1;19(8):e0307991. doi: 10.1371/journal.pone.0307991 (PMC11293680; doi:10.1371/journal.pone.0307991)
Supplement: S4 Text — (PDF) [file pone.0307991.s004.pdf]

S4 Text

Reinforcement learning using a single learning rate

We conducted multiple-task simulations of the reinforcement learning model with a single learning rate (hereafter referred to as the single-learning rate model). The value of learning rate (α) was randomly set from Uniform[0, 1] and Gaussian noise was added from $N(0, 0.01)$ in the mutation. The evolutionary algorithm was the same as that of the asymmetric reinforcement learning model (see the main text). We used the same task group for the multiple-task simulations as in the asymmetric reinforcement learning model (see S6 for detailed features of the task group in each simulation condition).
