# Supplementary material for: Risk preference as an outcome of evolutionarily adaptive learning mechanisms: An evolutionary simulation under diverse risky environments
Source: PLoS One. 2024 Aug 1;19(8):e0307991. doi: 10.1371/journal.pone.0307991 (PMC11293680; doi:10.1371/journal.pone.0307991)
Supplement: S1 Fig — The horizontal axis represents the location of distributions depicted by μ (risky option vs safe option). Each panel corresponds to the different risks of the risky option. The white and black circle indicates the mean rate of risk aversion in the first and last generation, respectively (the averaged result of 10 simulations with the same task parameter setting). The vertical bar is ±1 standard deviation (mean of 10 simulations’ SD). As reported in the main text (tasks with D = ±20), agents evolved to choose the more rewarding option in the last generation. (PDF) [file pone.0307991.s005.pdf]

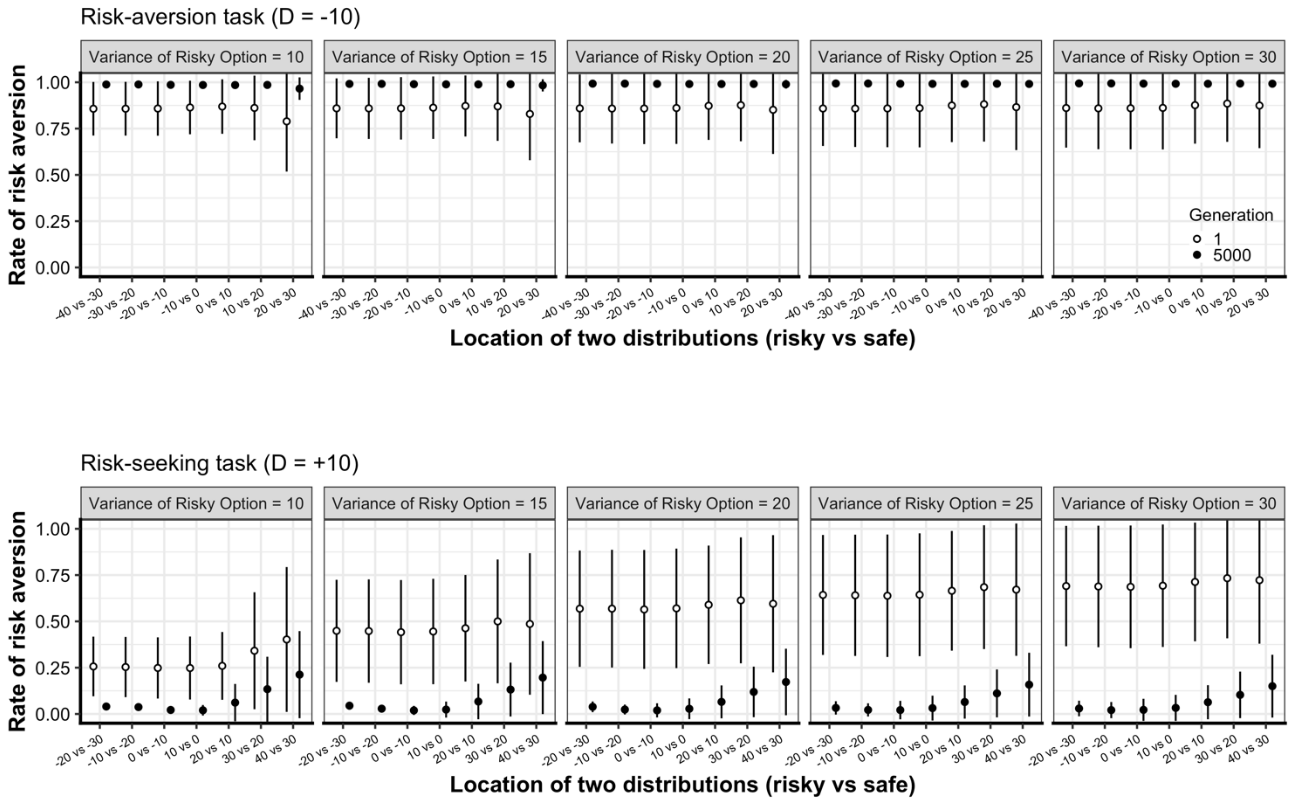

S1 Fig. Risk aversion rate in 70 single tasks with D = ±10 in the first and last generation. The horizontal axis represents the location of distributions depicted by μ (risky option vs safe option). Each panel corresponds to the different standard deviation of the risky option (σ_1 of normal distribution). The white and black circle indicates the mean rate of risk aversion in the first and last generation, respectively (the averaged result of 10 simulations with the same task parameter setting). The vertical bar is ±1 standard deviation (mean of 10 simulations' SD). As reported in the main text (tasks with D = ±20), agents evolved to choose the more rewarding option in the last generation.
