# Supplementary material for: Risk preference as an outcome of evolutionarily adaptive learning mechanisms: An evolutionary simulation under diverse risky environments
Source: PLoS One. 2024 Aug 1;19(8):e0307991. doi: 10.1371/journal.pone.0307991 (PMC11293680; doi:10.1371/journal.pone.0307991)
Supplement: S2 Fig — The solid line and colored area (αn is blue and αP is orange) are the mean value and SD, respectively, which were averaged over 10 simulations conducted with the same parameter setting. The column indicates the risk of the risky option (σ1). The row indicates the normal distribution of the safe option. Each panel corresponds to a single task. The task distribution is depicted by “risky option vs safe option” inside a panel. In the main text, we only report the mean value of parameters in the last generation. This figure shows that the mean value of learning rates becomes almost steady within 5,000 generations in almost all of tasks. (PDF) [file pone.0307991.s006.pdf]

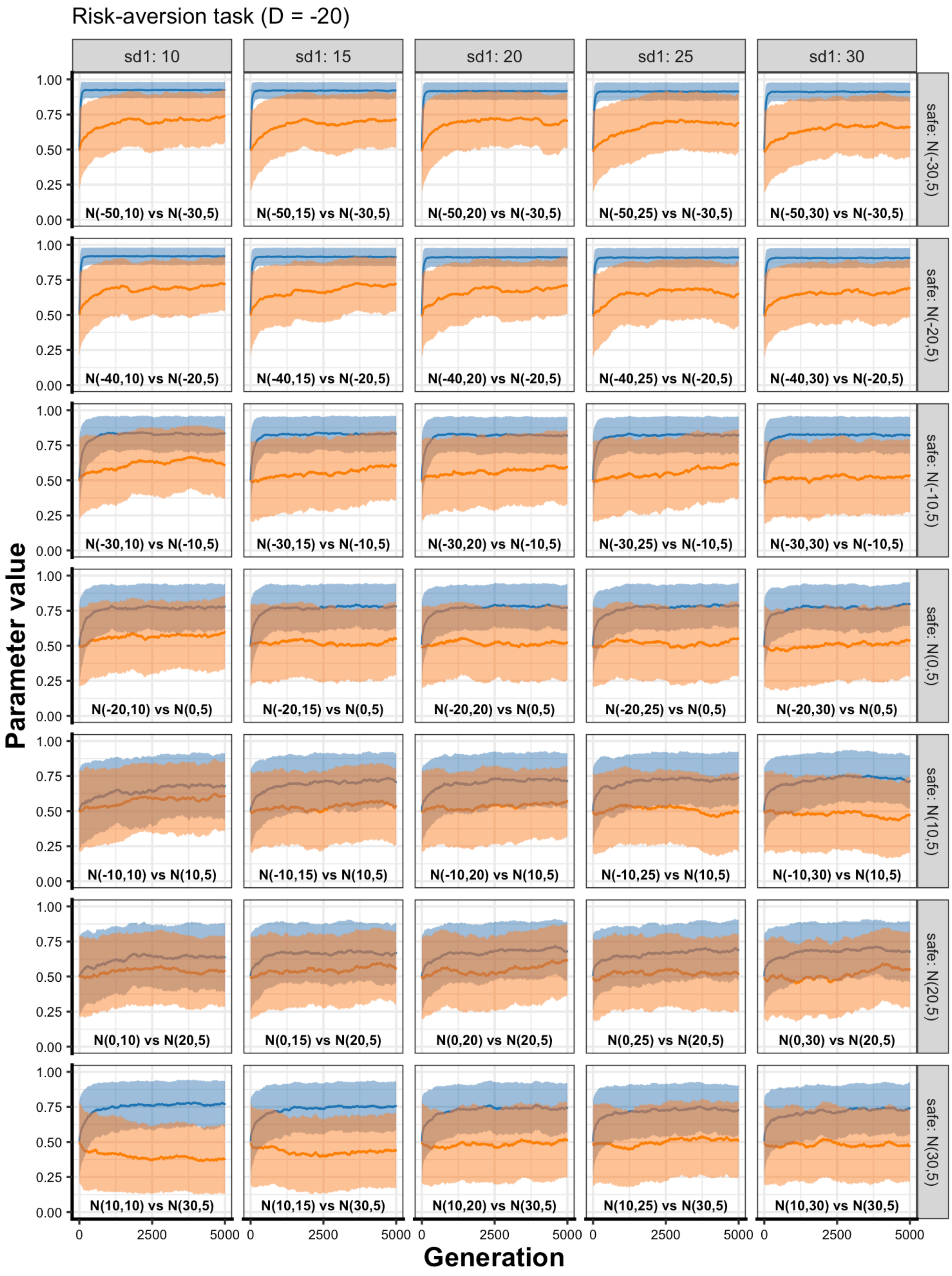

Risk-aversion task (D = -20)
sd1: 10
sd1: 15
sd1: 20
sd1: 25
sd1: 30
safe: N(-30,5)
safe: N(-20,5)
safe: N(-10,5)
safe: N(0,5)
safe: N(10,5)
safe: N(20,5)
safe: N(30,5)
N(-50,10) vs N(-30,5)
N(-50,15) vs N(-30,5)
N(-50,20) vs N(-30,5)
N(-50,25) vs N(-30,5)
N(-50,30) vs N(-30,5)
N(-40,10) vs N(-20,5)
N(-40,15) vs N(-20,5)
N(-40,20) vs N(-20,5)
N(-40,25) vs N(-20,5)
N(-40,30) vs N(-20,5)
N(-30,10) vs N(-10,5)
N(-30,15) vs N(-10,5)
N(-30,20) vs N(-10,5)
N(-30,25) vs N(-10,5)
N(-30,30) vs N(-10,5)
N(-20,10) vs N(0,5)
N(-20,15) vs N(0,5)
N(-20,20) vs N(0,5)
N(-20,25) vs N(0,5)
N(-20,30) vs N(0,5)
N(-10,10) vs N(10,5)
N(-10,15) vs N(10,5)
N(-10,20) vs N(10,5)
N(-10,25) vs N(10,5)
N(-10,30) vs N(10,5)
N(0,10) vs N(20,5)
N(0,15) vs N(20,5)
N(0,20) vs N(20,5)
N(0,25) vs N(20,5)
N(0,30) vs N(20,5)
N(10,10) vs N(30,5)
N(10,15) vs N(30,5)
N(10,20) vs N(30,5)
N(10,25) vs N(30,5)
N(10,30) vs N(30,5)
Parameter value
0.00
0.25
0.50
0.75
1.00
Generation
0
2500
5000

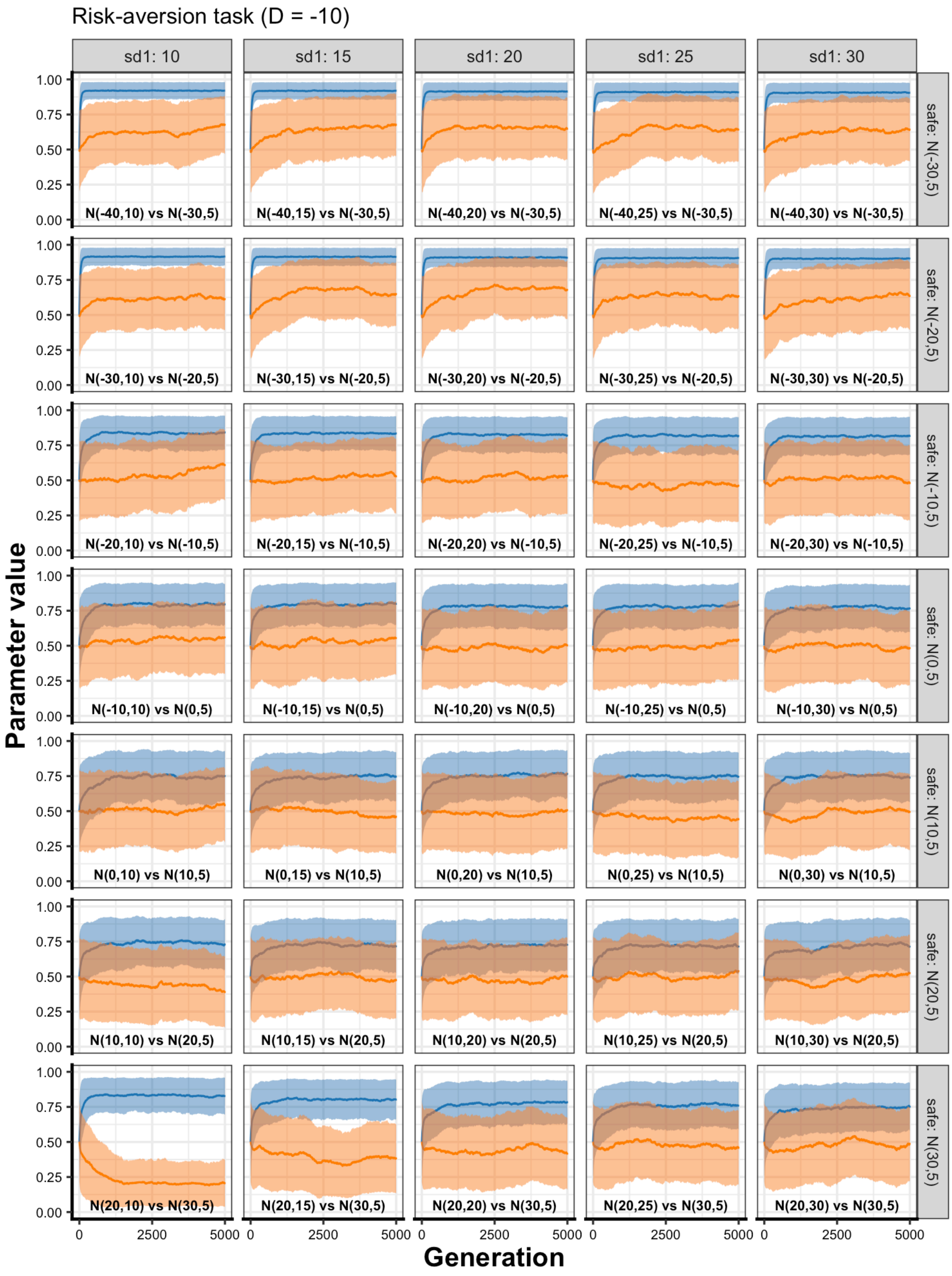

Risk-aversion task (D = -10)
sd1: 10
sd1: 15
sd1: 20
sd1: 25
sd1: 30
safe: N(-30,5)
safe: N(-20,5)
safe: N(-10,5)
safe: N(0,5)
safe: N(10,5)
safe: N(20,5)
safe: N(30,5)
N(-40,10) vs N(-30,5)
N(-40,15) vs N(-30,5)
N(-40,20) vs N(-30,5)
N(-40,25) vs N(-30,5)
N(-40,30) vs N(-30,5)
N(-30,10) vs N(-20,5)
N(-30,15) vs N(-20,5)
N(-30,20) vs N(-20,5)
N(-30,25) vs N(-20,5)
N(-30,30) vs N(-20,5)
N(-20,10) vs N(-10,5)
N(-20,15) vs N(-10,5)
N(-20,20) vs N(-10,5)
N(-20,25) vs N(-10,5)
N(-20,30) vs N(-10,5)
N(-10,10) vs N(0,5)
N(-10,15) vs N(0,5)
N(-10,20) vs N(0,5)
N(-10,25) vs N(0,5)
N(-10,30) vs N(0,5)
N(0,10) vs N(10,5)
N(0,15) vs N(10,5)
N(0,20) vs N(10,5)
N(0,25) vs N(10,5)
N(0,30) vs N(10,5)
N(10,10) vs N(20,5)
N(10,15) vs N(20,5)
N(10,20) vs N(20,5)
N(10,25) vs N(20,5)
N(10,30) vs N(20,5)
N(20,10) vs N(30,5)
N(20,15) vs N(30,5)
N(20,20) vs N(30,5)
N(20,25) vs N(30,5)
N(20,30) vs N(30,5)
Parameter value
0.00
0.25
0.50
0.75
1.00
Generation
0
2500
5000

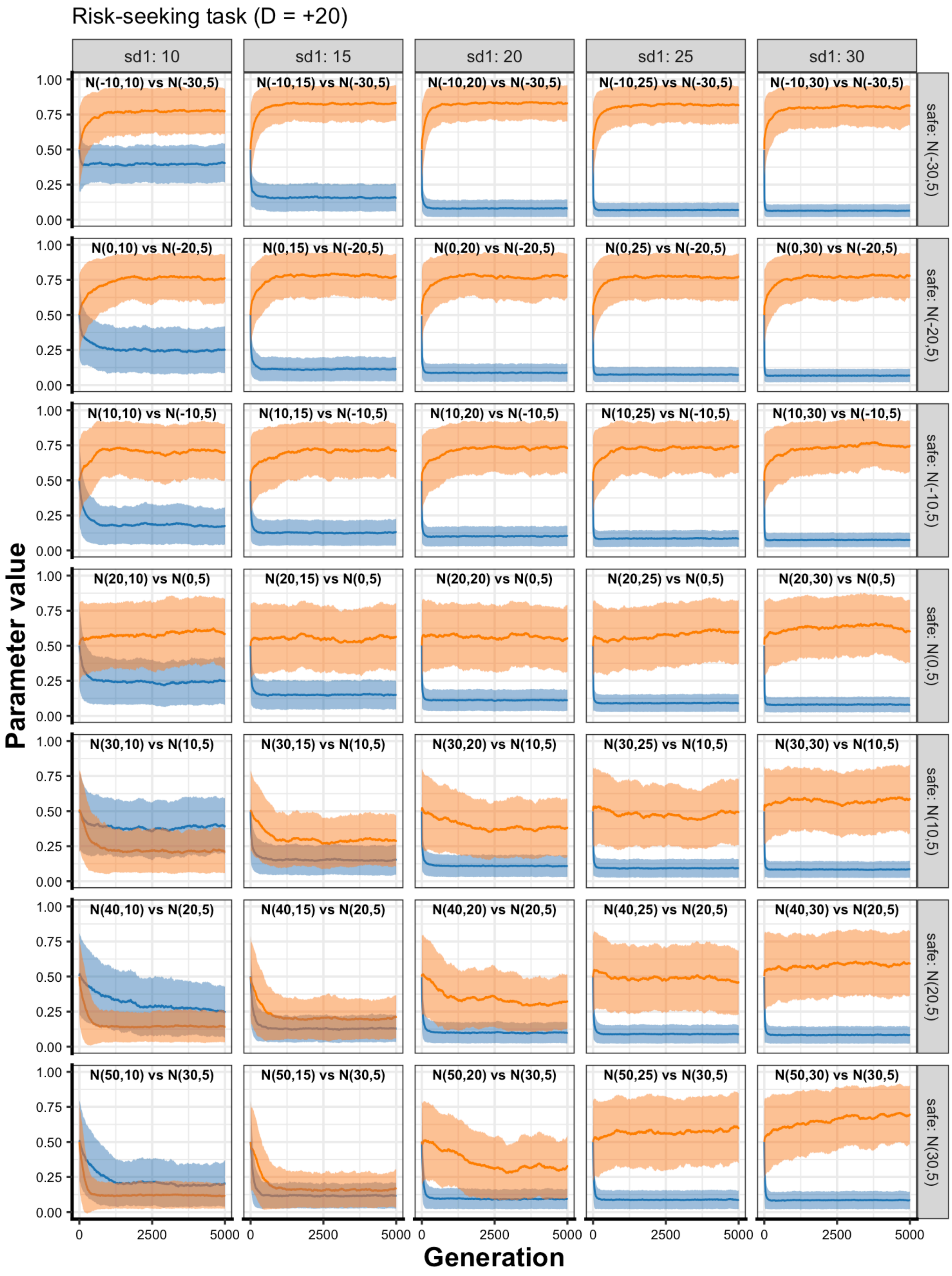
Risk-seeking task (D = +20)
sd1: 10
sd1: 15
sd1: 20
sd1: 25
sd1: 30
safe: N(-30,5)
safe: N(-20,5)
safe: N(-10,5)
safe: N(0,5)
safe: N(10,5)
safe: N(20,5)
safe: N(30,5)
N(-10,10) vs N(-30,5)
N(-10,15) vs N(-30,5)
N(-10,20) vs N(-30,5)
N(-10,25) vs N(-30,5)
N(-10,30) vs N(-30,5)
N(0,10) vs N(-20,5)
N(0,15) vs N(-20,5)
N(0,20) vs N(-20,5)
N(0,25) vs N(-20,5)
N(0,30) vs N(-20,5)
N(10,10) vs N(-10,5)
N(10,15) vs N(-10,5)
N(10,20) vs N(-10,5)
N(10,25) vs N(-10,5)
N(10,30) vs N(-10,5)
N(20,10) vs N(0,5)
N(20,15) vs N(0,5)
N(20,20) vs N(0,5)
N(20,25) vs N(0,5)
N(20,30) vs N(0,5)
N(30,10) vs N(10,5)
N(30,15) vs N(10,5)
N(30,20) vs N(10,5)
N(30,25) vs N(10,5)
N(30,30) vs N(10,5)
N(40,10) vs N(20,5)
N(40,15) vs N(20,5)
N(40,20) vs N(20,5)
N(40,25) vs N(20,5)
N(40,30) vs N(20,5)
N(50,10) vs N(30,5)
N(50,15) vs N(30,5)
N(50,20) vs N(30,5)
N(50,25) vs N(30,5)
N(50,30) vs N(30,5)
Parameter value
0.00
0.25
0.50
0.75
1.00
Generation
0
2500
5000

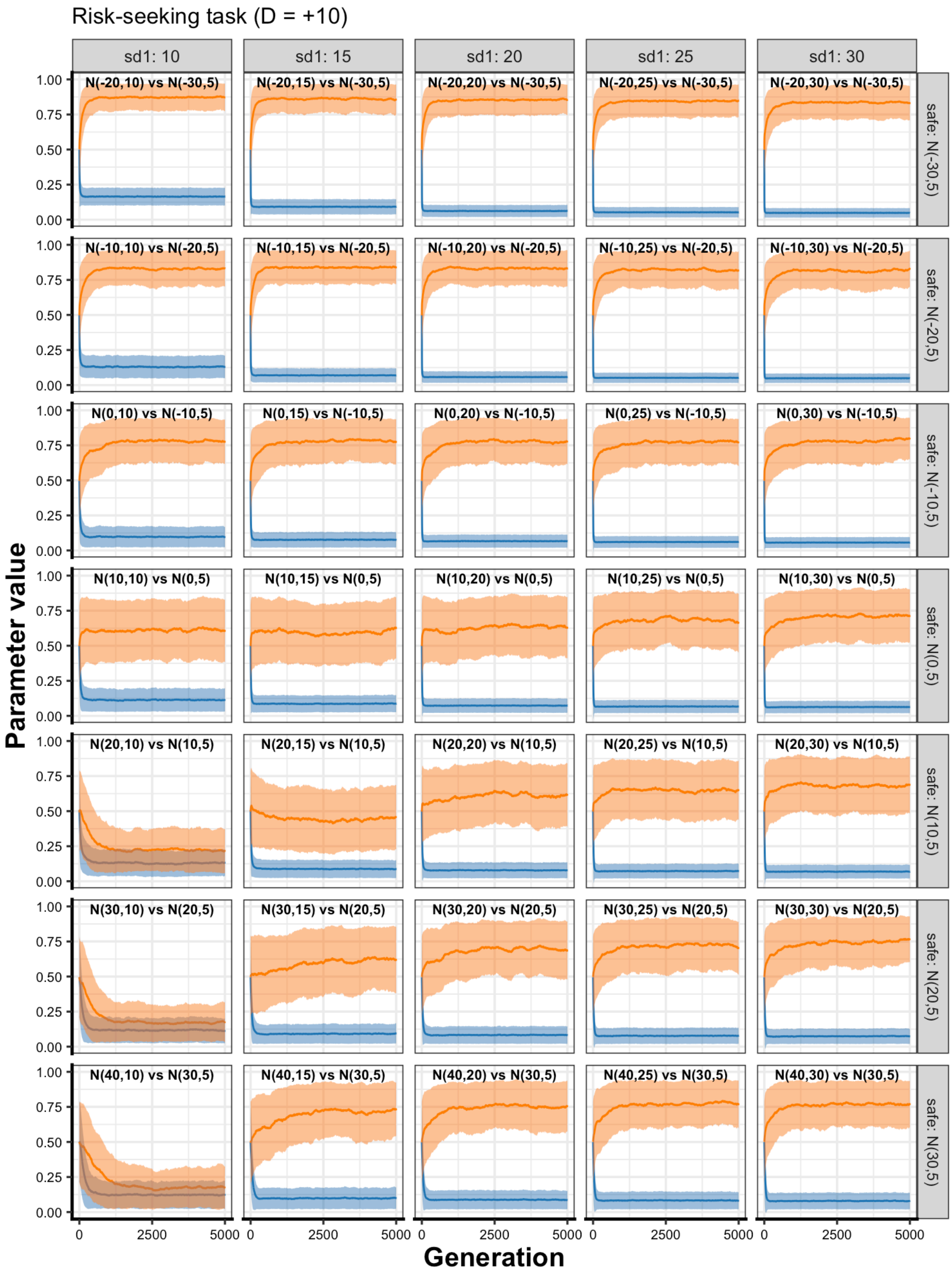

Risk-seeking task (D = +10)
sd1: 10
sd1: 15
sd1: 20
sd1: 25
sd1: 30
N(-20,10) vs N(-30,5)
N(-20,15) vs N(-30,5)
N(-20,20) vs N(-30,5)
N(-20,25) vs N(-30,5)
N(-20,30) vs N(-30,5)
safe: N(-30,5)
N(-10,10) vs N(-20,5)
N(-10,15) vs N(-20,5)
N(-10,20) vs N(-20,5)
N(-10,25) vs N(-20,5)
N(-10,30) vs N(-20,5)
safe: N(-20,5)
N(0,10) vs N(-10,5)
N(0,15) vs N(-10,5)
N(0,20) vs N(-10,5)
N(0,25) vs N(-10,5)
N(0,30) vs N(-10,5)
safe: N(-10,5)
N(10,10) vs N(0,5)
N(10,15) vs N(0,5)
N(10,20) vs N(0,5)
N(10,25) vs N(0,5)
N(10,30) vs N(0,5)
safe: N(0,5)
N(20,10) vs N(10,5)
N(20,15) vs N(10,5)
N(20,20) vs N(10,5)
N(20,25) vs N(10,5)
N(20,30) vs N(10,5)
safe: N(10,5)
N(30,10) vs N(20,5)
N(30,15) vs N(20,5)
N(30,20) vs N(20,5)
N(30,25) vs N(20,5)
N(30,30) vs N(20,5)
safe: N(20,5)
N(40,10) vs N(30,5)
N(40,15) vs N(30,5)
N(40,20) vs N(30,5)
N(40,25) vs N(30,5)
N(40,30) vs N(30,5)
safe: N(30,5)
Parameter value
1.00
0.75
0.50
0.25
0.00
Generation
0
2500
5000

S2 Fig. Comprehensive display of evolutionary dynamics of α_n and α_p in 140 single-task simulations. The solid line and colored area (α_n is blue and α_p is orange) are the mean value and SD, respectively, which were averaged over 10 simulations conducted with the same parameter setting. The column indicates the risk of the risky option (σ_1). The row indicates the normal distribution of the safe option. Each panel corresponds to a single task. The task distribution is depicted by "risky option vs safe option" inside a panel. In the main text, we only report the mean value of parameters in the last generation. This figure shows that the mean value of learning rates becomes almost steady within 5,000 generations in almost all of tasks.
