# Supplementary material for: Risk preference as an outcome of evolutionarily adaptive learning mechanisms: An evolutionary simulation under diverse risky environments
Source: PLoS One. 2024 Aug 1;19(8):e0307991. doi: 10.1371/journal.pone.0307991 (PMC11293680; doi:10.1371/journal.pone.0307991)
Supplement: S3 Fig — The horizontal axis represents the location of distributions. Each panel corresponds to the different risks of risky option (σ1). The white circle indicates the mean value in the last generation (the averaged result of 10 simulations with the same task parameter setting). The vertical bar is ±1 SD (mean of 10 simulations’ SD). As reported in the main text (tasks with D = ±20), the parameter relationship αn > αp was consistently observed for the risk-aversion tasks, and the reversed relationship αp > αn was observed for the risk-seeking tasks. (PDF) [file pone.0307991.s007.pdf]

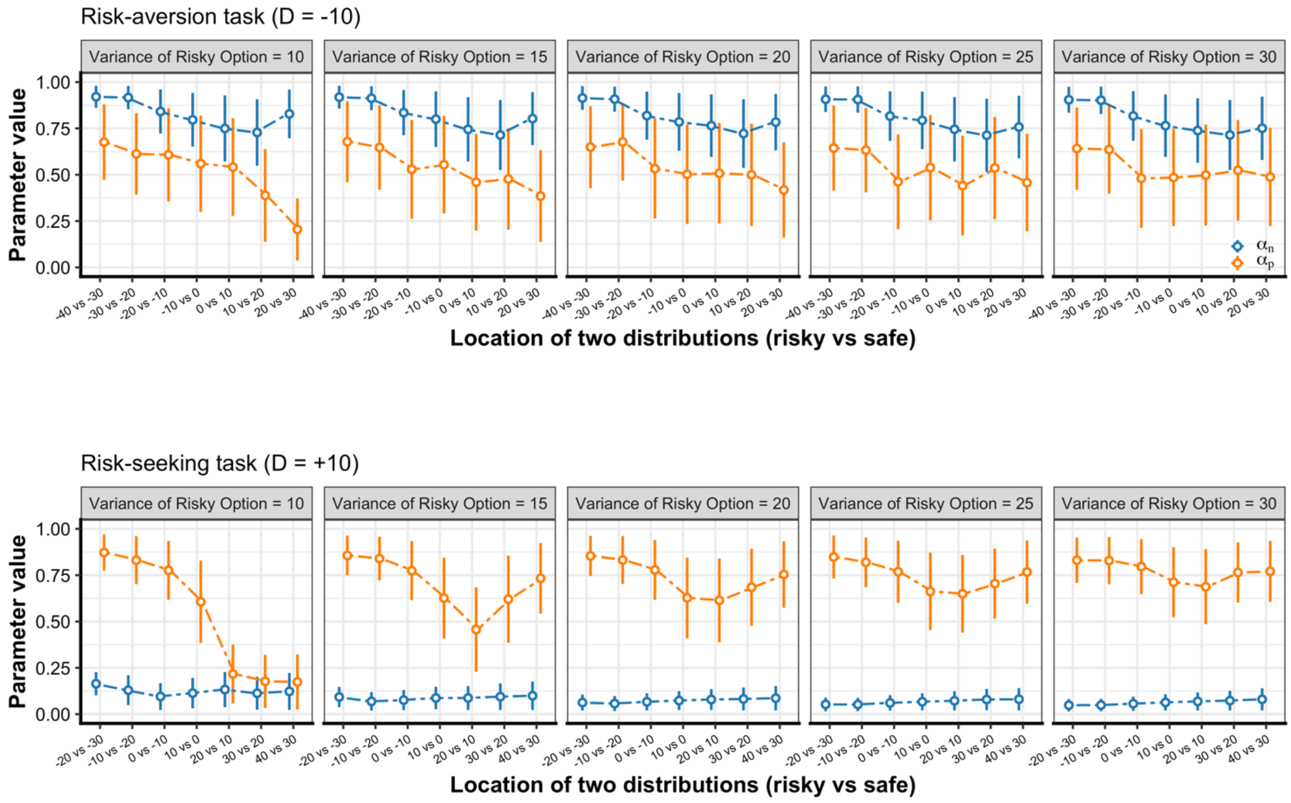


**Fig S3. Evolutionary result of $\alpha_n$ and $\alpha_p$ in 70 single tasks with $D$ = ±10.** The horizontal axis represents the location of distributions. Each panel corresponds to the different standard deviation of risky option ($\sigma_1$). The white circle indicates the mean value in the last generation (the averaged result of 10 simulations with the same task parameter setting). The vertical bar is ±1 SD (mean of 10 simulations' SD). As reported in the main text (tasks with $D$ = ±20), the parameter relationship $\alpha_n > \alpha_p$ was consistently observed for the risk-aversion tasks, and the reversed relationship $\alpha_p > \alpha_n$ was observed for the risk-seeking tasks.
