# Supplementary material for: Risk preference as an outcome of evolutionarily adaptive learning mechanisms: An evolutionary simulation under diverse risky environments
Source: PLoS One. 2024 Aug 1;19(8):e0307991. doi: 10.1371/journal.pone.0307991 (PMC11293680; doi:10.1371/journal.pone.0307991)
Supplement: S4 Fig — The solid line and colored area are the mean value and SD, respectively, which were averaged over 10 simulations conducted with the same parameter setting. The column indicates the risk of the risky option (σ1). The row indicates the normal distribution of the safe option. Each panel corresponds to a single task. The task distribution is depicted by “risky option vs safe option” inside a panel. (PDF) [file pone.0307991.s008.pdf]

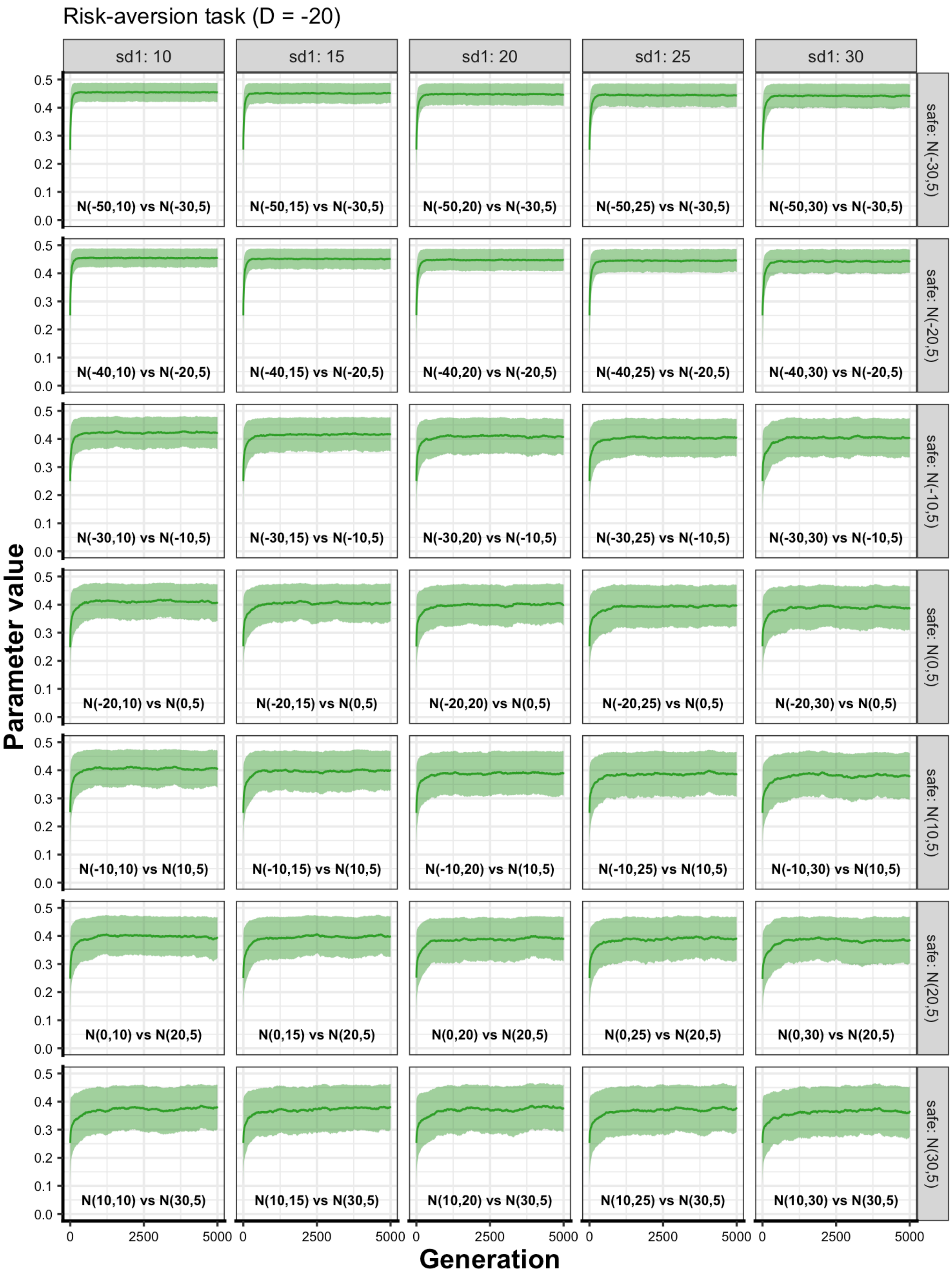
Risk-aversion task (D = -20)
sd1: 10
sd1: 15
sd1: 20
sd1: 25
sd1: 30
safe: N(-30,5)
safe: N(-20,5)
safe: N(-10,5)
safe: N(0,5)
safe: N(10,5)
safe: N(20,5)
safe: N(30,5)
N(-50,10) vs N(-30,5)
N(-50,15) vs N(-30,5)
N(-50,20) vs N(-30,5)
N(-50,25) vs N(-30,5)
N(-50,30) vs N(-30,5)
N(-40,10) vs N(-20,5)
N(-40,15) vs N(-20,5)
N(-40,20) vs N(-20,5)
N(-40,25) vs N(-20,5)
N(-40,30) vs N(-20,5)
N(-30,10) vs N(-10,5)
N(-30,15) vs N(-10,5)
N(-30,20) vs N(-10,5)
N(-30,25) vs N(-10,5)
N(-30,30) vs N(-10,5)
N(-20,10) vs N(0,5)
N(-20,15) vs N(0,5)
N(-20,20) vs N(0,5)
N(-20,25) vs N(0,5)
N(-20,30) vs N(0,5)
N(-10,10) vs N(10,5)
N(-10,15) vs N(10,5)
N(-10,20) vs N(10,5)
N(-10,25) vs N(10,5)
N(-10,30) vs N(10,5)
N(0,10) vs N(20,5)
N(0,15) vs N(20,5)
N(0,20) vs N(20,5)
N(0,25) vs N(20,5)
N(0,30) vs N(20,5)
N(10,10) vs N(30,5)
N(10,15) vs N(30,5)
N(10,20) vs N(30,5)
N(10,25) vs N(30,5)
N(10,30) vs N(30,5)
0.0
0.1
0.2
0.3
0.4
0.5
Parameter value
0
2500
5000
Generation

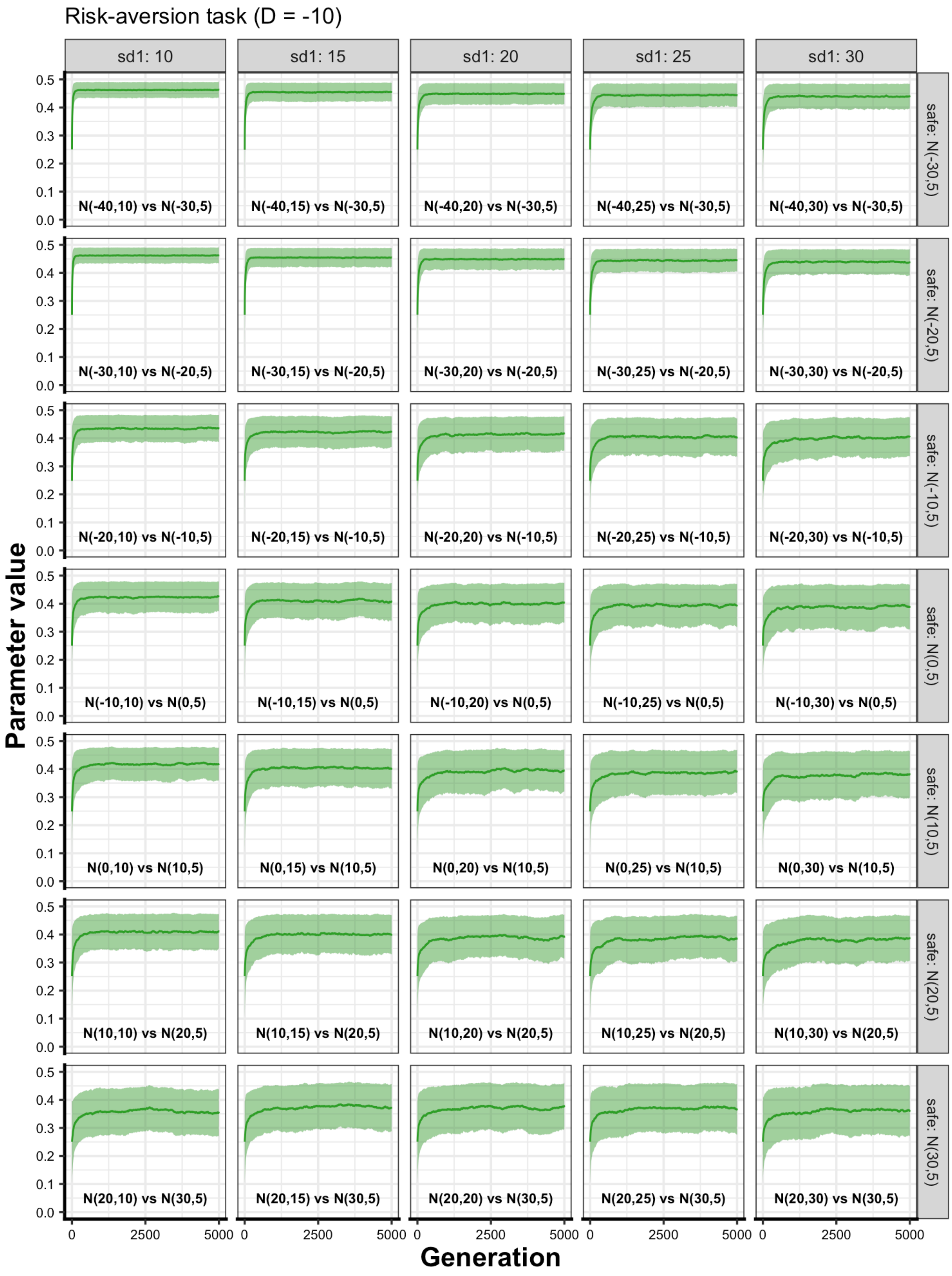

Risk-aversion task (D = -10)
sd1: 10
sd1: 15
sd1: 20
sd1: 25
sd1: 30
safe: N(-30,5)
safe: N(-20,5)
safe: N(-10,5)
safe: N(0,5)
safe: N(10,5)
safe: N(20,5)
safe: N(30,5)
N(-40,10) vs N(-30,5)
N(-40,15) vs N(-30,5)
N(-40,20) vs N(-30,5)
N(-40,25) vs N(-30,5)
N(-40,30) vs N(-30,5)
N(-30,10) vs N(-20,5)
N(-30,15) vs N(-20,5)
N(-30,20) vs N(-20,5)
N(-30,25) vs N(-20,5)
N(-30,30) vs N(-20,5)
N(-20,10) vs N(-10,5)
N(-20,15) vs N(-10,5)
N(-20,20) vs N(-10,5)
N(-20,25) vs N(-10,5)
N(-20,30) vs N(-10,5)
N(-10,10) vs N(0,5)
N(-10,15) vs N(0,5)
N(-10,20) vs N(0,5)
N(-10,25) vs N(0,5)
N(-10,30) vs N(0,5)
N(0,10) vs N(10,5)
N(0,15) vs N(10,5)
N(0,20) vs N(10,5)
N(0,25) vs N(10,5)
N(0,30) vs N(10,5)
N(10,10) vs N(20,5)
N(10,15) vs N(20,5)
N(10,20) vs N(20,5)
N(10,25) vs N(20,5)
N(10,30) vs N(20,5)
N(20,10) vs N(30,5)
N(20,15) vs N(30,5)
N(20,20) vs N(30,5)
N(20,25) vs N(30,5)
N(20,30) vs N(30,5)
Parameter value
0.0 0.1 0.2 0.3 0.4 0.5
Generation
0 2500 5000

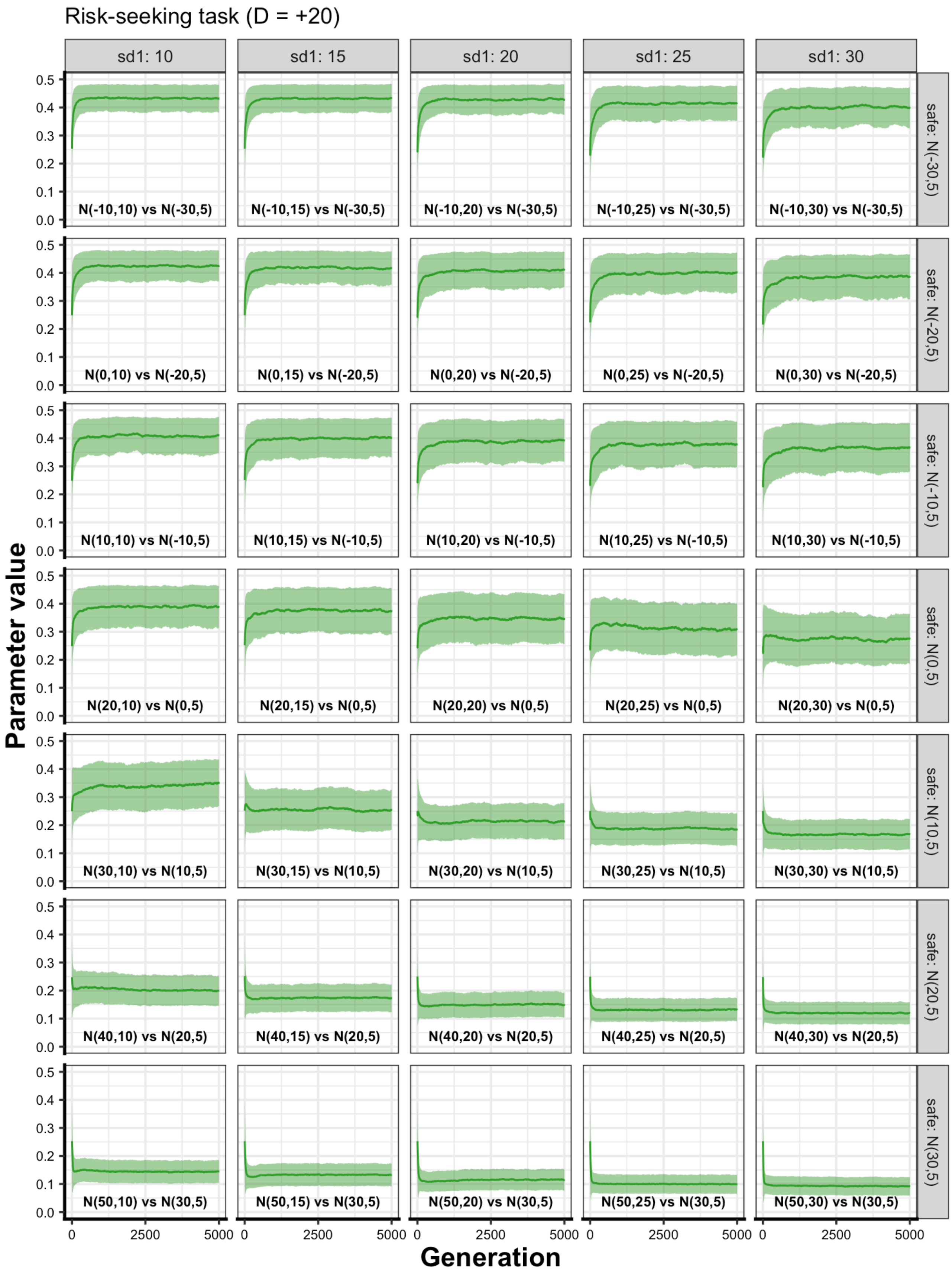
Risk-seeking task (D = +20)
sd1: 10
sd1: 15
sd1: 20
sd1: 25
sd1: 30
safe: N(-30,5)
safe: N(-20,5)
safe: N(-10,5)
safe: N(0,5)
safe: N(10,5)
safe: N(20,5)
safe: N(30,5)
N(-10,10) vs N(-30,5)
N(-10,15) vs N(-30,5)
N(-10,20) vs N(-30,5)
N(-10,25) vs N(-30,5)
N(-10,30) vs N(-30,5)
N(0,10) vs N(-20,5)
N(0,15) vs N(-20,5)
N(0,20) vs N(-20,5)
N(0,25) vs N(-20,5)
N(0,30) vs N(-20,5)
N(10,10) vs N(-10,5)
N(10,15) vs N(-10,5)
N(10,20) vs N(-10,5)
N(10,25) vs N(-10,5)
N(10,30) vs N(-10,5)
N(20,10) vs N(0,5)
N(20,15) vs N(0,5)
N(20,20) vs N(0,5)
N(20,25) vs N(0,5)
N(20,30) vs N(0,5)
N(30,10) vs N(10,5)
N(30,15) vs N(10,5)
N(30,20) vs N(10,5)
N(30,25) vs N(10,5)
N(30,30) vs N(10,5)
N(40,10) vs N(20,5)
N(40,15) vs N(20,5)
N(40,20) vs N(20,5)
N(40,25) vs N(20,5)
N(40,30) vs N(20,5)
N(50,10) vs N(30,5)
N(50,15) vs N(30,5)
N(50,20) vs N(30,5)
N(50,25) vs N(30,5)
N(50,30) vs N(30,5)
Parameter value
0.0
0.1
0.2
0.3
0.4
0.5
Generation
0
2500
5000

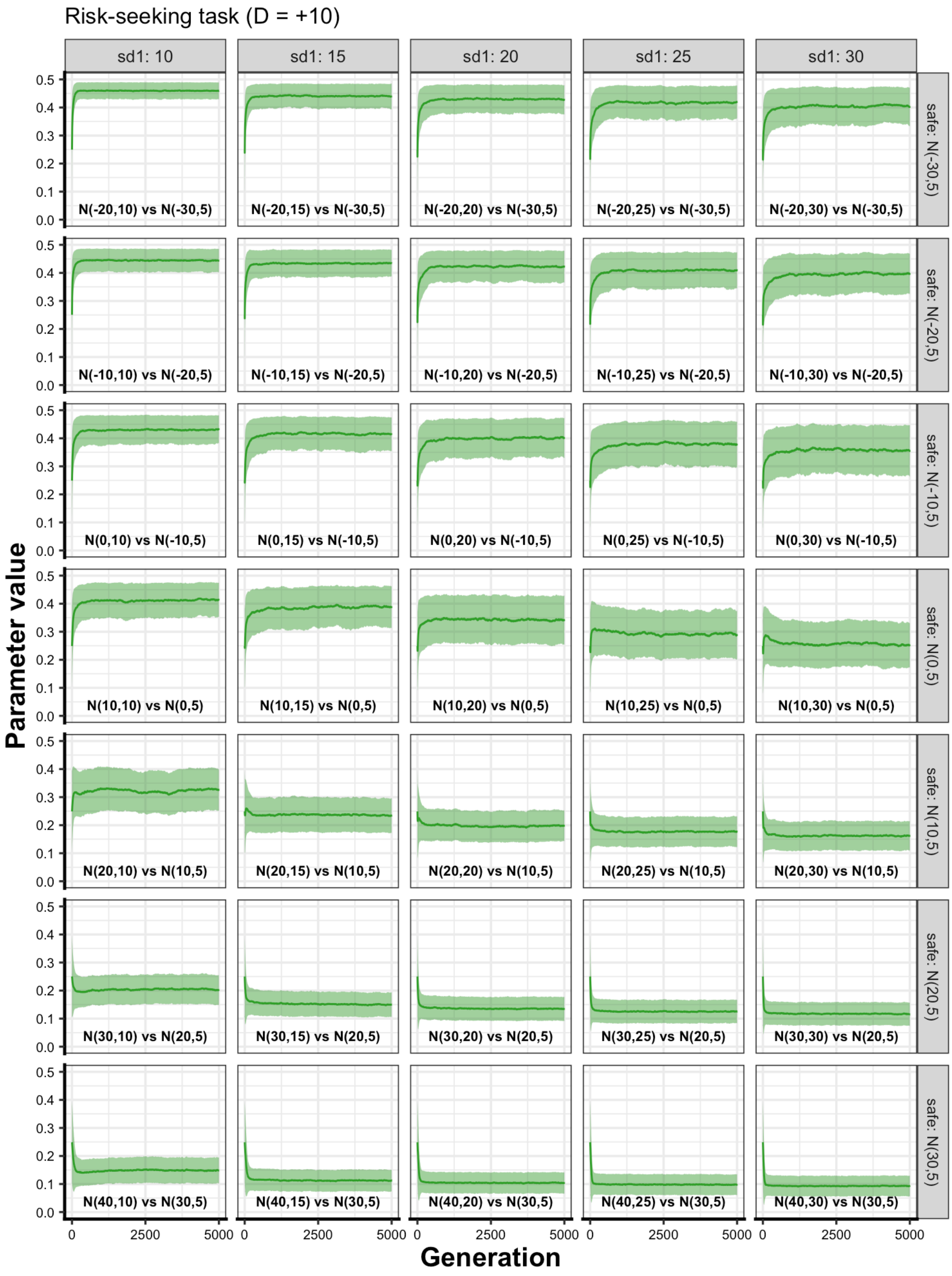
Risk-seeking task (D = +10)
sd1: 10
sd1: 15
sd1: 20
sd1: 25
sd1: 30
safe: N(-30,5)
safe: N(-20,5)
safe: N(-10,5)
safe: N(0,5)
safe: N(10,5)
safe: N(20,5)
safe: N(30,5)
N(-20,10) vs N(-30,5)
N(-20,15) vs N(-30,5)
N(-20,20) vs N(-30,5)
N(-20,25) vs N(-30,5)
N(-20,30) vs N(-30,5)
N(-10,10) vs N(-20,5)
N(-10,15) vs N(-20,5)
N(-10,20) vs N(-20,5)
N(-10,25) vs N(-20,5)
N(-10,30) vs N(-20,5)
N(0,10) vs N(-10,5)
N(0,15) vs N(-10,5)
N(0,20) vs N(-10,5)
N(0,25) vs N(-10,5)
N(0,30) vs N(-10,5)
N(10,10) vs N(0,5)
N(10,15) vs N(0,5)
N(10,20) vs N(0,5)
N(10,25) vs N(0,5)
N(10,30) vs N(0,5)
N(20,10) vs N(10,5)
N(20,15) vs N(10,5)
N(20,20) vs N(10,5)
N(20,25) vs N(10,5)
N(20,30) vs N(10,5)
N(30,10) vs N(20,5)
N(30,15) vs N(20,5)
N(30,20) vs N(20,5)
N(30,25) vs N(20,5)
N(30,30) vs N(20,5)
N(40,10) vs N(30,5)
N(40,15) vs N(30,5)
N(40,20) vs N(30,5)
N(40,25) vs N(30,5)
N(40,30) vs N(30,5)
Parameter value
Generation
0.0
0.1
0.2
0.3
0.4
0.5
0
2500
5000

S4 Fig. Comprehensive display of evolutionary dynamics of $\boldsymbol{\beta}$ in 140 single-task simulations. The solid line and colored area are the mean value and SD, respectively, which were averaged over 10 simulations conducted with the same parameter setting. The column indicates the standard deviation of the risky option (σ_1). The row indicates the normal distribution of the safe option. Each panel corresponds to a single task. The task distribution is depicted by "risky option vs safe option" inside a panel.
