# Supplementary material for: Risk preference as an outcome of evolutionarily adaptive learning mechanisms: An evolutionary simulation under diverse risky environments
Source: PLoS One. 2024 Aug 1;19(8):e0307991. doi: 10.1371/journal.pone.0307991 (PMC11293680; doi:10.1371/journal.pone.0307991)
Supplement: S5 Fig — The white circle represents the mean value in the last generation (mean of 10 simulations with the same parameter setting). The vertical bar is ±1 standard deviation (mean of 10 simulations’ SD). In the positively located risk-seeking tasks, the lower mean value of β evolved to avoid being trapped in the less-rewarding option. In the other tasks, the mean β value evolved to reach higher values and allowed agents to behave in a greedy manner. The mean β value evolved to a higher value (consistently more than 0.35) in the risk-aversion task and when the distributions were in the negative region for the risk-seeking tasks. As the expected values of the two options did not change during learning, it seems reasonable that the agents tended to choose the more valuable option in a greedy manner. However, when the distributions were in the positive region during the risk-seeking tasks, the mean β evolved to a lower value. In these tasks, if agents with higher β initially choose the safe option, even the small positive outcome could greatly increase the probability of choosing the safe option. This leads agents to get trapped in the less-rewarding safe option. Hence agents with lower β (those who choose an option in a more random way) were more successful by avoiding safe option in the risk-seeking task. (PDF) [file pone.0307991.s009.pdf]

Risk-aversion task (D = -20)

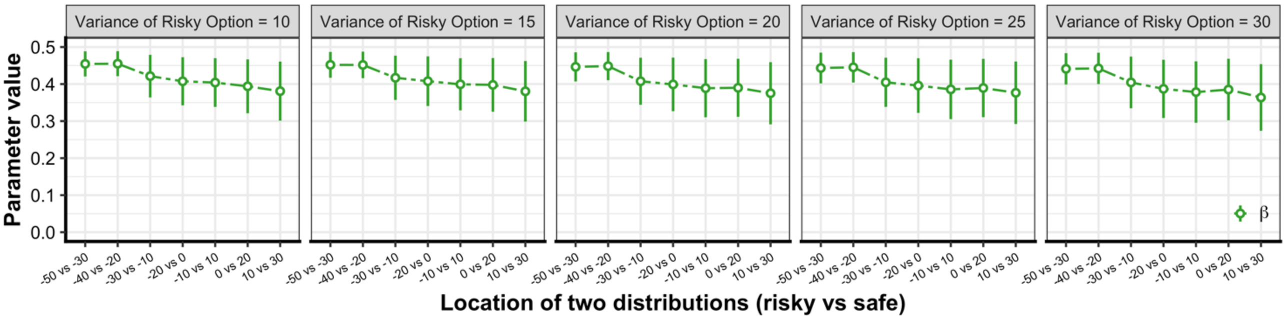

Risk-aversion task (D = -10)

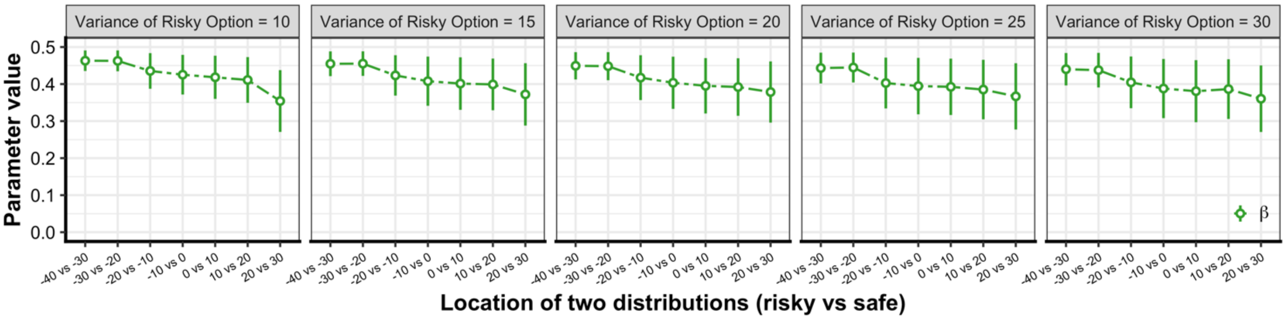

Risk-seeking task (D = +20)

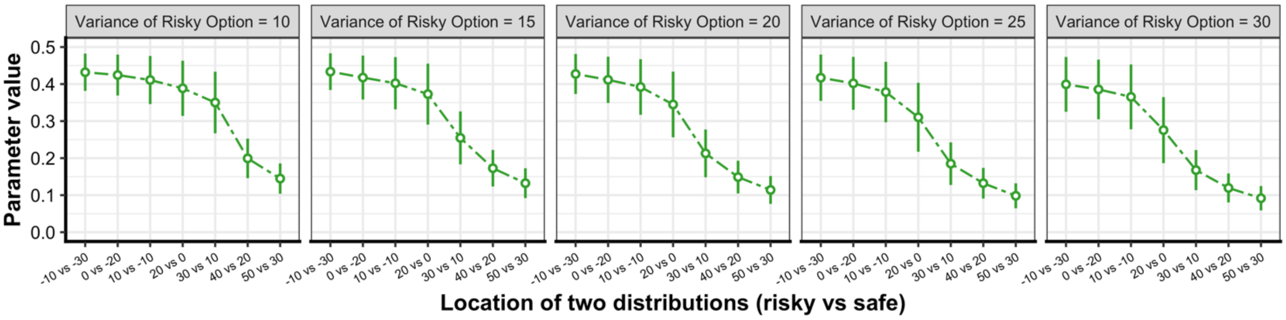

Risk-seeking task (D = +10)

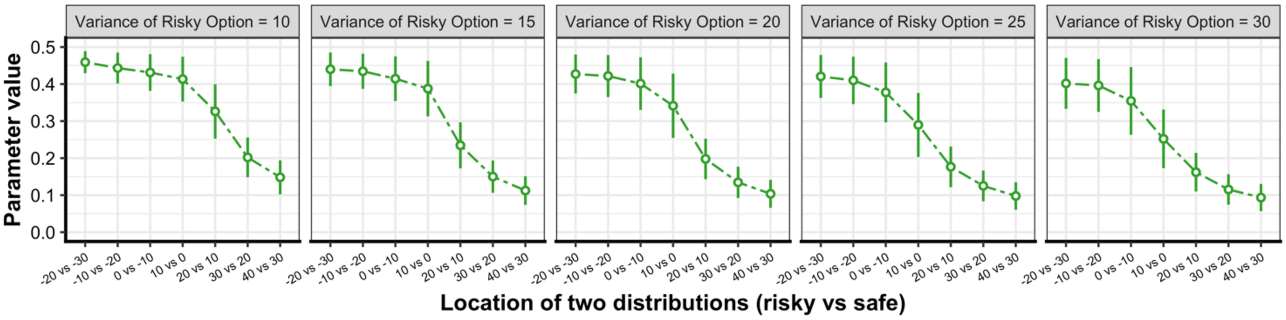

S5 Fig. Evolutionary result of β in 140 single tasks. The white circle represents the mean value in the last generation (mean of 10 simulations with the same parameter setting). The vertical bar is ±1 standard deviation (mean of 10 simulations' SD). In the positively located risk-seeking tasks, the lower mean value of β evolved to avoid being trapped in the less-rewarding option. In the other tasks, the mean β value evolved to reach higher values and allowed agents to behave in a greedy manner. The mean β value evolved to a higher value (consistently more than 0.35) in the risk-aversion task and when the distributions were in the negative region for the risk-seeking tasks. As the expected values of the two options did not change during learning, it seems reasonable that the agents tended to choose the more valuable option in a greedy manner. However, when the distributions were in the positive region during the risk-seeking tasks, the mean β evolved to a lower value. In these tasks, if agents with higher β initially choose the safe option, even the small positive outcome could greatly increase the probability of choosing the safe option. This leads agents to get trapped in the less-rewarding safe option. Hence agents with lower β (those who choose an option in a more random way) were more successful by avoiding safe option in the risk-seeking task.
