# Supplementary material for: Risk preference as an outcome of evolutionarily adaptive learning mechanisms: An evolutionary simulation under diverse risky environments
Source: PLoS One. 2024 Aug 1;19(8):e0307991. doi: 10.1371/journal.pone.0307991 (PMC11293680; doi:10.1371/journal.pone.0307991)
Supplement: S6 Fig — Each panel represents a simulation condition. (PDF) [file pone.0307991.s010.pdf]

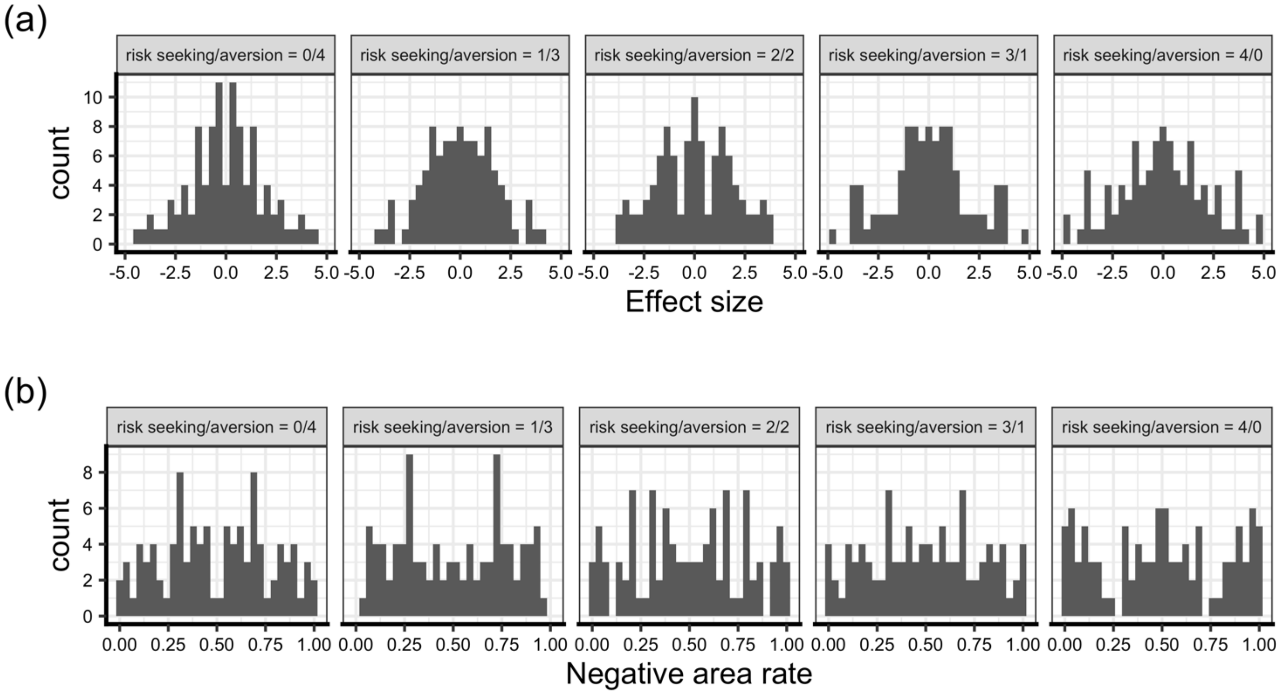

S6 Fig. Histogram of (a) the effect size and (b) negative area rate in a task group of a multiple-task simulation. Each panel represents a simulation condition.
