# Supplementary material for: Risk preference as an outcome of evolutionarily adaptive learning mechanisms: An evolutionary simulation under diverse risky environments
Source: PLoS One. 2024 Aug 1;19(8):e0307991. doi: 10.1371/journal.pone.0307991 (PMC11293680; doi:10.1371/journal.pone.0307991)
Supplement: S7 Fig — The horizontal axis indicates a task in a simulation depicted by the simulation number (from 1 to 100)–task number (from 1 to 4). The white circle and black circle represent the mean rate of risk aversion in the first and last generation, respectively. The vertical bar is ±1 SD. (PDF) [file pone.0307991.s011.pdf]

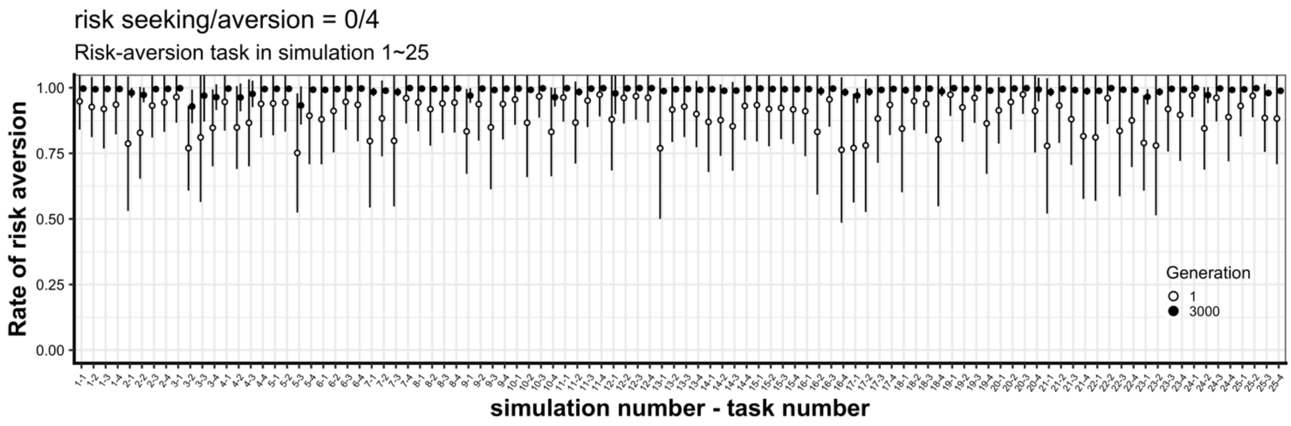
risk seeking/aversion = 0/4
Risk-aversion task in simulation 1~25
Rate of risk aversion
1.00
0.75
0.50
0.25
0.00
Generation
1
3000
simulation number - task number

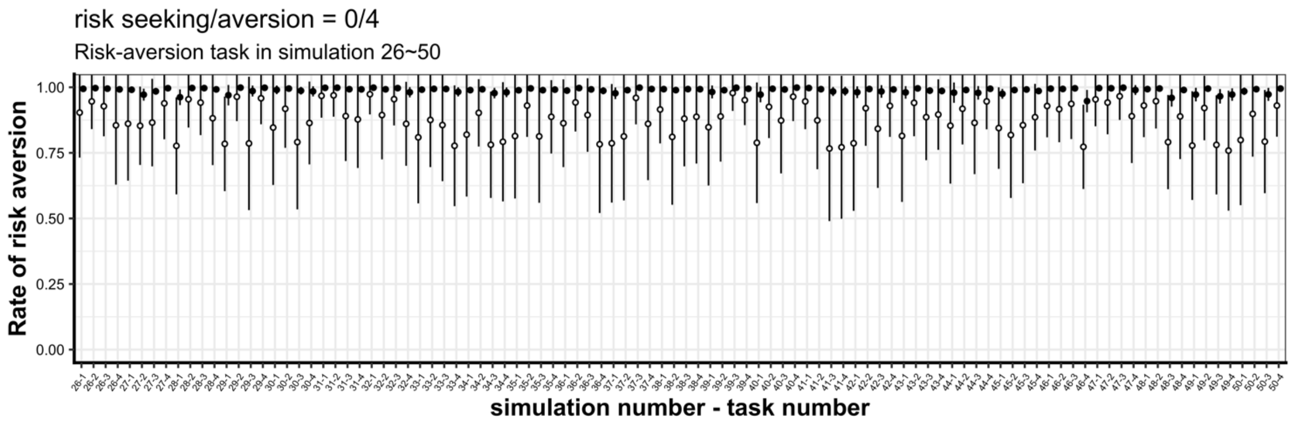
risk seeking/aversion = 0/4
Risk-aversion task in simulation 26~50
Rate of risk aversion
1.00
0.75
0.50
0.25
0.00
simulation number - task number

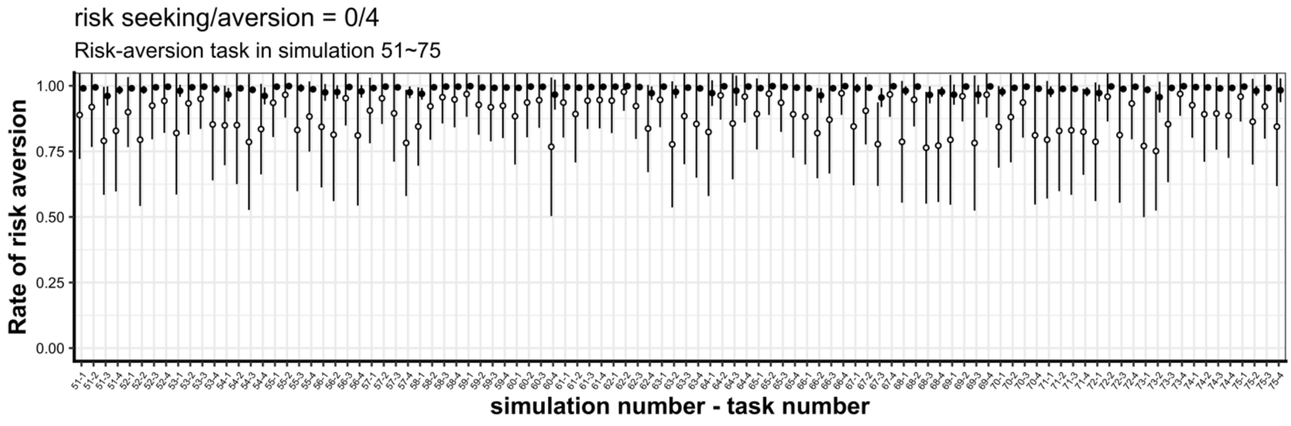
risk seeking/aversion = 0/4
Risk-aversion task in simulation 51~75
Rate of risk aversion
1.00
0.75
0.50
0.25
0.00
simulation number - task number

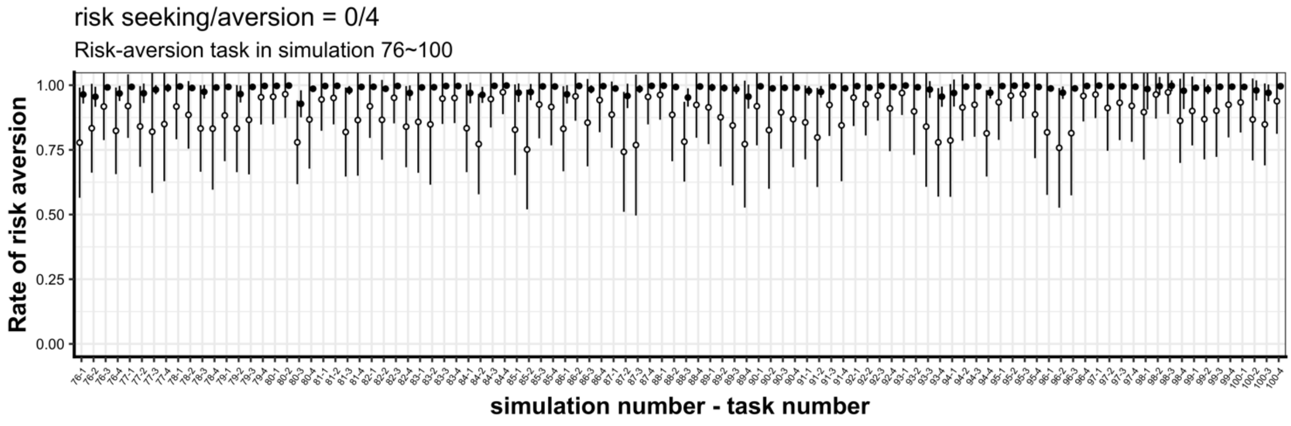
risk seeking/aversion = 0/4
Risk-aversion task in simulation 76~100
Rate of risk aversion
1.00
0.75
0.50
0.25
0.00
simulation number - task number

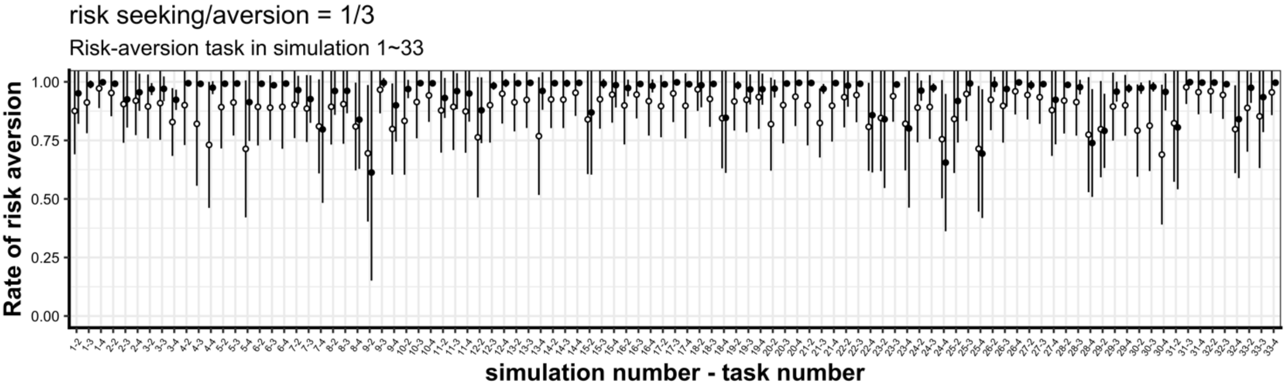
risk seeking/aversion = 1/3
Risk-aversion task in simulation 1~33
Rate of risk aversion
simulation number - task number

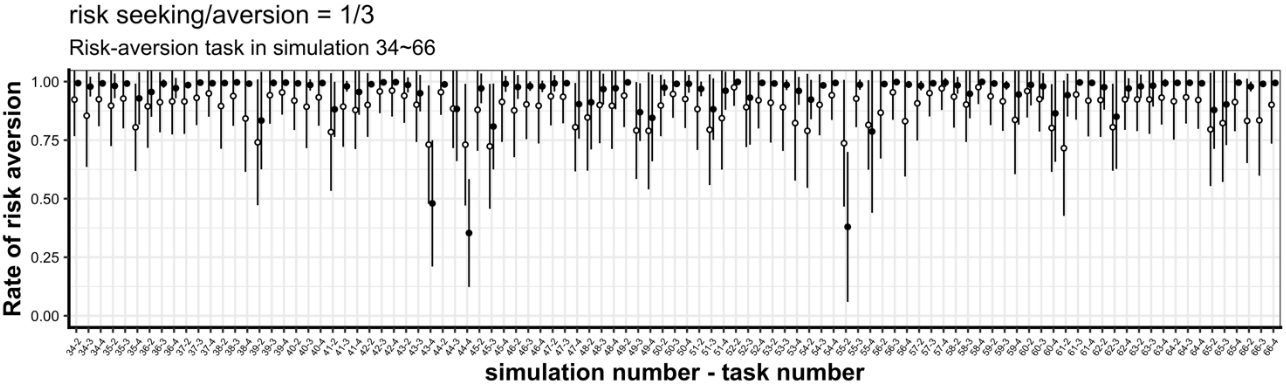
risk seeking/aversion = 1/3
Risk-aversion task in simulation 34~66
Rate of risk aversion
simulation number - task number

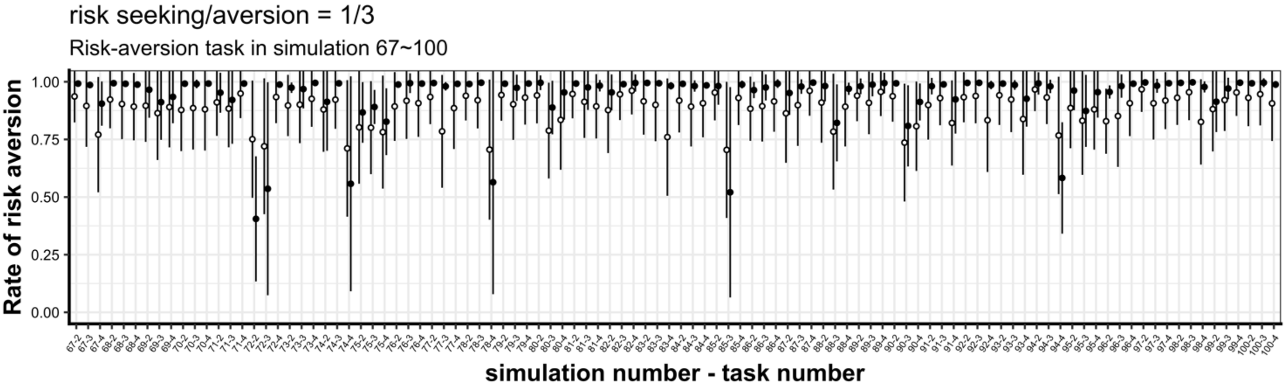
risk seeking/aversion = 1/3
Risk-aversion task in simulation 67~100
Rate of risk aversion
simulation number - task number

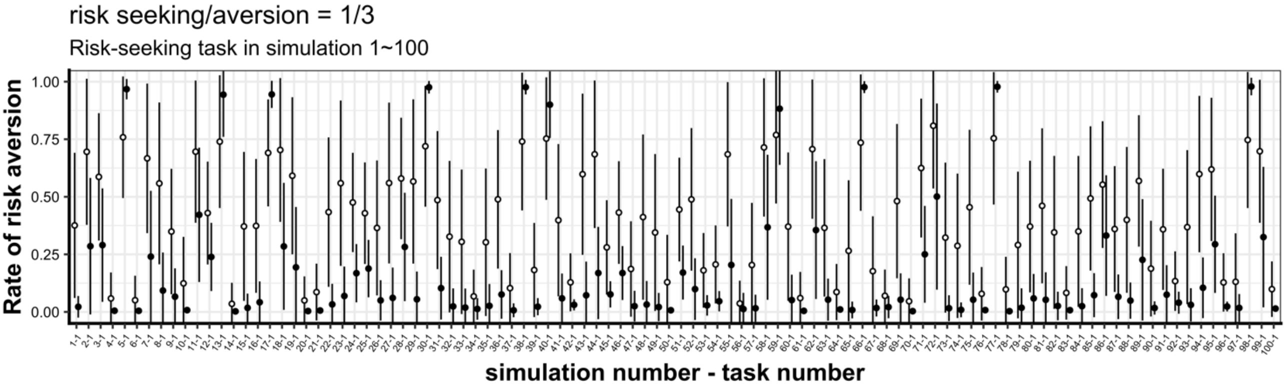
risk seeking/aversion = 1/3
Risk-seeking task in simulation 1~100
Rate of risk aversion
simulation number - task number

risk seeking/aversion = 2/2

Risk-aversion task in simulation 1~50

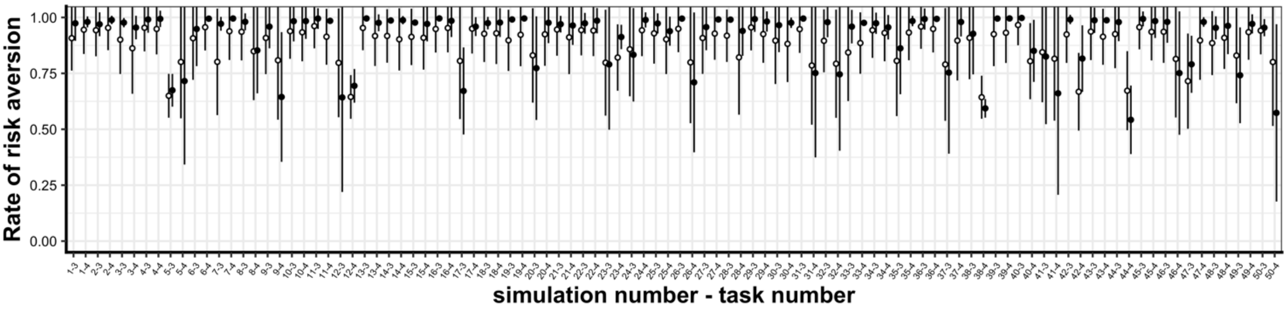

risk seeking/aversion = 2/2

Risk-aversion task in simulation 51~100

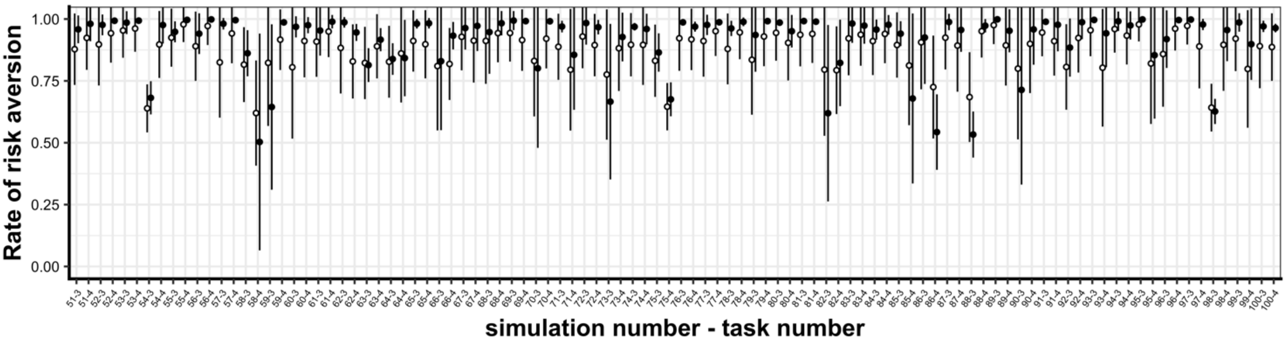

risk seeking/aversion = 2/2

Risk-seeking task in simulation 1~50

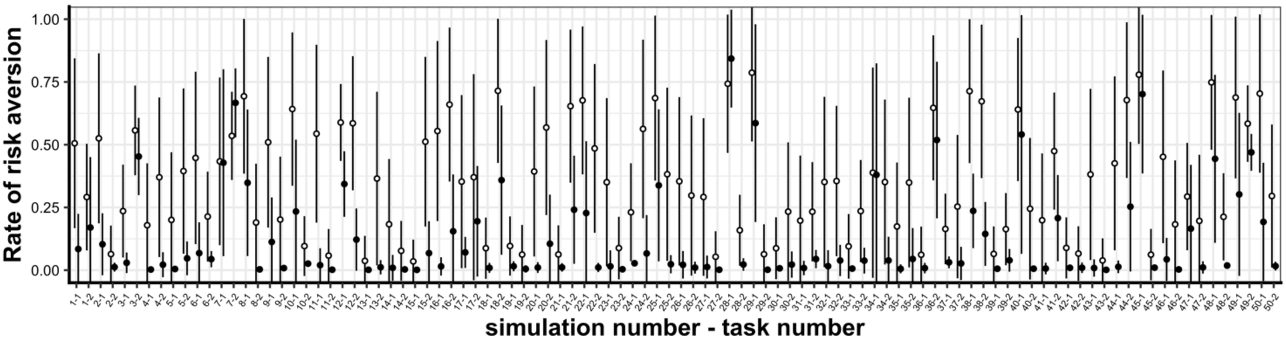

risk seeking/aversion = 2/2

Risk-seeking task in simulation 51~100

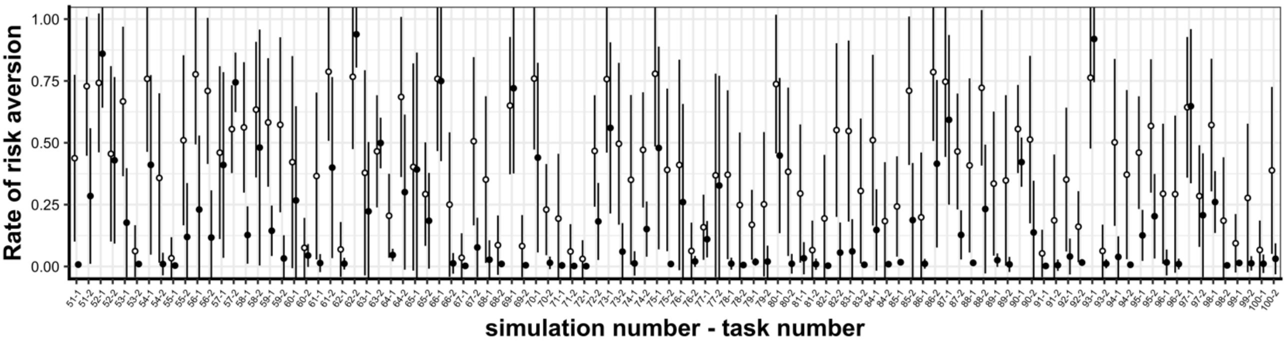

risk seeking/aversion = 3/1

Risk-aversion task in simulation 1~100

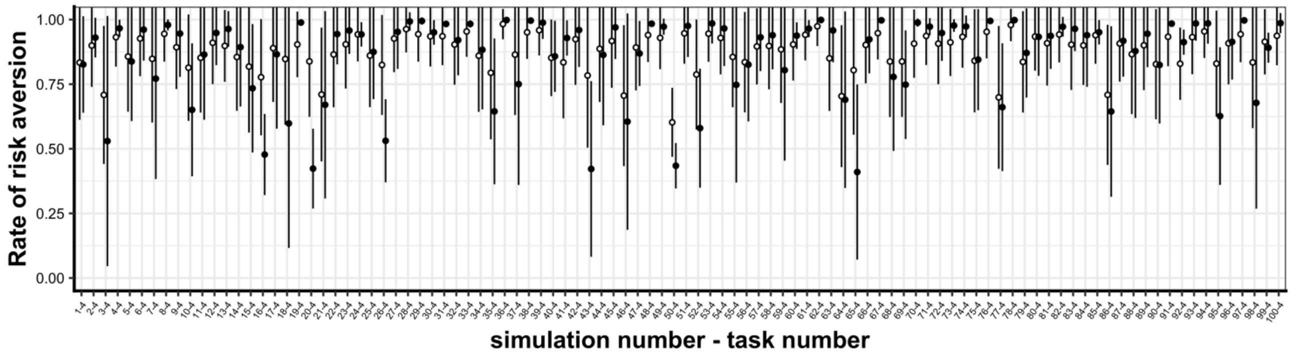

risk seeking/aversion = 3/1

Risk-seeking task in simulation 1~33

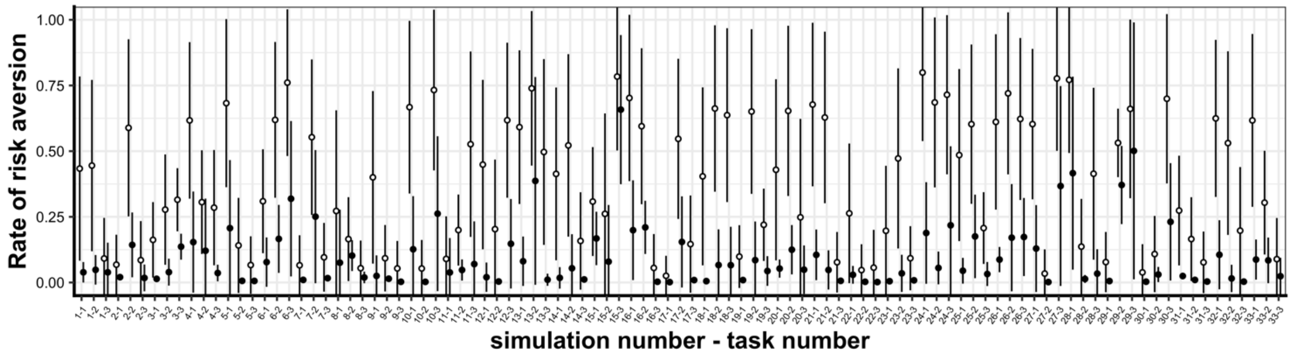

risk seeking/aversion = 3/1

Risk-seeking task in simulation 34~66

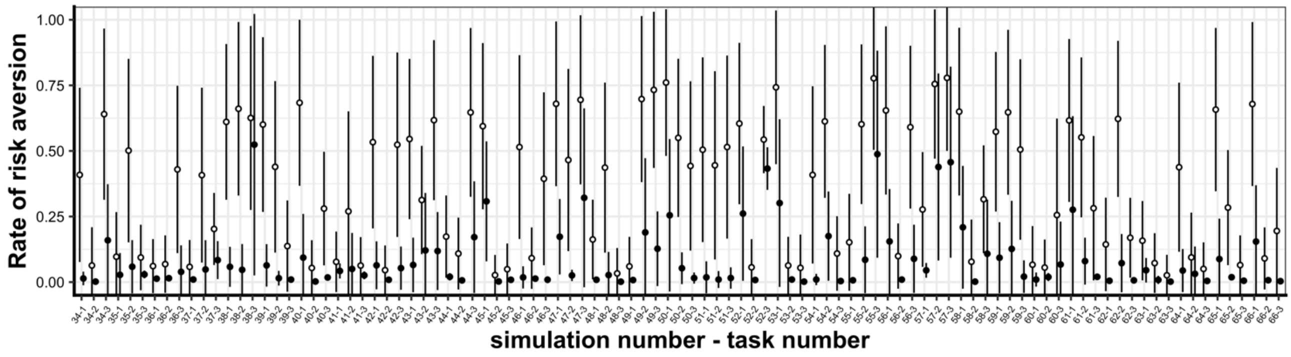

risk seeking/aversion = 3/1

Risk-seeking task in simulation 67~100

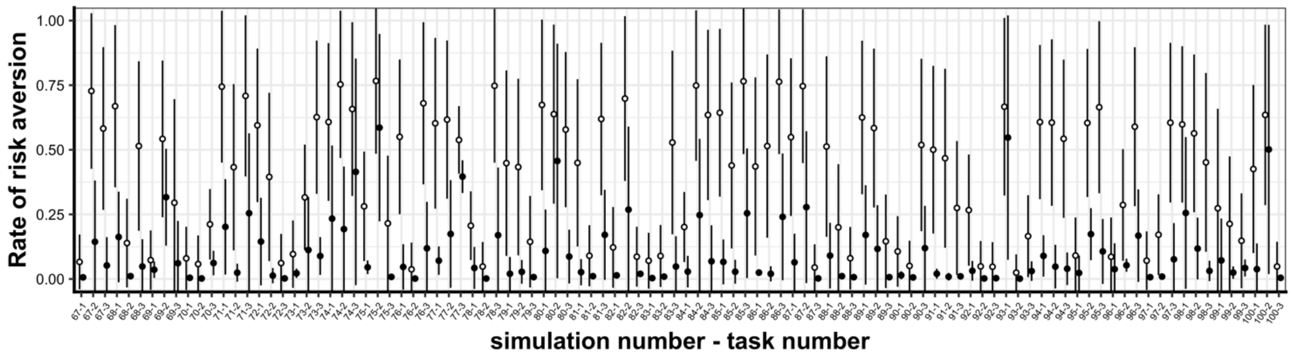

risk seeking/aversion = 4/0

Risk-seeking task in simulation 1~25

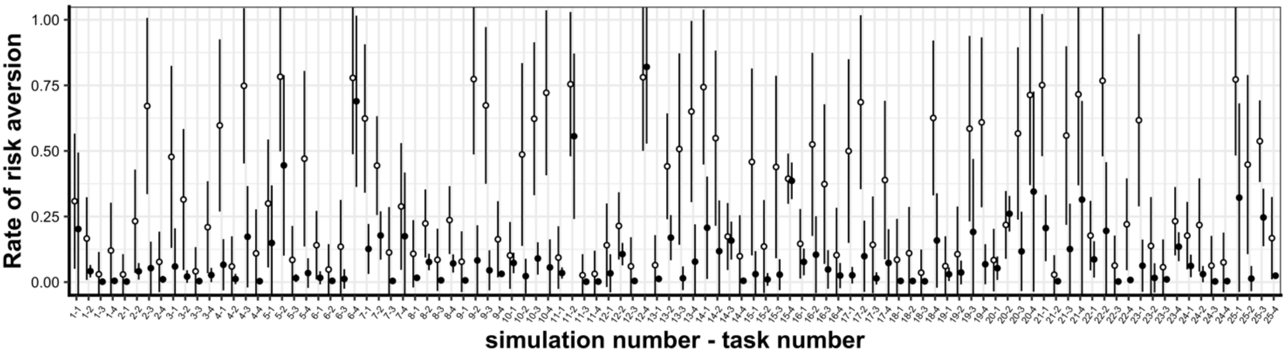

risk seeking/aversion = 4/0

Risk-seeking task in simulation 26~50

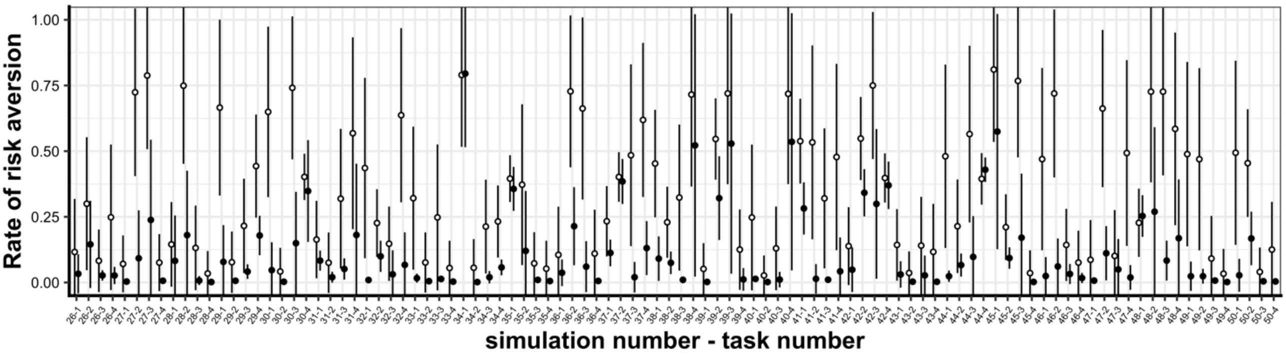

risk seeking/aversion = 4/0

Risk-seeking task in simulation 51~75

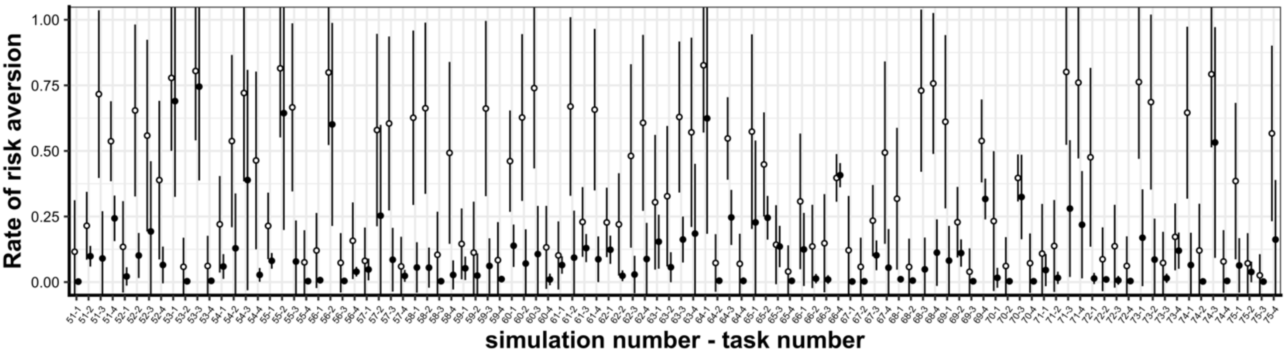

risk seeking/aversion = 4/0

Risk-seeking task in simulation 76~100

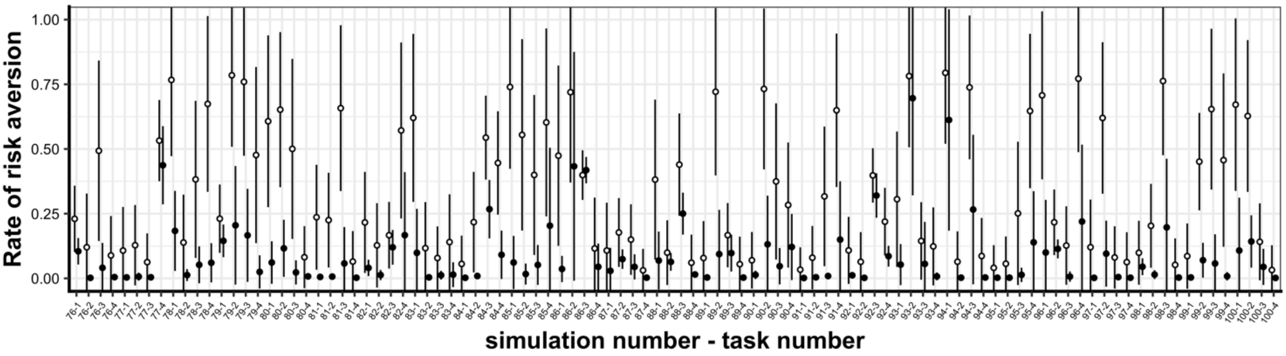

S7 Fig. Comprehensive display of the risk aversion rate of all tasks in multiple-task simulations. The horizontal axis indicates a task in a simulation depicted by the simulation number (from 1 to 100) – task number (from 1 to 4). The white circle and black circle represent the mean rate of risk aversion in the first and last generation, respectively. The vertical bar is ±1 SD.
