# Supplementary material for: Risk preference as an outcome of evolutionarily adaptive learning mechanisms: An evolutionary simulation under diverse risky environments
Source: PLoS One. 2024 Aug 1;19(8):e0307991. doi: 10.1371/journal.pone.0307991 (PMC11293680; doi:10.1371/journal.pone.0307991)
Supplement: S8 Fig — The solid line represents the mean rate of risk aversion. The colored area shows ±1 SD. The top (bottom) panel corresponds to the first (last) generation. The mean rate of choosing the more rewarding option increased in both the risk-aversion and risk-seeking tasks for the final generation. (PDF) [file pone.0307991.s012.pdf]

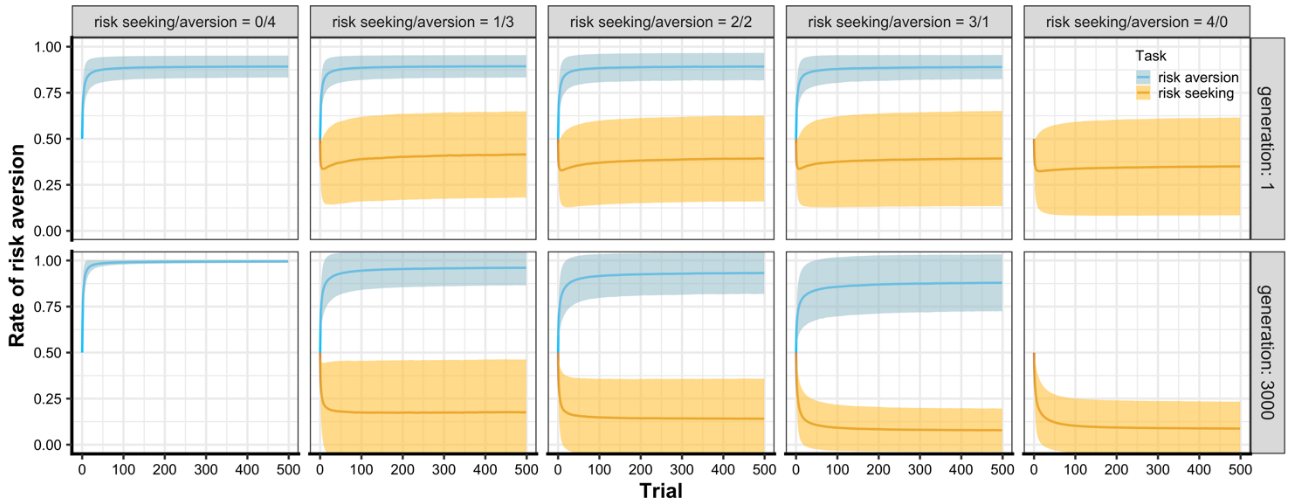

S8 Fig. Learning dynamics of the population in the multiple-task simulations as well as comparison of the first generation with the last generation. The solid line represents the mean rate of risk aversion. The colored area shows ±1 SD. The top (bottom) panel corresponds to the first (last) generation. The mean rate of choosing the more rewarding option increased in both the risk-aversion and risk-seeking tasks for the final generation.
