# Supplementary material for: Risk preference as an outcome of evolutionarily adaptive learning mechanisms: An evolutionary simulation under diverse risky environments
Source: PLoS One. 2024 Aug 1;19(8):e0307991. doi: 10.1371/journal.pone.0307991 (PMC11293680; doi:10.1371/journal.pone.0307991)
Supplement: S9 Fig — Each black line represents the mean value of the parameter of a simulation. The colored line shows the averaged value of the mean value of simulations. As the number of risk-seeking tasks increased, the potential to be trapped in the less-rewarding option increased, which led β evolving to lower in some simulations. The mean β increased when the number of risk-seeking task was small, and it showed more variability as the number of risk-seeking tasks increased. This evolutionary pattern can be explained by the same logic as that used in the single-task simulation. When the number of risk-seeking tasks was small, the mean value of β evolved to be higher (led agents to behave in the greedy way). However, as it increased, the chances that agents got trapped in the less-rewarding safe option also increased; therefore, β evolved to be lower in some simulations (led agents to behave in the random way). (PDF) [file pone.0307991.s013.pdf]

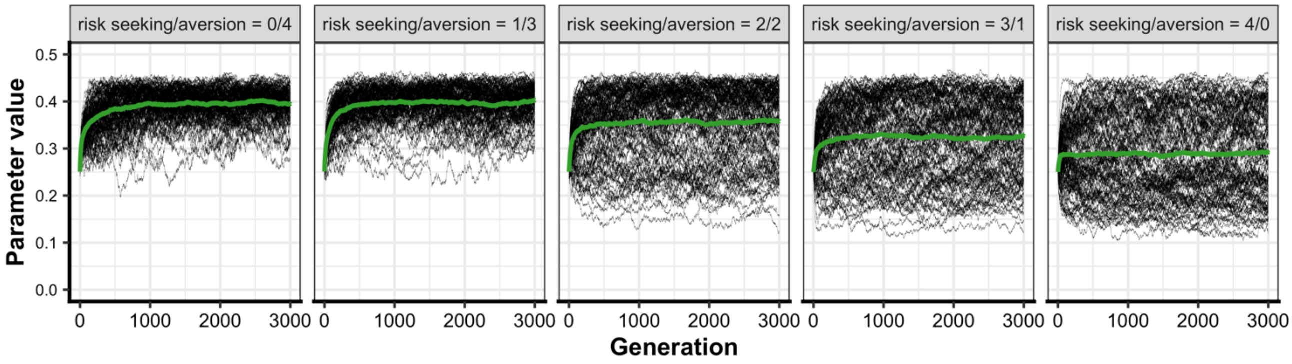

S9 Fig. Evolutionary dynamics of β in 100 multiple-task simulations. Each black line represents the mean value of the parameter of a simulation. The colored line shows the averaged value of the mean value of simulations. As the number of risk-seeking tasks increased, the potential to be trapped in the less-rewarding option increased, which led β evolving to lower in some simulations. The mean β increased when the number of risk-seeking task was small, and it showed more variability as the number of risk-seeking tasks increased. This evolutionary pattern can be explained by the same logic as that used in the single-task simulation. When the number of risk-seeking tasks was small, the mean value of β evolved to be higher (led agents to behave in the greedy way). However, as it increased, the chances that agents got trapped in the less-rewarding safe option also increased; therefore, β evolved to be lower in some simulations (led agents to behave in the random way).
