# Supplementary material for: Risk preference as an outcome of evolutionarily adaptive learning mechanisms: An evolutionary simulation under diverse risky environments
Source: PLoS One. 2024 Aug 1;19(8):e0307991. doi: 10.1371/journal.pone.0307991 (PMC11293680; doi:10.1371/journal.pone.0307991)
Supplement: S10 Fig — The column indicates the risk of the risky option (σ1). The row indicates the normal distribution of the safe option. Each panel corresponds to a single task. The task distribution is depicted by “risky option vs safe option” inside a panel. The star (*) following the task distribution indicates that the panel was shown in Fig 3 in the main text. (PDF) [file pone.0307991.s014.pdf]

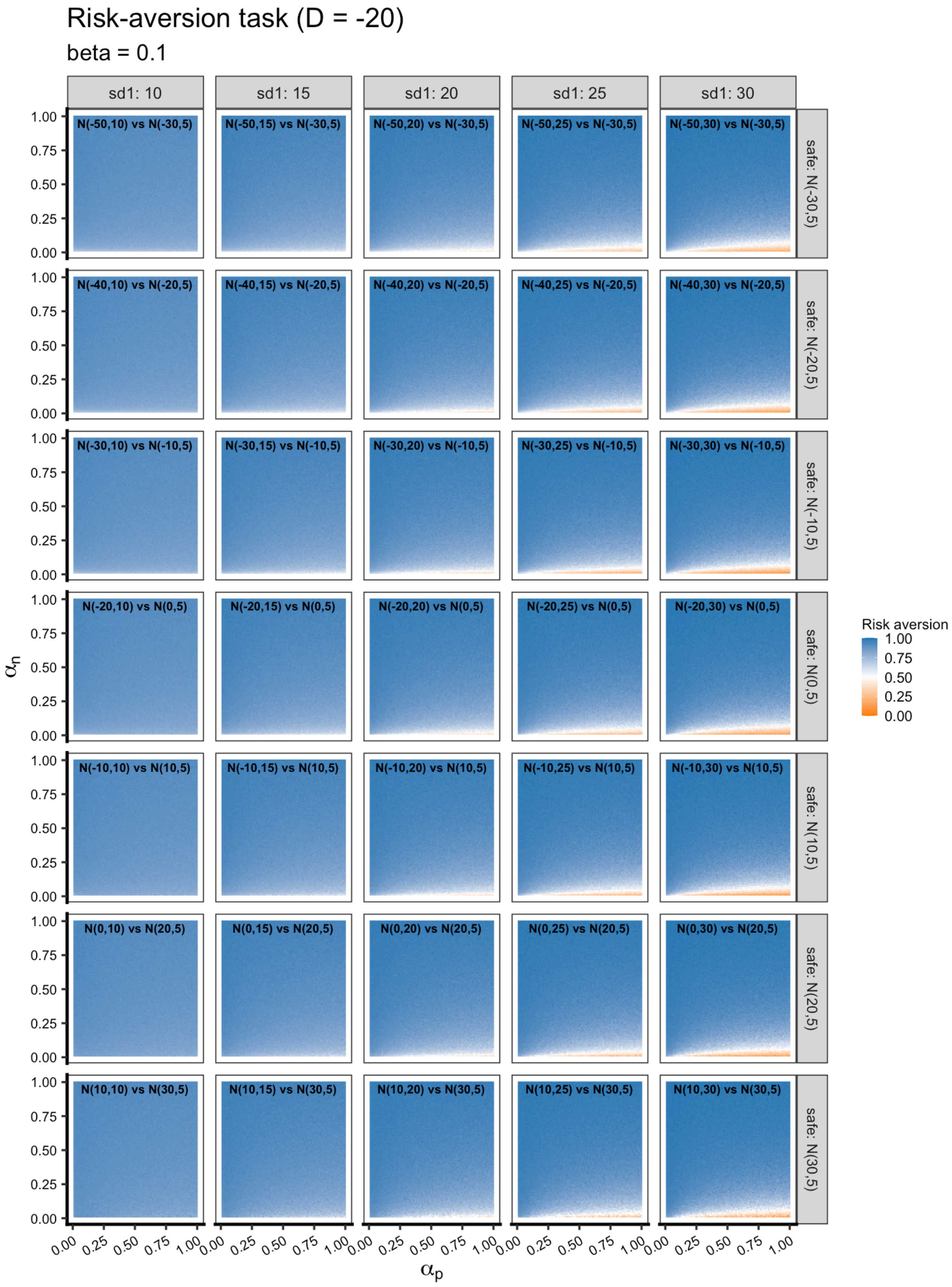

Risk-aversion task (D = -20)
beta = 0.1
sd1: 10
sd1: 15
sd1: 20
sd1: 25
sd1: 30
safe: N(-30,5)
safe: N(-20,5)
safe: N(-10,5)
safe: N(0,5)
safe: N(10,5)
safe: N(20,5)
safe: N(30,5)
N(-50,10) vs N(-30,5)
N(-50,15) vs N(-30,5)
N(-50,20) vs N(-30,5)
N(-50,25) vs N(-30,5)
N(-50,30) vs N(-30,5)
N(-40,10) vs N(-20,5)
N(-40,15) vs N(-20,5)
N(-40,20) vs N(-20,5)
N(-40,25) vs N(-20,5)
N(-40,30) vs N(-20,5)
N(-30,10) vs N(-10,5)
N(-30,15) vs N(-10,5)
N(-30,20) vs N(-10,5)
N(-30,25) vs N(-10,5)
N(-30,30) vs N(-10,5)
N(-20,10) vs N(0,5)
N(-20,15) vs N(0,5)
N(-20,20) vs N(0,5)
N(-20,25) vs N(0,5)
N(-20,30) vs N(0,5)
N(-10,10) vs N(10,5)
N(-10,15) vs N(10,5)
N(-10,20) vs N(10,5)
N(-10,25) vs N(10,5)
N(-10,30) vs N(10,5)
N(0,10) vs N(20,5)
N(0,15) vs N(20,5)
N(0,20) vs N(20,5)
N(0,25) vs N(20,5)
N(0,30) vs N(20,5)
N(10,10) vs N(30,5)
N(10,15) vs N(30,5)
N(10,20) vs N(30,5)
N(10,25) vs N(30,5)
N(10,30) vs N(30,5)
α_n
α_p
0.00
0.25
0.50
0.75
1.00
Risk aversion
1.00
0.75
0.50
0.25
0.00

Risk-aversion task (D = -20)

beta = 0.25

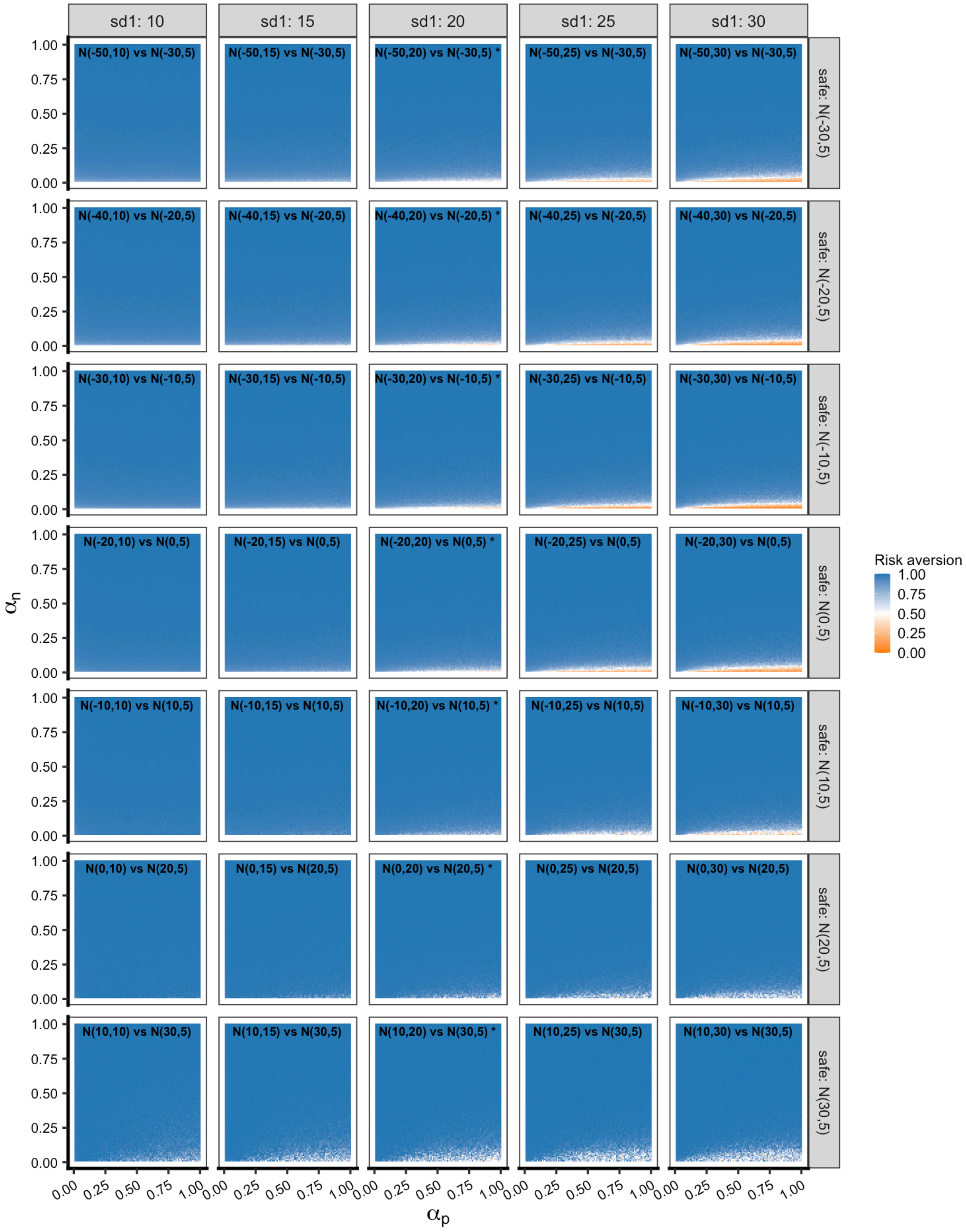

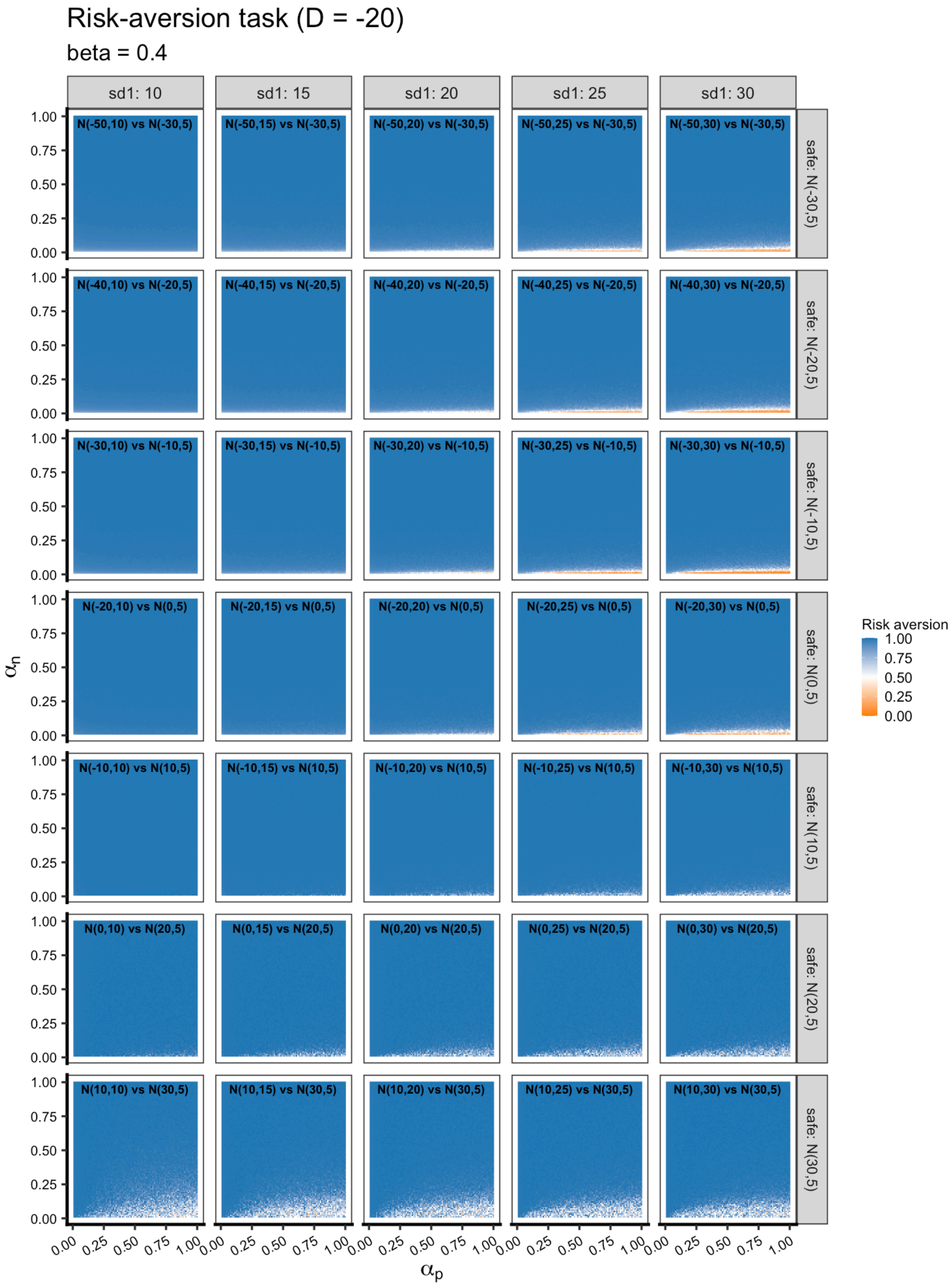

Risk-aversion task (D = -20)
beta = 0.4
sd1: 10
sd1: 15
sd1: 20
sd1: 25
sd1: 30
N(-50,10) vs N(-30,5)
N(-50,15) vs N(-30,5)
N(-50,20) vs N(-30,5)
N(-50,25) vs N(-30,5)
N(-50,30) vs N(-30,5)
safe: N(-30,5)
N(-40,10) vs N(-20,5)
N(-40,15) vs N(-20,5)
N(-40,20) vs N(-20,5)
N(-40,25) vs N(-20,5)
N(-40,30) vs N(-20,5)
safe: N(-20,5)
N(-30,10) vs N(-10,5)
N(-30,15) vs N(-10,5)
N(-30,20) vs N(-10,5)
N(-30,25) vs N(-10,5)
N(-30,30) vs N(-10,5)
safe: N(-10,5)
N(-20,10) vs N(0,5)
N(-20,15) vs N(0,5)
N(-20,20) vs N(0,5)
N(-20,25) vs N(0,5)
N(-20,30) vs N(0,5)
safe: N(0,5)
N(-10,10) vs N(10,5)
N(-10,15) vs N(10,5)
N(-10,20) vs N(10,5)
N(-10,25) vs N(10,5)
N(-10,30) vs N(10,5)
safe: N(10,5)
N(0,10) vs N(20,5)
N(0,15) vs N(20,5)
N(0,20) vs N(20,5)
N(0,25) vs N(20,5)
N(0,30) vs N(20,5)
safe: N(20,5)
N(10,10) vs N(30,5)
N(10,15) vs N(30,5)
N(10,20) vs N(30,5)
N(10,25) vs N(30,5)
N(10,30) vs N(30,5)
safe: N(30,5)
α_n
α_p
0.00
0.25
0.50
0.75
1.00
Risk aversion
1.00
0.75
0.50
0.25
0.00

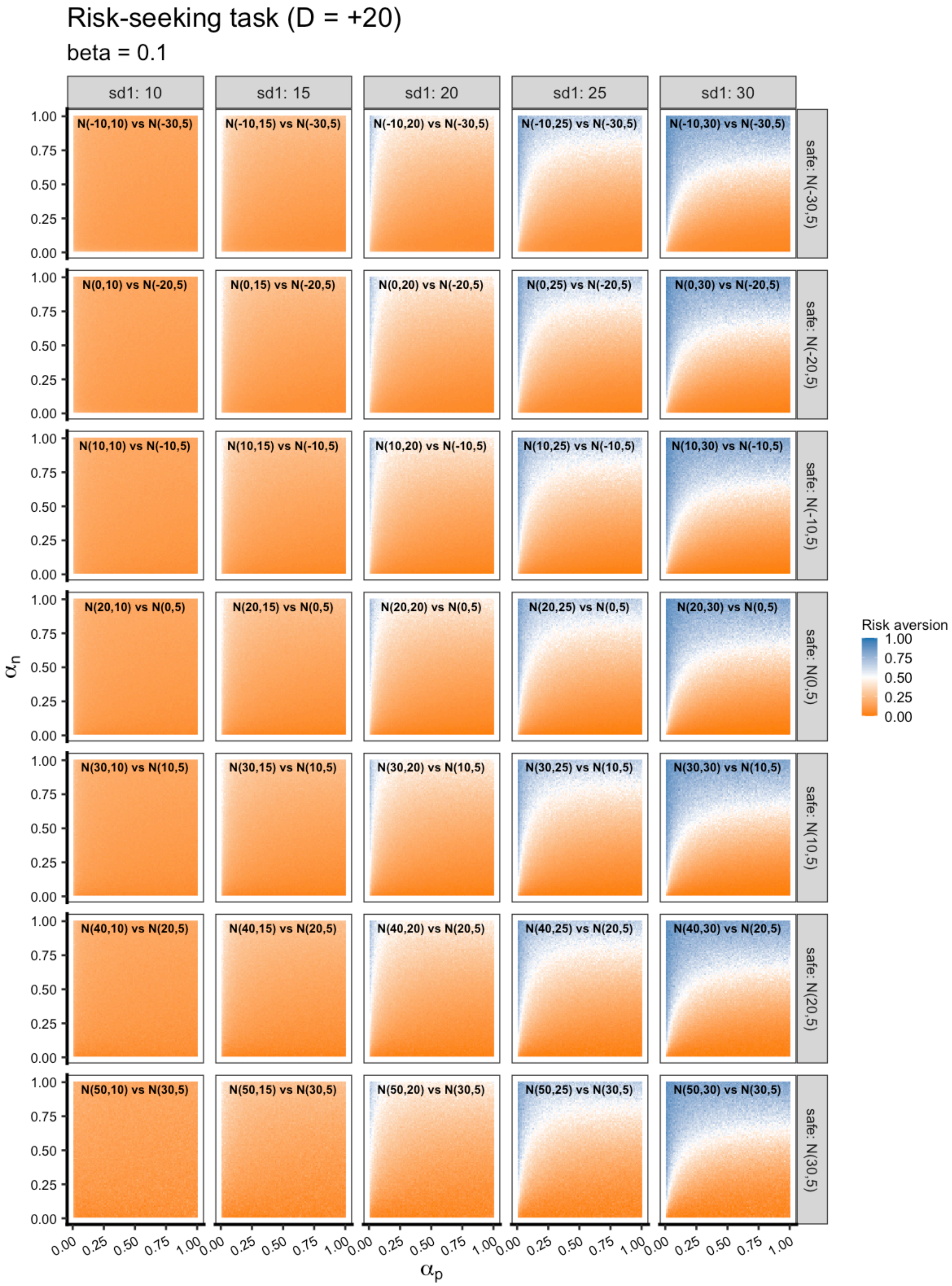
Risk-seeking task (D = +20)
beta = 0.1
sd1: 10
sd1: 15
sd1: 20
sd1: 25
sd1: 30
safe: N(-30,5)
safe: N(-20,5)
safe: N(-10,5)
safe: N(0,5)
safe: N(10,5)
safe: N(20,5)
safe: N(30,5)
N(-10,10) vs N(-30,5)
N(-10,15) vs N(-30,5)
N(-10,20) vs N(-30,5)
N(-10,25) vs N(-30,5)
N(-10,30) vs N(-30,5)
N(0,10) vs N(-20,5)
N(0,15) vs N(-20,5)
N(0,20) vs N(-20,5)
N(0,25) vs N(-20,5)
N(0,30) vs N(-20,5)
N(10,10) vs N(-10,5)
N(10,15) vs N(-10,5)
N(10,20) vs N(-10,5)
N(10,25) vs N(-10,5)
N(10,30) vs N(-10,5)
N(20,10) vs N(0,5)
N(20,15) vs N(0,5)
N(20,20) vs N(0,5)
N(20,25) vs N(0,5)
N(20,30) vs N(0,5)
N(30,10) vs N(10,5)
N(30,15) vs N(10,5)
N(30,20) vs N(10,5)
N(30,25) vs N(10,5)
N(30,30) vs N(10,5)
N(40,10) vs N(20,5)
N(40,15) vs N(20,5)
N(40,20) vs N(20,5)
N(40,25) vs N(20,5)
N(40,30) vs N(20,5)
N(50,10) vs N(30,5)
N(50,15) vs N(30,5)
N(50,20) vs N(30,5)
N(50,25) vs N(30,5)
N(50,30) vs N(30,5)
α_n
α_p
0.00
0.25
0.50
0.75
1.00
Risk aversion
1.00
0.75
0.50
0.25
0.00

Risk-seeking task (D = +20)

beta = 0.25

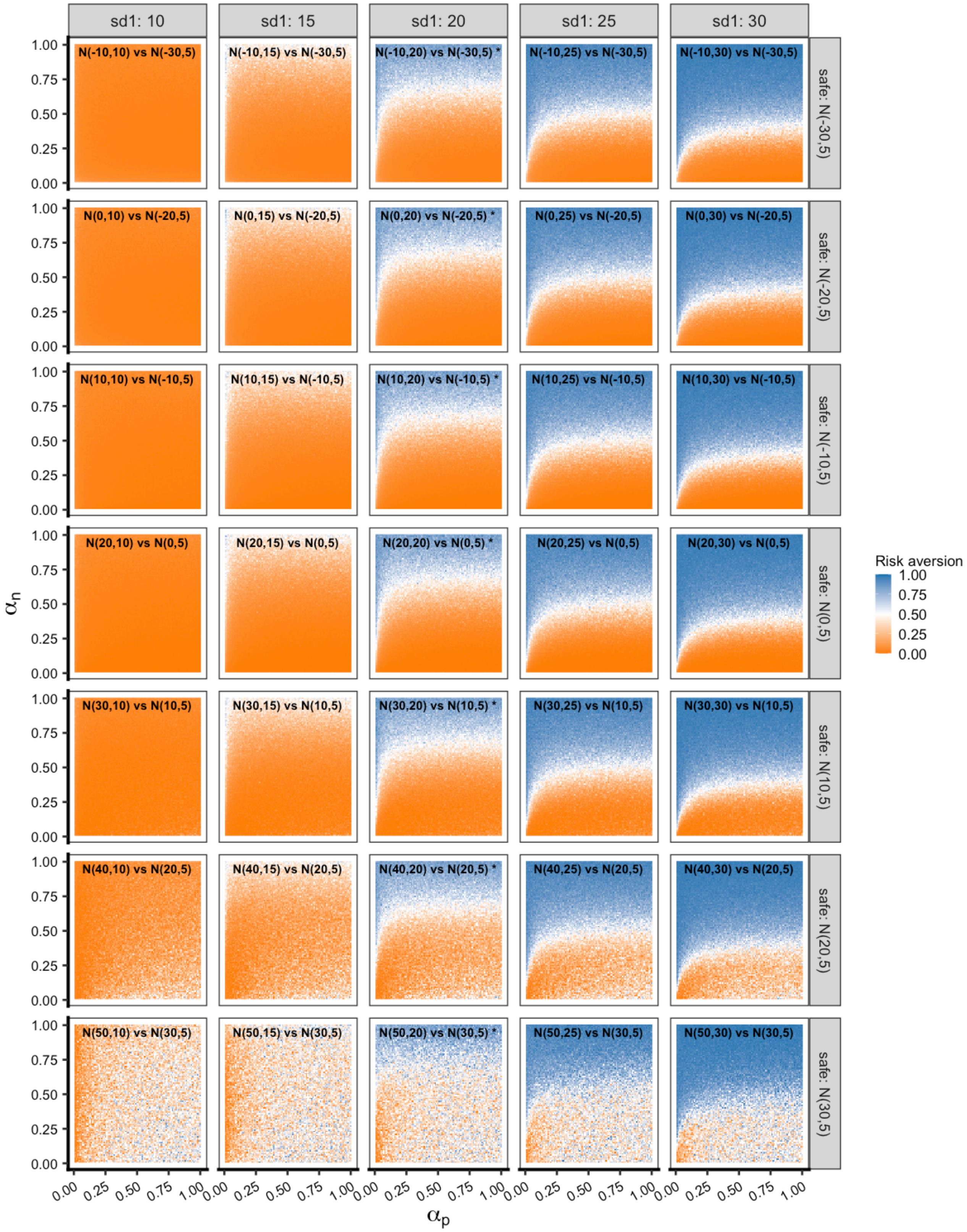

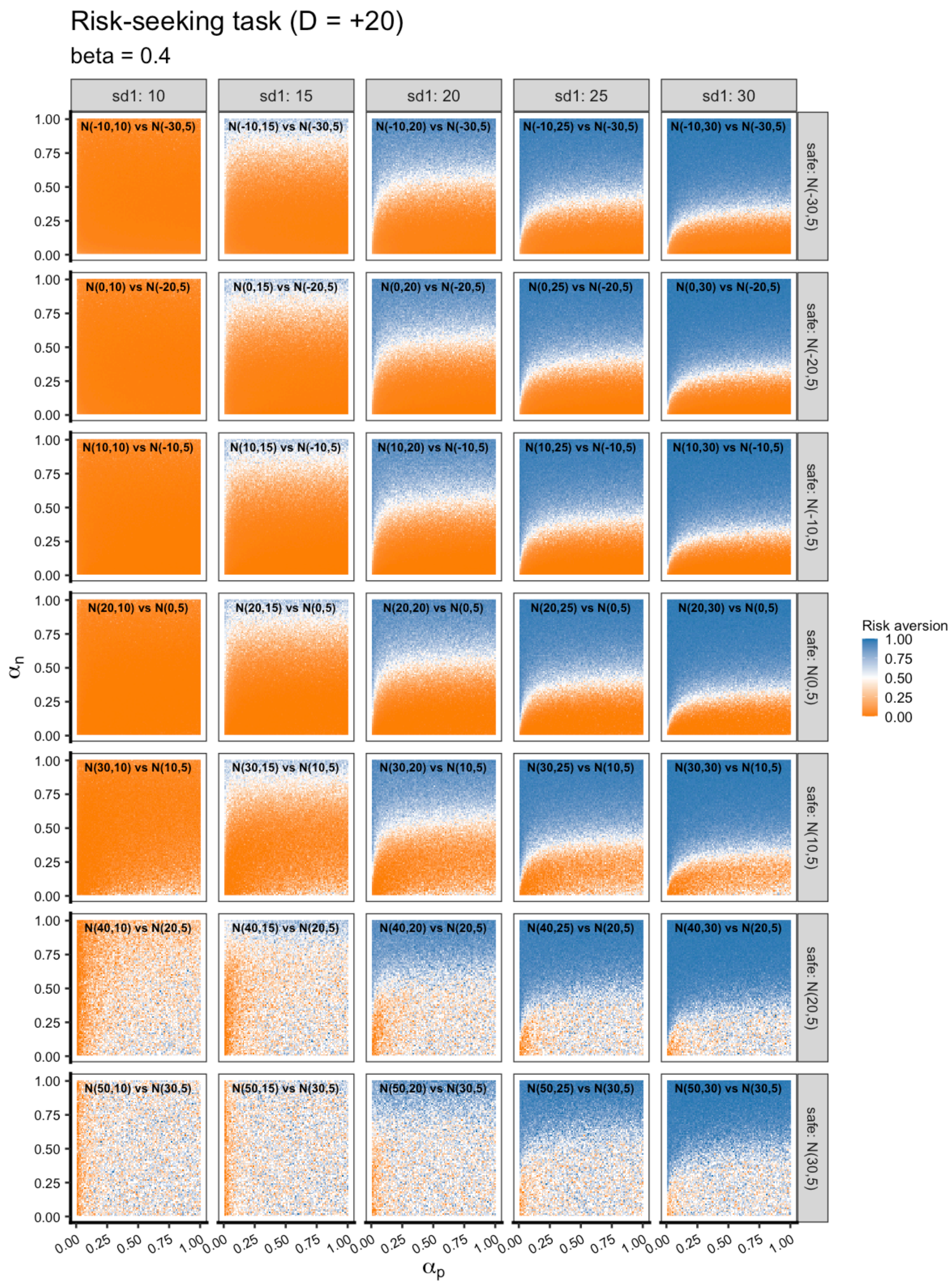

Risk-seeking task (D = +20)
beta = 0.4
sd1: 10
sd1: 15
sd1: 20
sd1: 25
sd1: 30
N(-10,10) vs N(-30,5)
N(-10,15) vs N(-30,5)
N(-10,20) vs N(-30,5)
N(-10,25) vs N(-30,5)
N(-10,30) vs N(-30,5)
safe: N(-30,5)
N(0,10) vs N(-20,5)
N(0,15) vs N(-20,5)
N(0,20) vs N(-20,5)
N(0,25) vs N(-20,5)
N(0,30) vs N(-20,5)
safe: N(-20,5)
N(10,10) vs N(-10,5)
N(10,15) vs N(-10,5)
N(10,20) vs N(-10,5)
N(10,25) vs N(-10,5)
N(10,30) vs N(-10,5)
safe: N(-10,5)
N(20,10) vs N(0,5)
N(20,15) vs N(0,5)
N(20,20) vs N(0,5)
N(20,25) vs N(0,5)
N(20,30) vs N(0,5)
safe: N(0,5)
N(30,10) vs N(10,5)
N(30,15) vs N(10,5)
N(30,20) vs N(10,5)
N(30,25) vs N(10,5)
N(30,30) vs N(10,5)
safe: N(10,5)
N(40,10) vs N(20,5)
N(40,15) vs N(20,5)
N(40,20) vs N(20,5)
N(40,25) vs N(20,5)
N(40,30) vs N(20,5)
safe: N(20,5)
N(50,10) vs N(30,5)
N(50,15) vs N(30,5)
N(50,20) vs N(30,5)
N(50,25) vs N(30,5)
N(50,30) vs N(30,5)
safe: N(30,5)
α_n
α_p
0.00
0.25
0.50
0.75
1.00
Risk aversion
1.00
0.75
0.50
0.25
0.00

S10 Fig. Comprehensive display of the effects of α_p and α_n on risky behavior in the 70 single-task simulations when β was fixed to 0.1, 0.25, or 0.4. The column indicates the risk of the risky option (σ_1). The row indicates the normal distribution of the safe option. Each panel corresponds to a single task. The task distribution is depicted by "risky option vs safe option" inside a panel. The star (*) following the task distribution indicates that the panel was shown in Fig 3 in the main text.
