# Supplementary material for: Risk preference as an outcome of evolutionarily adaptive learning mechanisms: An evolutionary simulation under diverse risky environments
Source: PLoS One. 2024 Aug 1;19(8):e0307991. doi: 10.1371/journal.pone.0307991 (PMC11293680; doi:10.1371/journal.pone.0307991)
Supplement: S11 Fig — (a) Frequency of agents’ risk aversion rate in the initial generation of multiple-task simulations when the expected value of two options was the same. The rate of risk aversion was calculated from the last 100-trial choice of 500 trials. Because the initial genes were randomly assigned from a uniform distribution, the histograms were almost identical across simulation conditions. Furthermore, regardless of the gain or loss domain, a peak was observed for complete risk aversion. (b) The frequency of the difference in risk aversion rate between the gain domain and loss domain in the initial agents. A high peak in the difference was observed at approximately zero; thus, indicating that numerous agents slightly changed their degree of risk aversion. The number of agents who increased their risk aversion in the gain domain was small compared with those in the last generation. (PDF) [file pone.0307991.s015.pdf]

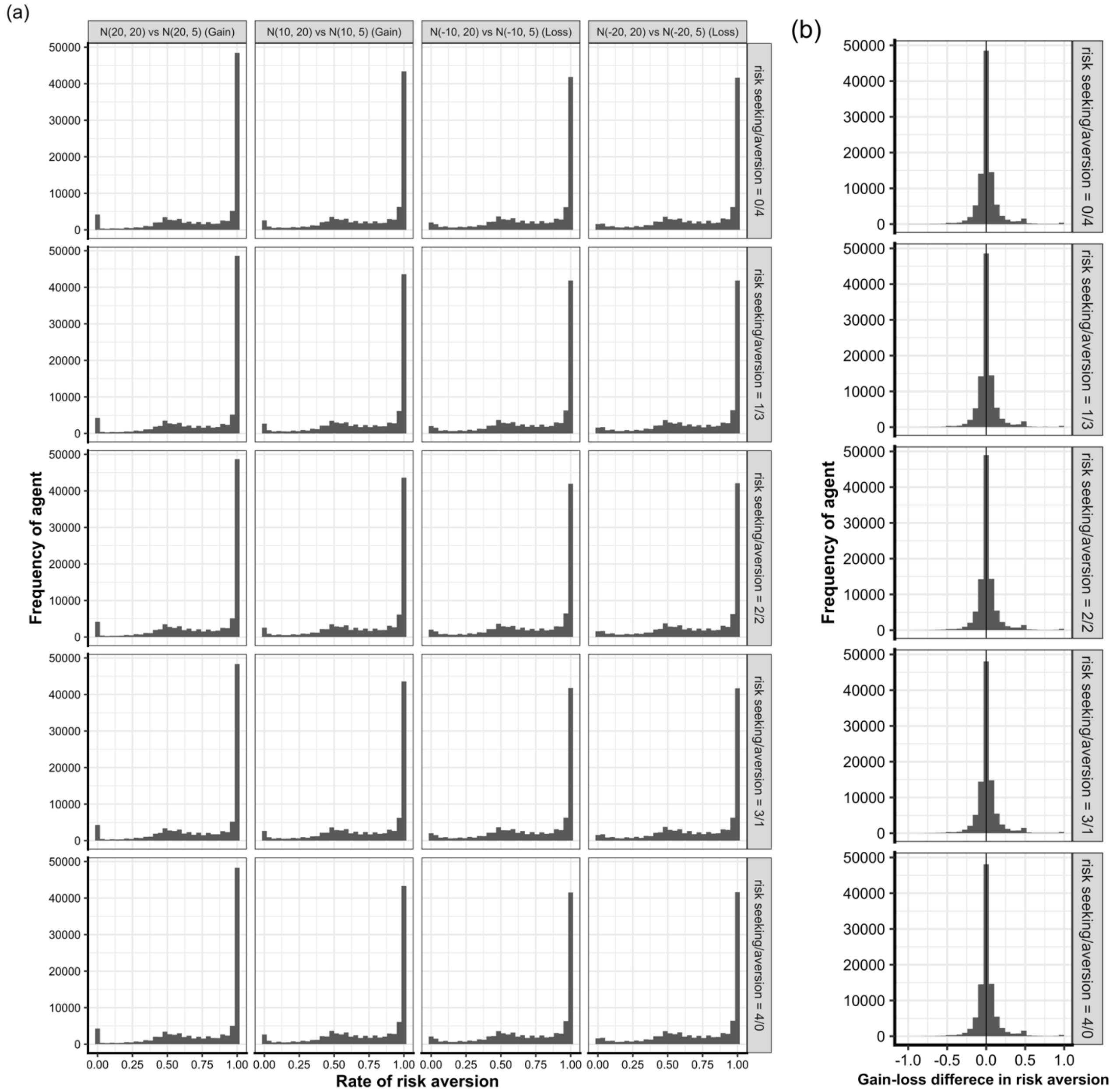

S11 Fig. Risk tendency of evolved agents in the tasks where the expected values are the same. (a) Frequency of agents' risk aversion rate in the initial generation of multiple-task simulations when the expected value of two options was the same. The rate of risk aversion was calculated from the last 100-trial choice of 500 trials. Because the initial genes were randomly assigned from a uniform distribution, the histograms were almost identical across simulation conditions. Furthermore, regardless of the gain or loss domain, a peak was observed for complete risk aversion. (b) The frequency of the difference in risk aversion rate between the gain domain and loss domain in the initial agents. A high peak in the difference was observed at approximately

zero; thus, indicating that numerous agents slightly changed their degree of risk aversion. The number of agents who increased their risk aversion in the gain domain was small compared with those in the last generation.
