# Supplementary material for: Risk preference as an outcome of evolutionarily adaptive learning mechanisms: An evolutionary simulation under diverse risky environments
Source: PLoS One. 2024 Aug 1;19(8):e0307991. doi: 10.1371/journal.pone.0307991 (PMC11293680; doi:10.1371/journal.pone.0307991)
Supplement: S12 Fig — The evolution of (a) αn and αp, (b) β, and (c) φ. (d) A positive correlation was observed between the evolved αp and the mean rate of the negative area of the two option distributions of the simulation. The almost same result was replicated as the asymmetric reinforcement learning model: that is, when agents experience at least one risk-seeking task, the value of αn decreased and on average the relationship αn > αp was observed. The inverse temperature (β) showed a larger variability as the number of risk-seeking task increased. The mean value of φ evolved from 1.19 to 1.83 except for the condition where agents only experienced the risk-aversion task (the mean value was 3.05). (PDF) [file pone.0307991.s016.pdf]

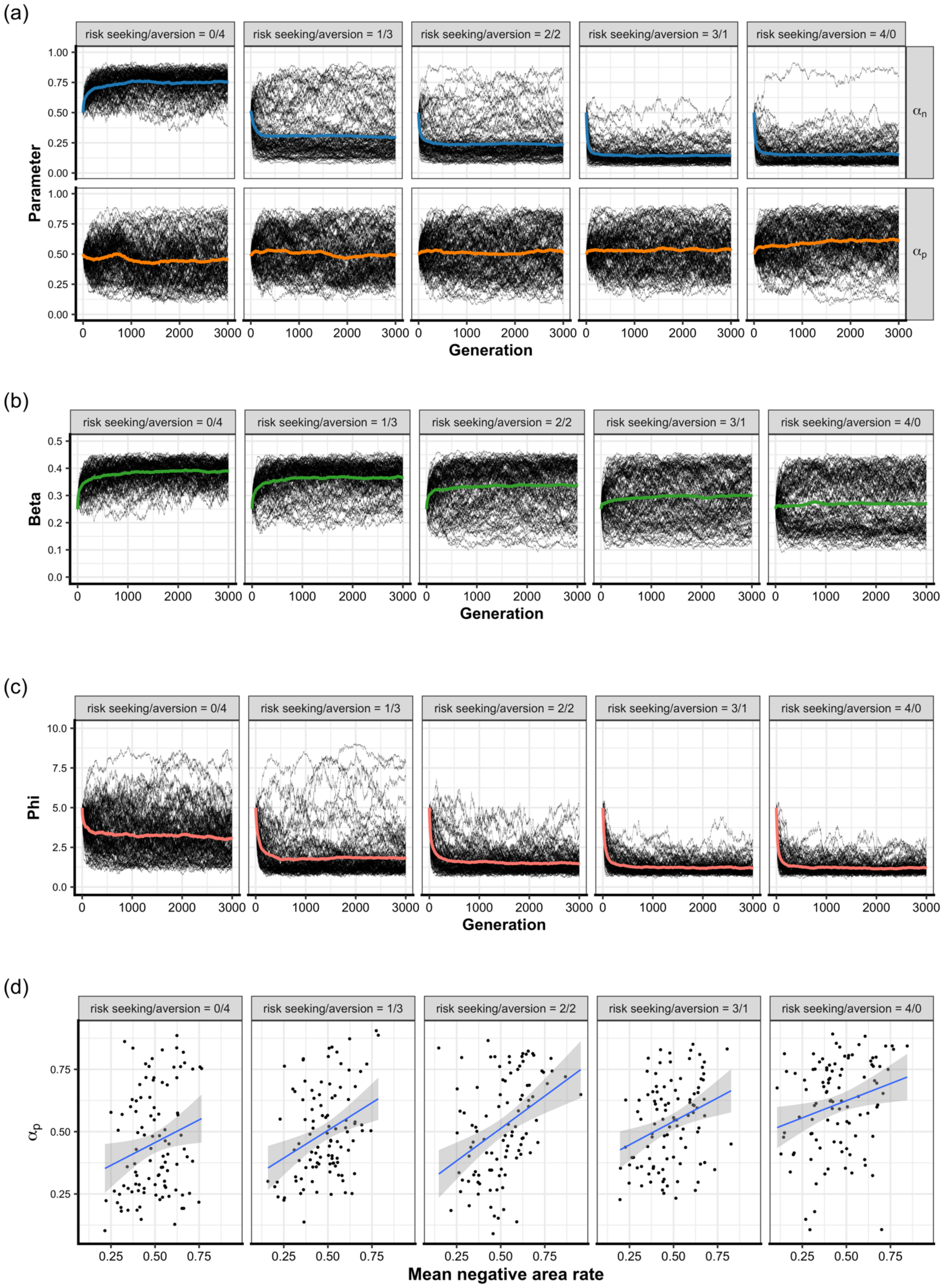
(a)
risk seeking/aversion = 0/4
risk seeking/aversion = 1/3
risk seeking/aversion = 2/2
risk seeking/aversion = 3/1
risk seeking/aversion = 4/0
Parameter
α_n
α_p
Generation
(b)
Beta
Generation
(c)
Phi
Generation
(d)
α_p
Mean negative area rate

S12 Fig. Evolutionary dynamics of parameters in the Hybrid model. The evolution of (a) α_n and α_p, (b) β, and (c) φ. (d) A positive correlation was observed between the evolved α_p and the mean rate of the negative area of the two option distributions of the simulation. The almost same result was replicated as the asymmetric reinforcement learning model: that is, when agents experience at least one risk-seeking task, the value of α_n decreased and on average the relationship $\alpha_n > \alpha_p$ was observed. The inverse temperature (β) showed a larger variability as the number of risk-seeking task increased. The mean value of φ evolved from 1.19 to 1.83 except for the condition where agents only experienced the risk-aversion task (the mean value was 3.05).
