# Supplementary material for: Risk preference as an outcome of evolutionarily adaptive learning mechanisms: An evolutionary simulation under diverse risky environments
Source: PLoS One. 2024 Aug 1;19(8):e0307991. doi: 10.1371/journal.pone.0307991 (PMC11293680; doi:10.1371/journal.pone.0307991)
Supplement: S13 Fig — (a) Comparison of the risk aversion rate between the first and final generations. Circles and vertical bars represent the mean and SD of risk aversion in the risk-seeking (dark orange) and risk-aversion tasks (light blue), respectively. (b) Comparison of average learning dynamics throughout trials between the first generation (top panel) and last generation (bottom panel). The solid line represents the mean risk aversion rate. Colored area shows ±1 SD. As with the asymmetric reinforcement learning model, the mean value of risk aversion improved in both risk-seeking and risk-averse tasks as a result of evolution. (PDF) [file pone.0307991.s017.pdf]

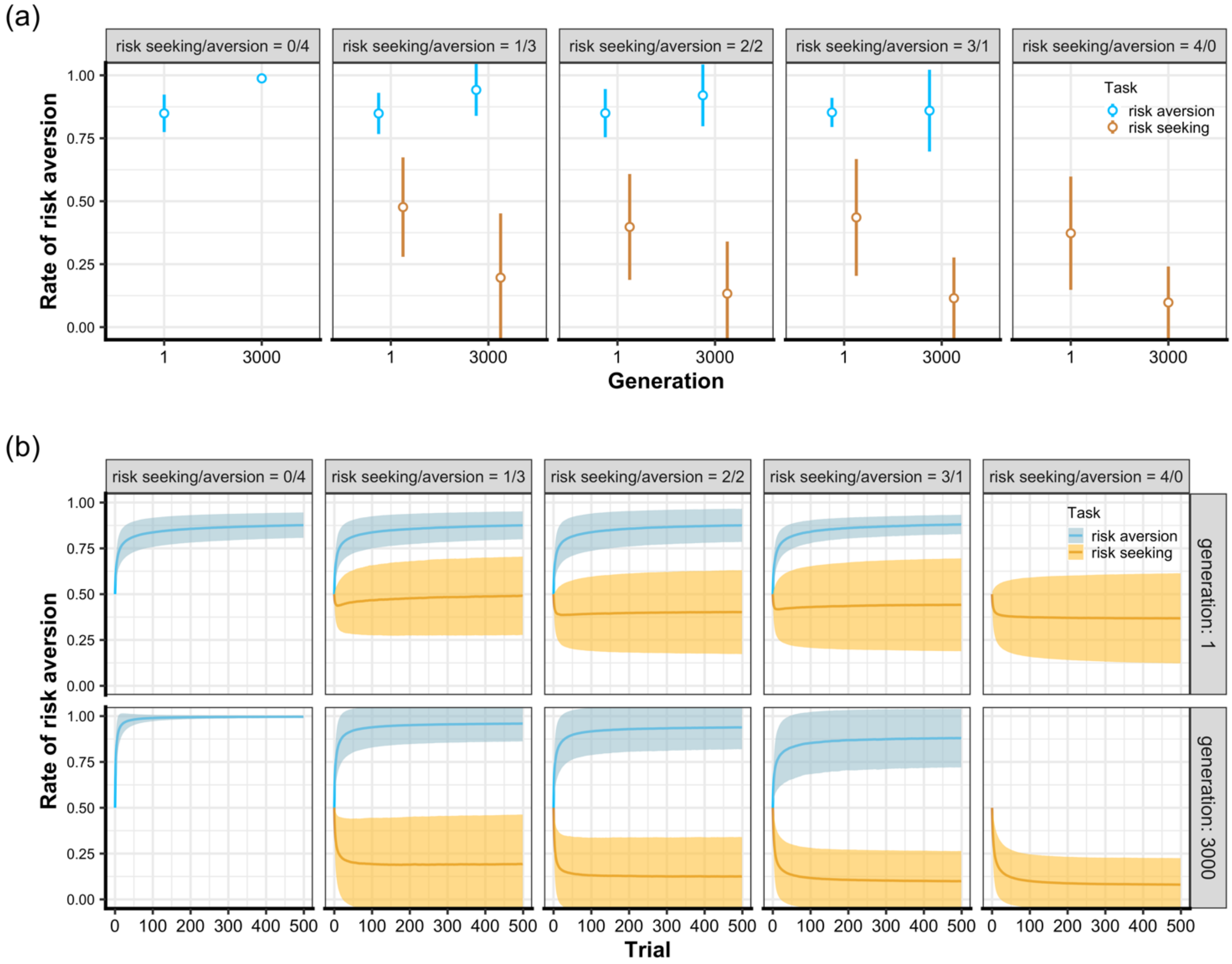

S13 Fig. Behavioral performance of the hybrid model. (a) Comparison of the risk aversion rate between the first and final generations. Circles and vertical bars represent the mean and SD of risk aversion in the risk-seeking (dark orange) and risk-aversion tasks (light blue), respectively. (b) Comparison of average learning dynamics throughout trials between the first generation (top panel) and last generation (bottom panel). The solid line represents the mean risk aversion rate. Colored area shows ±1 SD. As with the asymmetric reinforcement learning model, the mean value of risk aversion improved in both risk-seeking and risk-averse tasks as a result of evolution.
