# Supplementary material for: Risk preference as an outcome of evolutionarily adaptive learning mechanisms: An evolutionary simulation under diverse risky environments
Source: PLoS One. 2024 Aug 1;19(8):e0307991. doi: 10.1371/journal.pone.0307991 (PMC11293680; doi:10.1371/journal.pone.0307991)
Supplement: S14 Fig — The evolution of (a) α and (b) β. The averaged value of α decreased except the condition where agents only experience the risk-aversion task, which was similar to the evolution of αn within the asymmetric reinforcement learning model. The averaged β value increased regardless of the simulation condition. (PDF) [file pone.0307991.s018.pdf]

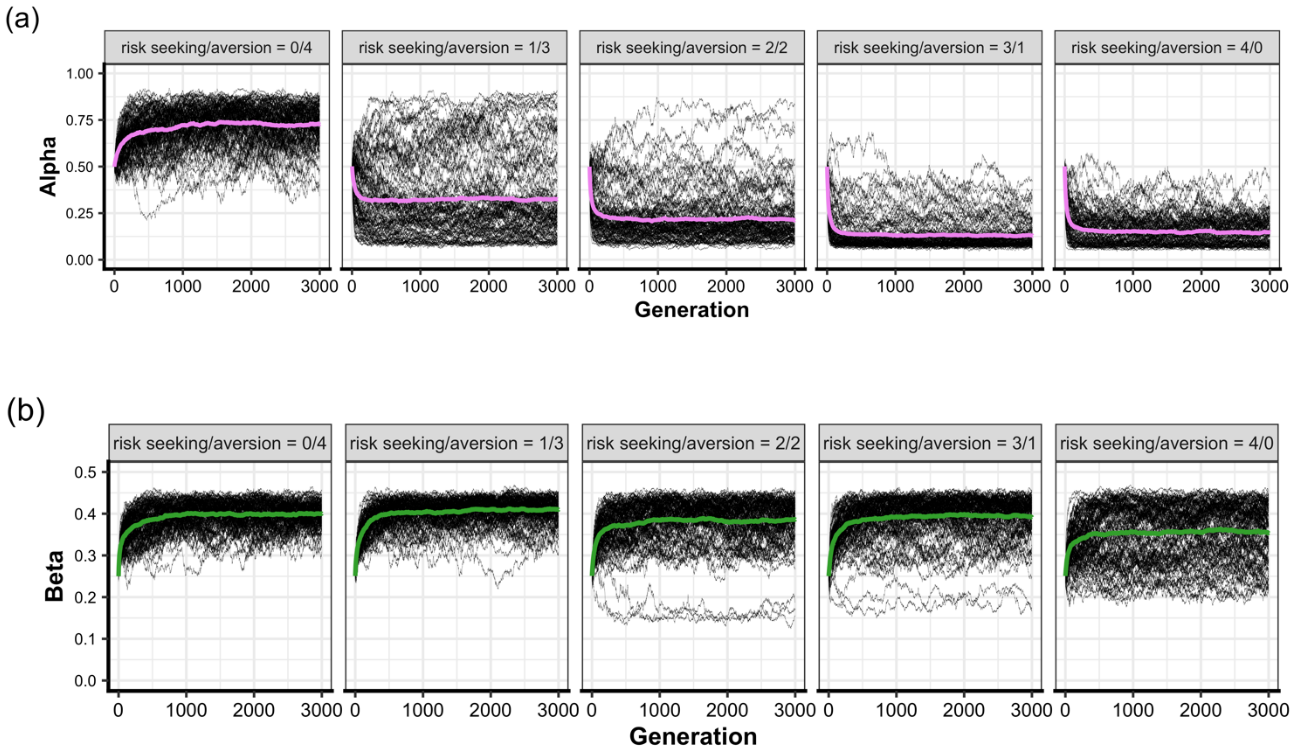

S14 Fig. Evolutionary dynamics of parameters of the single learning rate model. The evolution of (a) α and (b) β. The averaged value of α decreased except the condition where agents only experience the risk-aversion task, which was similar to the evolution of α_n within the asymmetric reinforcement learning model. The averaged β value increased regardless of the simulation condition.
