# Supplementary material for: Risk preference as an outcome of evolutionarily adaptive learning mechanisms: An evolutionary simulation under diverse risky environments
Source: PLoS One. 2024 Aug 1;19(8):e0307991. doi: 10.1371/journal.pone.0307991 (PMC11293680; doi:10.1371/journal.pone.0307991)
Supplement: S15 Fig — (a) Comparison of risk aversion rate between the first and final generation. Circles and vertical bars represent the mean and SD of risk aversion in the risk-seeking task (dark orange) and the risk-aversion tasks (light blue). (b) Comparison of the averaged learning dynamics through trials between the first generation (top panel) and last generation (bottom panel). The solid line represents the mean rate of risk aversion. The colored area shows ±1 SD. Although the single learning rate model has fewer parameters, it exhibits almost the same performance as the asymmetric reinforcement learning model. (PDF) [file pone.0307991.s019.pdf]

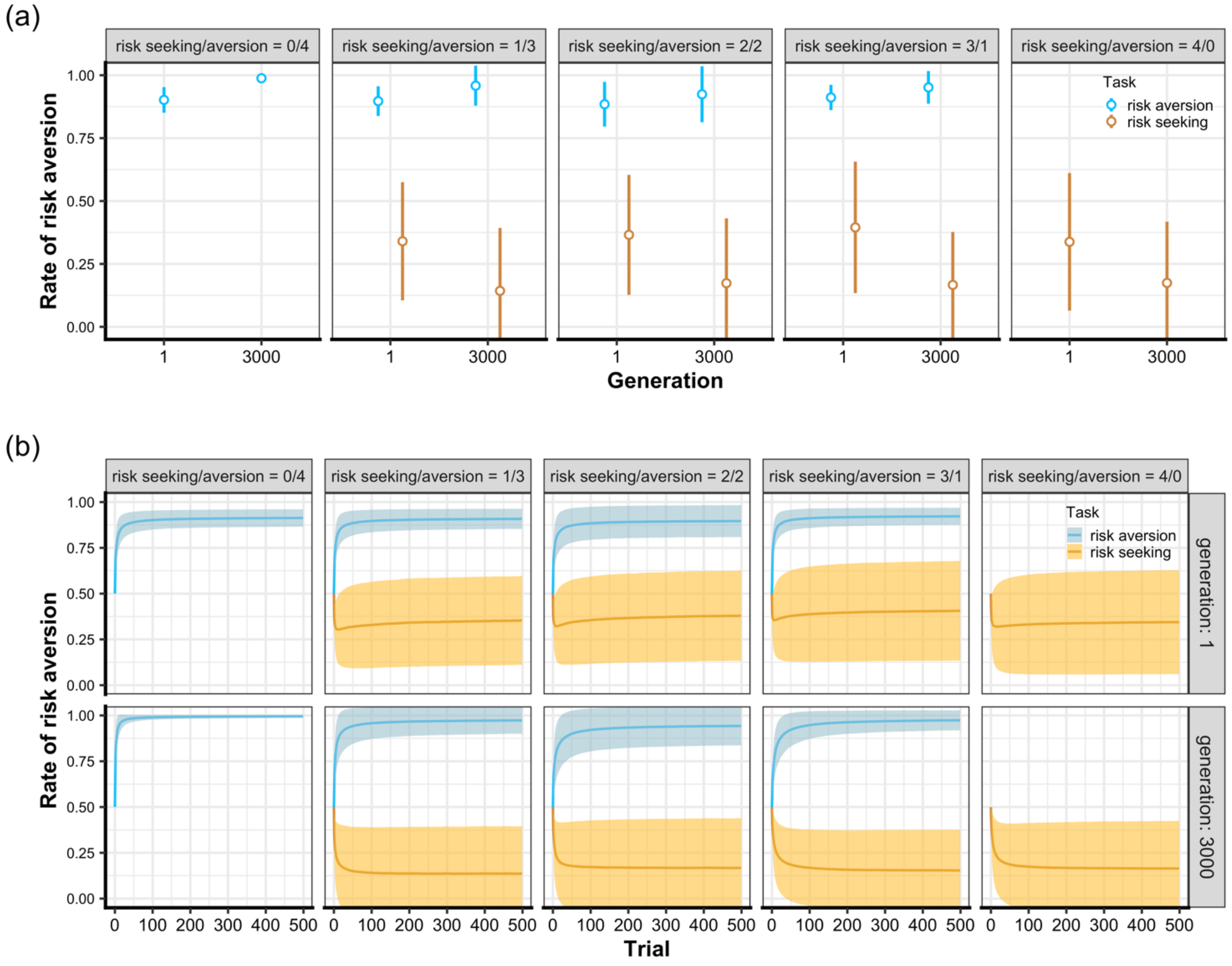

S15 Fig. Behavioral performance of the reinforcement learning model with the single learning rate. (a) Comparison of risk aversion rate between the first and final generation. Circles and vertical bars represent the mean and SD of risk aversion in the risk-seeking task (dark orange) and the risk-aversion tasks (light blue). (b) Comparison of the averaged learning dynamics through trials between the first generation (top panel) and last generation (bottom panel). The solid line represents the mean rate of risk aversion. The colored area shows ±1 SD. Although the single learning rate model has fewer parameters, it exhibits almost the same performance as the asymmetric reinforcement learning model.
