# Supplementary material for: Risk preference as an outcome of evolutionarily adaptive learning mechanisms: An evolutionary simulation under diverse risky environments
Source: PLoS One. 2024 Aug 1;19(8):e0307991. doi: 10.1371/journal.pone.0307991 (PMC11293680; doi:10.1371/journal.pone.0307991)
Supplement: S16 Fig — Rate of risk aversion was calculated from the last 100-trial choice of 500 trials. Histogram of the risk aversion rate of agents (a) in the first generation and (c) in the last generation. The difference of risk aversion rate between gain and loss domain (b) in the first generation and (d) in the last generation. If the difference is positive, the agent showed more risk aversion in the gain domain. Most agents in the first generation showed complete risk aversion regardless of the task domain and simulation condition, and the second-highest mode was observed at an intermediate level of risk aversion. As a result of this evolution, more agents tended to exhibit complete risk aversion when they experienced more risk-aversion tasks (e.g., the condition in which the number of risk-seeking tasks was zero or one). In the other evolutionary condition, the frequency of complete risk aversion decreased in the loss domain. Similar to the asymmetric reinforcement learning model, the evolved agents showed more risk aversion in the gain domain than in the loss domain (or more risk seeking in the loss domain). The rate of behavioral change between gain and loss was more than zero at 43.6%, 50.3%, and 52.5% when the number of risk-seeking tasks was one, two, and three in the evolutionary environment, respectively (see S3 Table). (PDF) [file pone.0307991.s020.pdf]

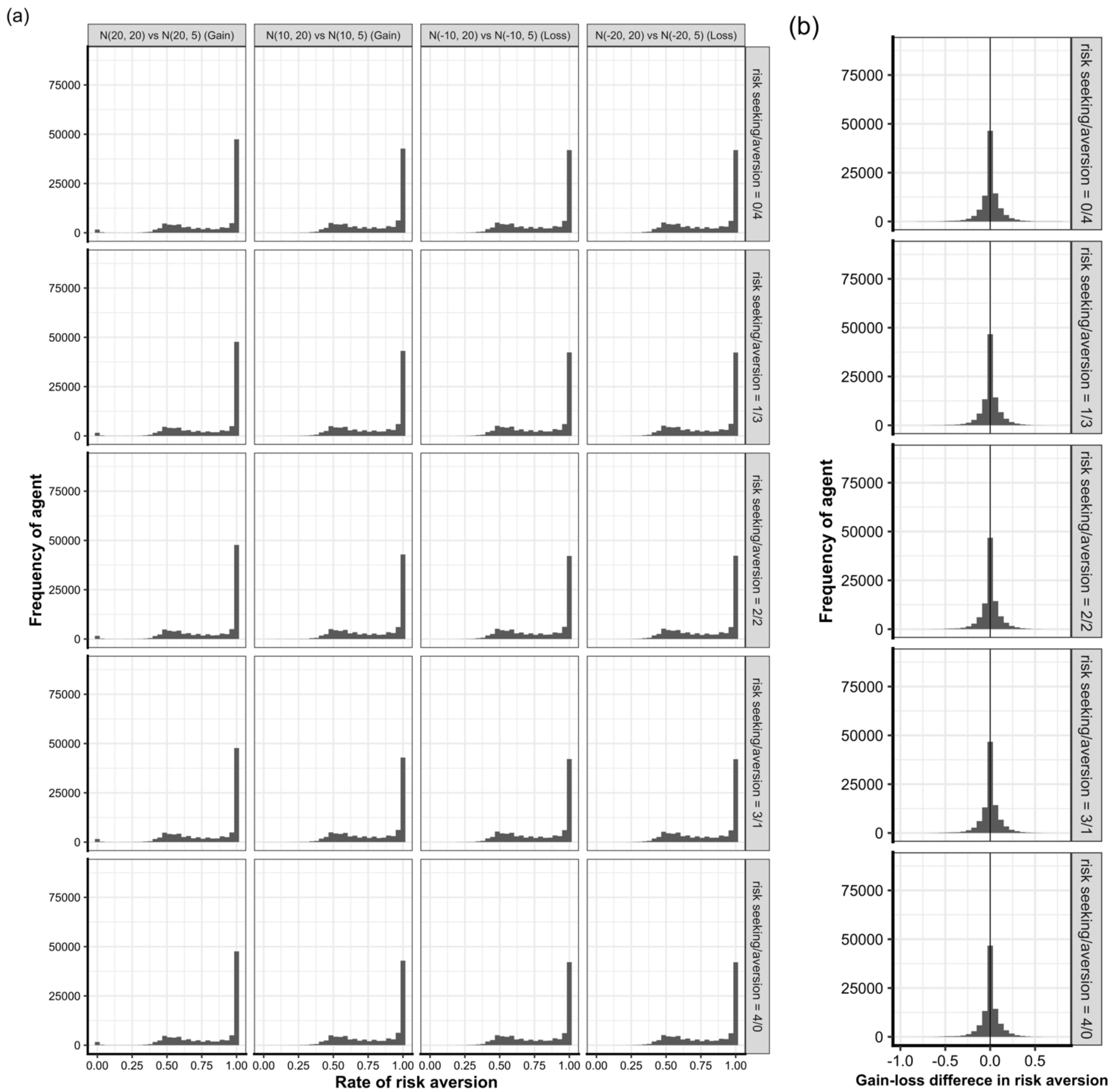

(a)
N(20, 20) vs N(20, 5) (Gain)
N(10, 20) vs N(10, 5) (Gain)
N(-10, 20) vs N(-10, 5) (Loss)
N(-20, 20) vs N(-20, 5) (Loss)
risk seeking/aversion = 0/4
risk seeking/aversion = 1/3
risk seeking/aversion = 2/2
risk seeking/aversion = 3/1
risk seeking/aversion = 4/0
Frequency of agent
Rate of risk aversion
0.00 0.25 0.50 0.75 1.00
0 25000 50000 75000
(b)
Frequency of agent
Gain-loss differece in risk aversion
-1.0 -0.5 0.0 0.5

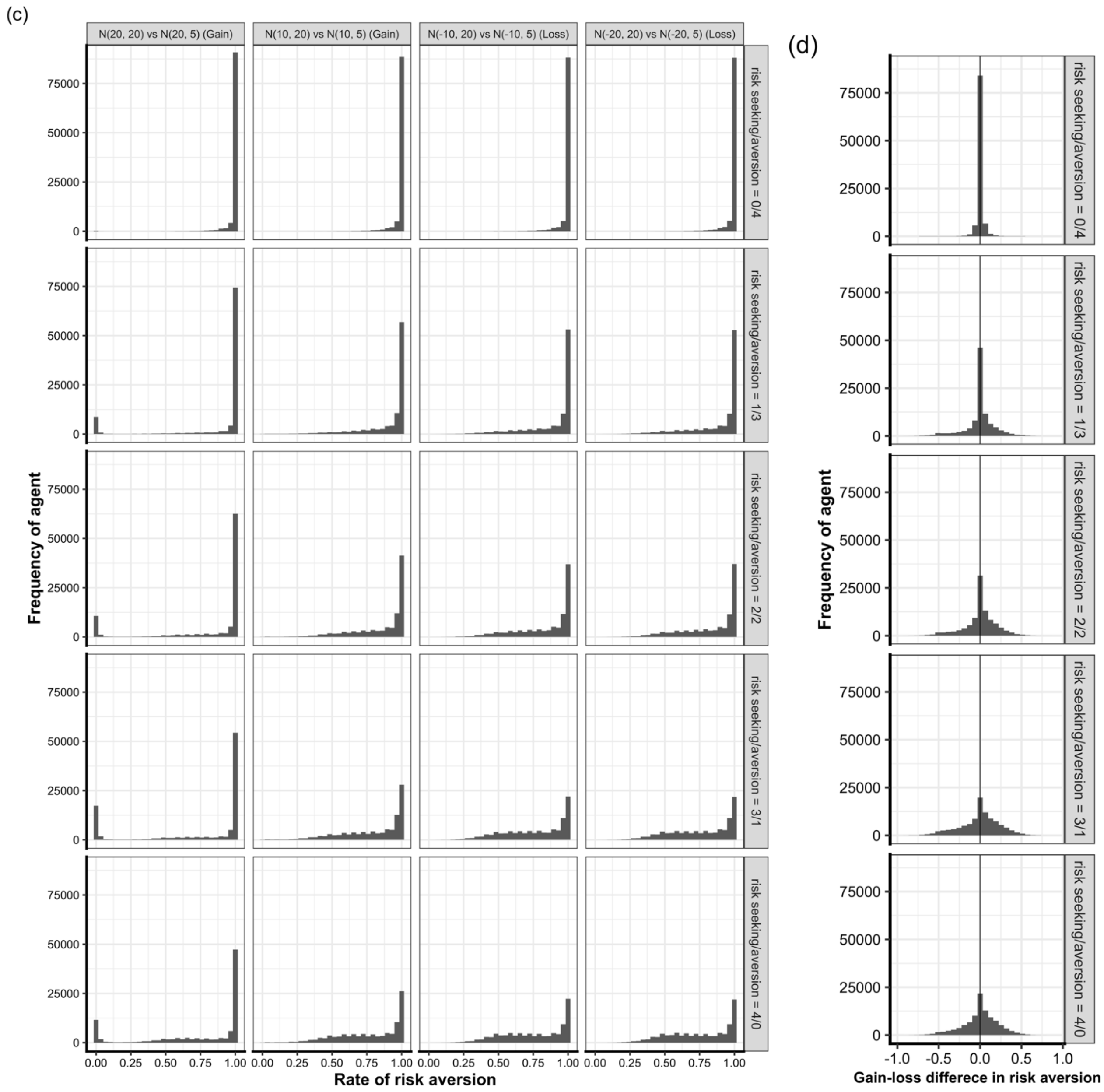

S16 Fig. Frequency of risk aversion rate of the single learning rate model in the tasks where the expected value of the two options was the same. Rate of risk aversion was calculated from the last 100-trial choice of 500 trials. Histogram of the risk aversion rate of agents (a) in the first generation and (c) in the last generation. The difference of risk aversion rate between gain and loss domain (b) in the first generation and (d) in the last generation. If the difference is positive, the agent showed more risk aversion in the gain domain. Most agents in the first generation showed complete risk aversion regardless of the task domain and simulation condition, and the

second-highest mode was observed at an intermediate level of risk aversion. As a result of this evolution, more agents tended to exhibit complete risk aversion when they experienced more risk-aversion tasks (e.g., the condition in which the number of risk-seeking tasks was zero or one). In the other evolutionary condition, the frequency of complete risk aversion decreased in the loss domain. Similar to the asymmetric reinforcement learning model, the evolved agents showed more risk aversion in the gain domain than in the loss domain (or more risk seeking in the loss domain). The rate of behavioral change between gain and loss was more than zero at 43.6%, 50.3%, and 52.5% when the number of risk-seeking tasks was one, two, and three in the evolutionary environment, respectively (see S3 Table).
