# Supplementary material for: Risk preference as an outcome of evolutionarily adaptive learning mechanisms: An evolutionary simulation under diverse risky environments
Source: PLoS One. 2024 Aug 1;19(8):e0307991. doi: 10.1371/journal.pone.0307991 (PMC11293680; doi:10.1371/journal.pone.0307991)
Supplement: S17 Fig — The horizontal axis represents the Niv index (αn − αp)/(αn + αp) calculated for each agent. The tasks are the same as Fig 1 in the main text. The column indicates the SD of the risky option (σ1). The row indicates the location of two distributions depicted by μ (risky vs safe option). Red and blue color correspond to the histogram of the first and last generation, respectively. In risk-aversion tasks, the histogram skewed to the positive value. In risk-seeking tasks, the histogram skewed to the negative value with three exceptions. (PDF) [file pone.0307991.s021.pdf]

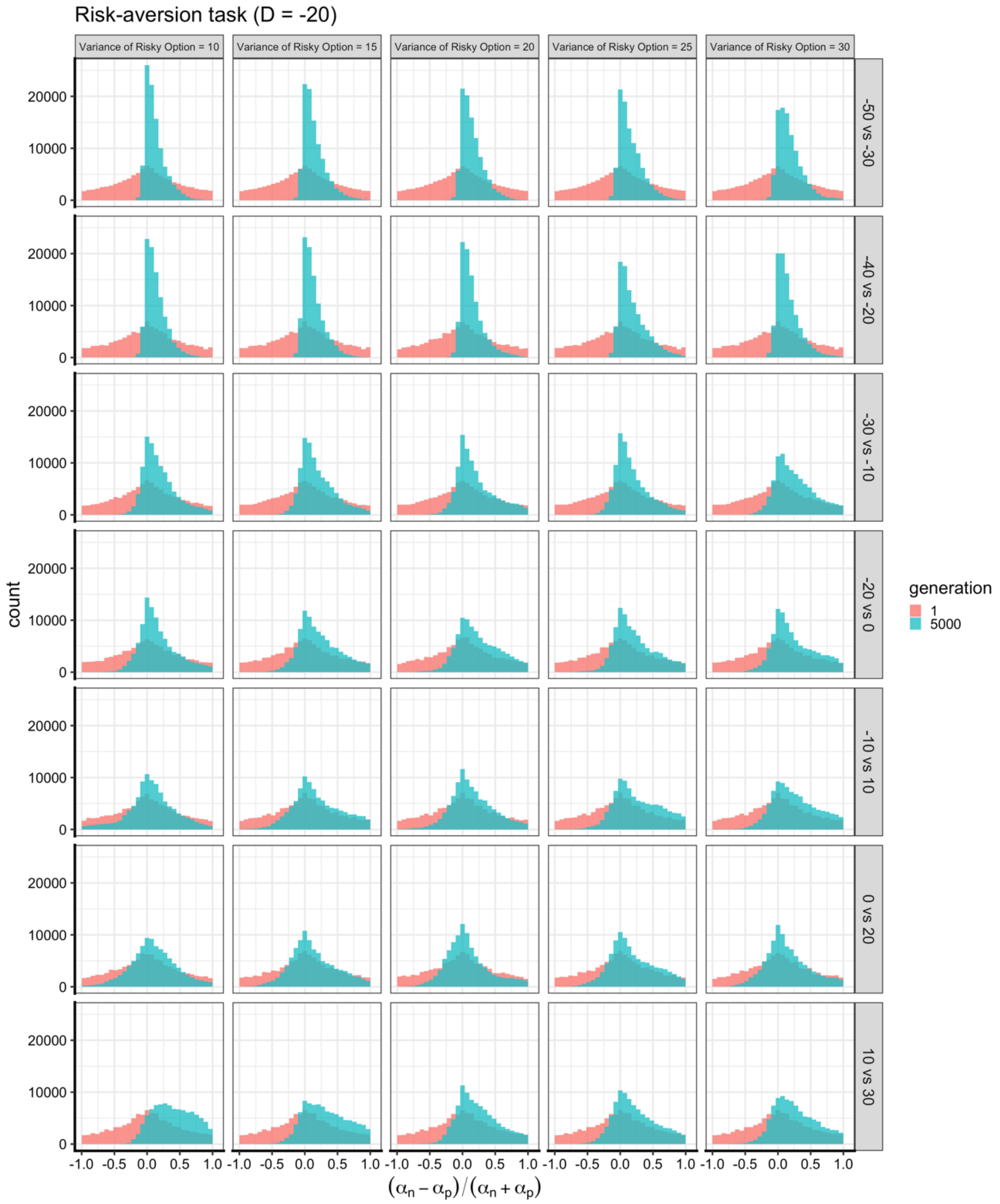

Risk-aversion task (D = -20)
Variance of Risky Option = 10
Variance of Risky Option = 15
Variance of Risky Option = 20
Variance of Risky Option = 25
Variance of Risky Option = 30
-50 vs -30
-40 vs -20
-30 vs -10
-20 vs 0
-10 vs 10
0 vs 20
10 vs 30
count
20000
10000
0
-1.0 -0.5 0.0 0.5 1.0
$(\alpha_n - \alpha_p)/(\alpha_n + \alpha_p)$
generation
1
5000

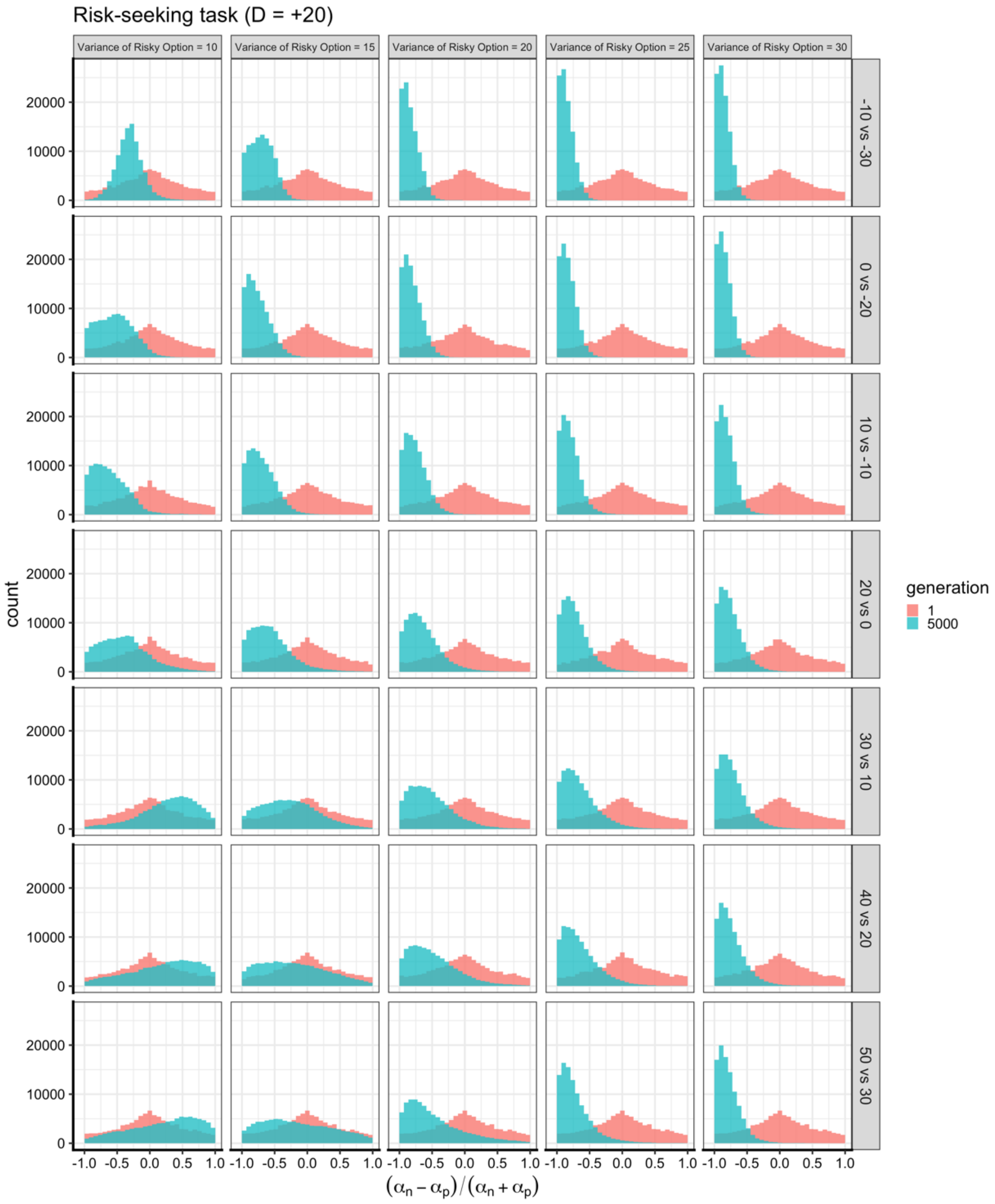

S17 Fig. Histogram of Niv index in the single-task simulation. The horizontal axis represents the Niv index $(\alpha_n - \alpha_p)/(\alpha_n + \alpha_p)$ calculated for each agent. The tasks are the same as Fig 1 in the main text. The column indicates the SD of the risky option (σ_1). The row indicates the location of two distributions depicted by μ (risky vs safe option). Red and blue color correspond to the histogram of the first and last generation, respectively. In risk-aversion tasks, the histogram skewed to the positive value. In risk-seeking tasks, the histogram skewed to the negative value with three exceptions.
