# Supplementary material for: Risk preference as an outcome of evolutionarily adaptive learning mechanisms: An evolutionary simulation under diverse risky environments
Source: PLoS One. 2024 Aug 1;19(8):e0307991. doi: 10.1371/journal.pone.0307991 (PMC11293680; doi:10.1371/journal.pone.0307991)
Supplement: S18 Fig — The horizontal axis represents the Niv index (αn − αp)/(αn + αp) calculated for each agent. Each column in the panel corresponds to a different condition. Red and blue color correspond to the histogram of the first and last generation, respectively. The histogram skewed to the negative value when agents experience a risk-seeking task. (PDF) [file pone.0307991.s022.pdf]

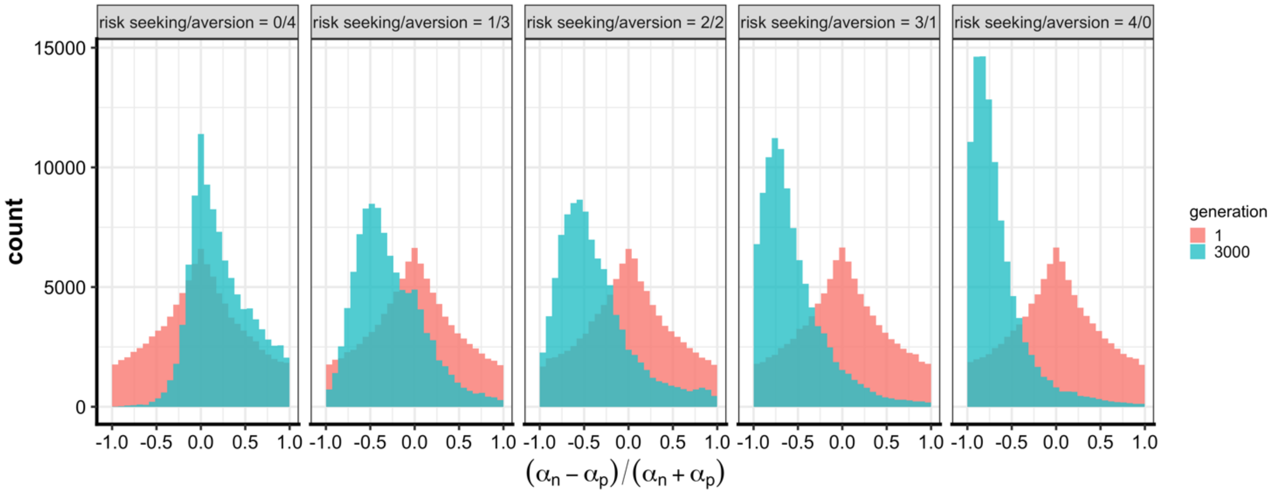

S18 Fig. Histogram of Niv index in the multiple-task simulation. The horizontal axis represents the Niv index $(\alpha_n - \alpha_p)/(\alpha_n + \alpha_p)$ calculated for each agent. Each column in the panel corresponds to a different condition. Red and blue color correspond to the histogram of the first and last generation, respectively. The histogram skewed to the negative value when agents experience a risk-seeking task.
