# Supplementary material for: Risk preference as an outcome of evolutionarily adaptive learning mechanisms: An evolutionary simulation under diverse risky environments
Source: PLoS One. 2024 Aug 1;19(8):e0307991. doi: 10.1371/journal.pone.0307991 (PMC11293680; doi:10.1371/journal.pone.0307991)
Supplement: S19 Fig — The scatter plot was created from the data of Fig 3 of the main text. Each point represents an agent. The dashed line is the fitted linear regression line. Pearson correlation coefficient is shown in the bottom. The positive correlation was observed between Niv index and the proportion of choosing the safe option. However, a large variance in the choice was found even though the value of Niv index was the same. (PDF) [file pone.0307991.s023.pdf]

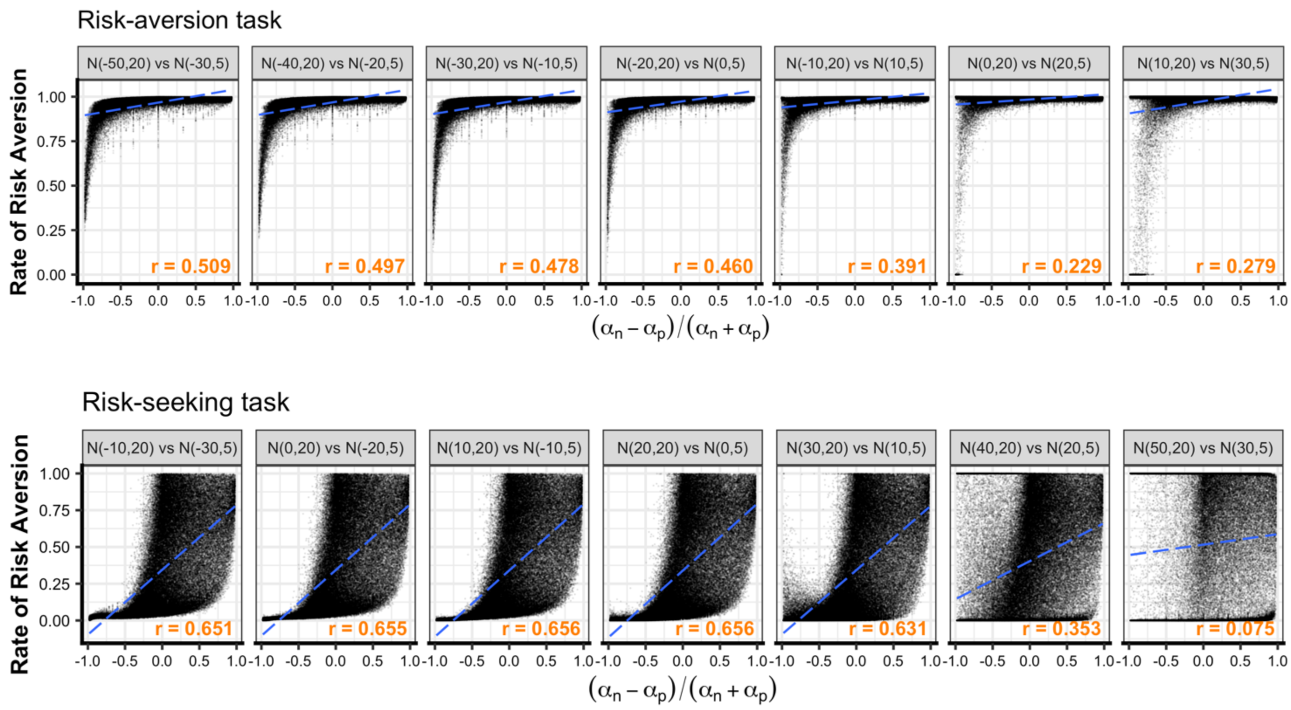

S19 Fig. Relationship between Niv index and risk aversion. The scatter plot was created from the data of Fig 3 of the main text. Each point represents an agent. The dashed line is the fitted linear regression line. Pearson correlation coefficient is shown in the bottom. The positive correlation was observed between Niv index and the proportion of choosing the safe option. However, a large variance in the choice was found even though the value of Niv index was the same.
