# Supplementary material for: Risk preference as an outcome of evolutionarily adaptive learning mechanisms: An evolutionary simulation under diverse risky environments
Source: PLoS One. 2024 Aug 1;19(8):e0307991. doi: 10.1371/journal.pone.0307991 (PMC11293680; doi:10.1371/journal.pone.0307991)
Supplement: S20 Fig — Cohen’s d for the evolved αp and αn was calculated for each task by d=Mαp-Mαn/σpooled and σpooled=SDαp2+SDαn2/2, where M and SD is the mean and standard deviation of an parameter in a task, respectively. The positive value of d indicates that the mean value of αp is larger than that of αn while the negative value of d indicates the opposite relationship. See S4 Table for the statistics of Cohen’s d and S5 Table for the detailed value of each task. (PDF) [file pone.0307991.s024.pdf]

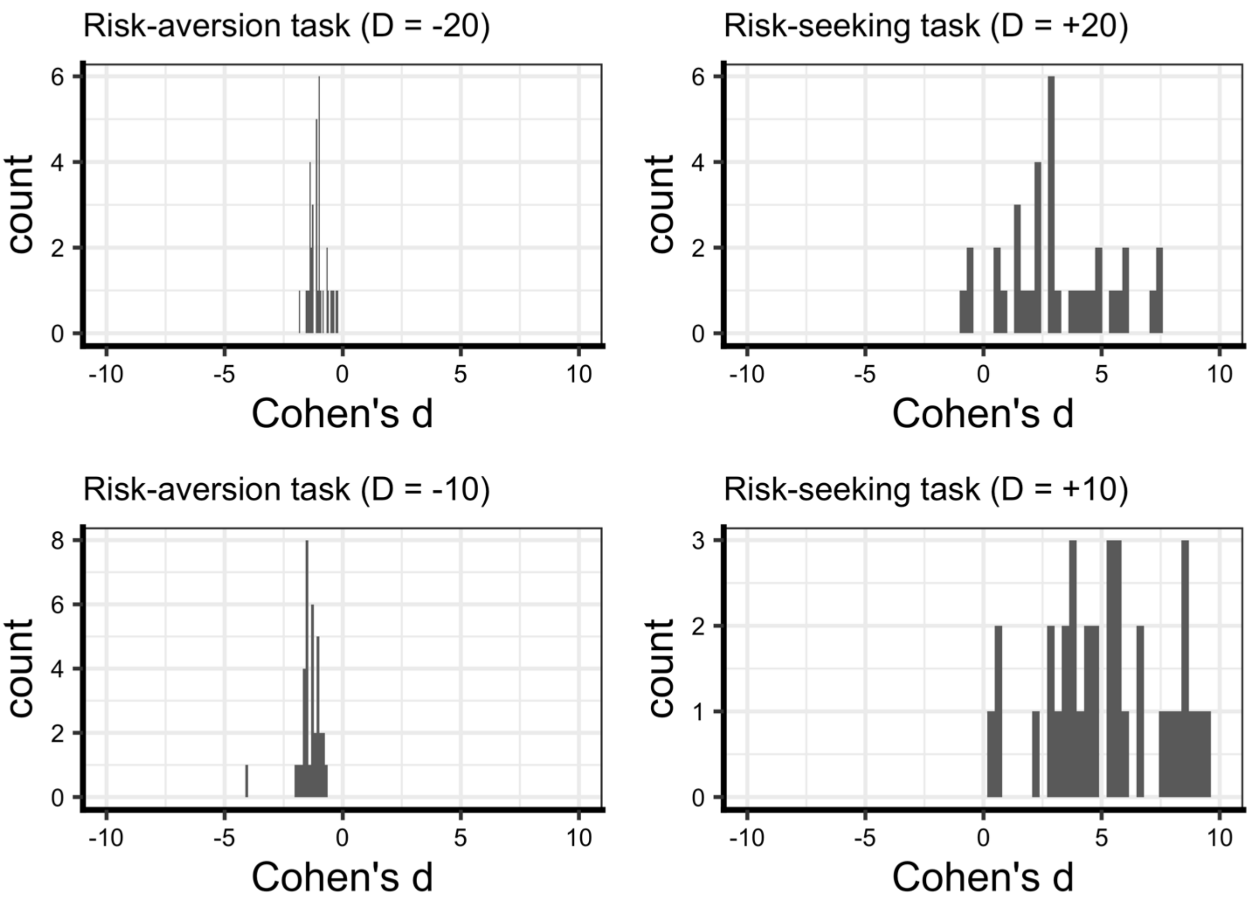

S20 Fig. Histogram of Cohen's *d* in the single-task simulation. Cohen's *d* for the evolved α_p and α_n was calculated for each task by $d = \left(M_{\alpha_p} - M_{\alpha_n}\right)/\sigma_{\text{pooled}}$ and $\sigma_{\text{pooled}} = \sqrt{\left(SD_{\alpha_p}^2 + SD_{\alpha_n}^2\right)/2}$, where M and SD is the mean and standard deviation of an parameter in a task, respectively. The positive value of *d* indicates that the mean value of α_p is larger than that of α_n while the negative value of *d* indicates the opposite relationship. See S4 Table for the statistics of Cohen's *d* and S5 Table for the detailed value of each task.
