# Supplementary material for: Risk preference as an outcome of evolutionarily adaptive learning mechanisms: An evolutionary simulation under diverse risky environments
Source: PLoS One. 2024 Aug 1;19(8):e0307991. doi: 10.1371/journal.pone.0307991 (PMC11293680; doi:10.1371/journal.pone.0307991)
Supplement: S21 Fig — Each column in the panel corresponds to a different condition. The Cohen’s d of the evolved αp and αn was calculated for each simulation. See the caption of S20 Fig for the calculation. The histogram shows that the most of the Cohen’s d is distributed in a positive range when agents experience a risk-seeking task. See S6 Table for the statistics of Cohen’s d and S7 Table for the summary of effect size. (PDF) [file pone.0307991.s025.pdf]

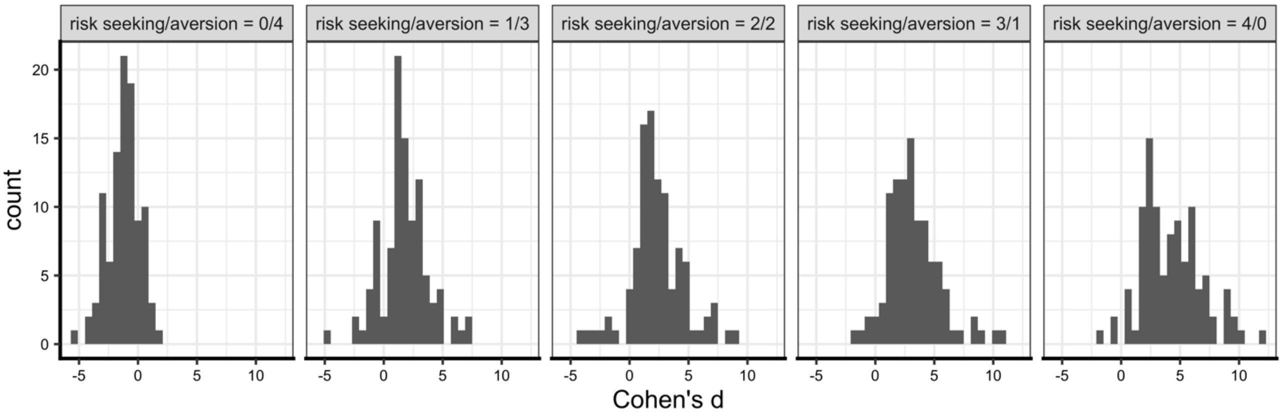

S21 Fig. Histogram of the Cohen's *d* in the multiple-task simulation. Each column in the panel corresponds to a different condition. The Cohen's *d* of the evolved α_p and α_n was calculated for each simulation. See the caption of S20 Fig for the calculation. The histogram shows that the most of the Cohen's *d* is distributed in a positive range when agents experience a risk-seeking task. See S6 Table for the statistics of Cohen's *d* and S7 Table for the summary of effect size.
