# Supplementary material for: Risk preference as an outcome of evolutionarily adaptive learning mechanisms: An evolutionary simulation under diverse risky environments
Source: PLoS One. 2024 Aug 1;19(8):e0307991. doi: 10.1371/journal.pone.0307991 (PMC11293680; doi:10.1371/journal.pone.0307991)
Supplement: S22 Fig — The ratio of standard deviation of the evolved αp to that of the evolved αn was calculated for each task. Most of the SD ratio was greater than one, suggesting that the SD of the evolved αp was sufficiently larger than that of the evolved αn. (PDF) [file pone.0307991.s026.pdf]

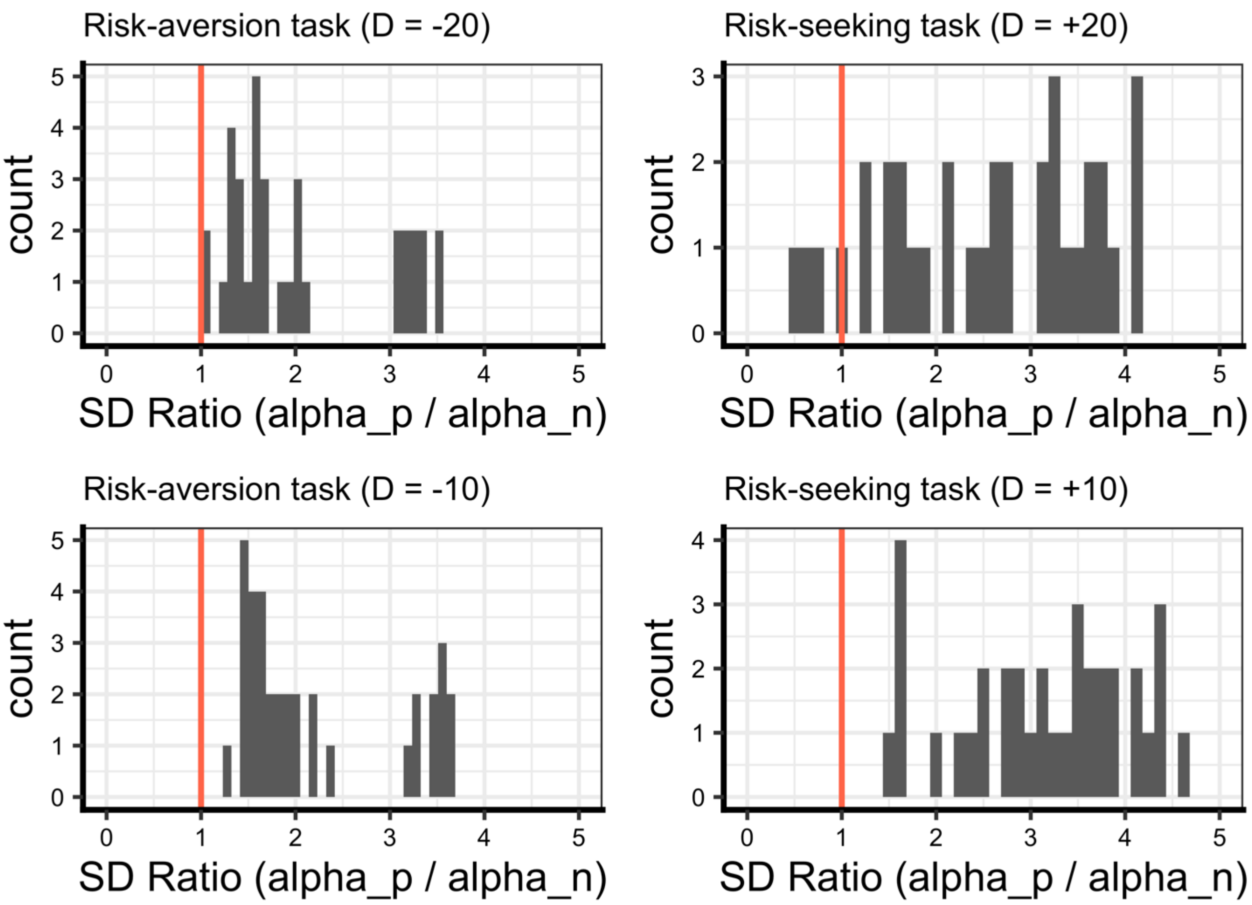

S22 Fig. Histogram of SD ratio in the single-task simulation. The ratio of standard deviation of the evolved α_p to that of the evolved α_n was calculated for each task. Most of the SD ratio was greater than one, suggesting that the SD of the evolved α_p was sufficiently larger than that of the evolved α_n.
