# Supplementary material for: Risk preference as an outcome of evolutionarily adaptive learning mechanisms: An evolutionary simulation under diverse risky environments
Source: PLoS One. 2024 Aug 1;19(8):e0307991. doi: 10.1371/journal.pone.0307991 (PMC11293680; doi:10.1371/journal.pone.0307991)
Supplement: S23 Fig — The heatmap of risk-aversion task of Fig 3. was magnified by dividing the y-axis (αn) by 0.1. The figure illustrates that risk-neutral or risk-seeking tendency can be found around the area where αn is close to zero, but not found in other area. (PDF) [file pone.0307991.s027.pdf]

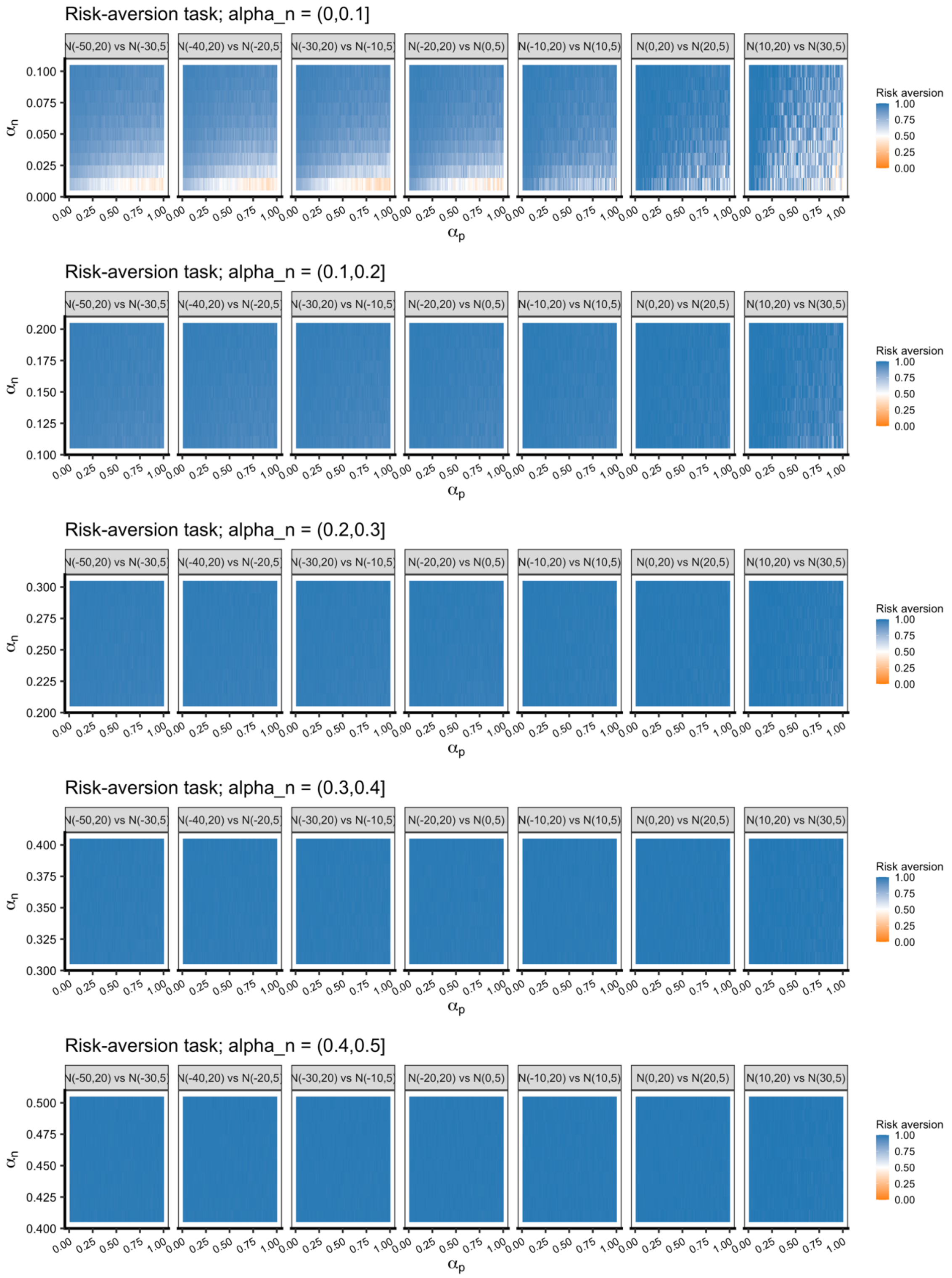

Risk-aversion task; alpha_n = (0,0.1]
Risk-aversion task; alpha_n = (0.1,0.2]
Risk-aversion task; alpha_n = (0.2,0.3]
Risk-aversion task; alpha_n = (0.3,0.4]
Risk-aversion task; alpha_n = (0.4,0.5]
N(-50,20) vs N(-30,5
N(-40,20) vs N(-20,5
N(-30,20) vs N(-10,5
N(-20,20) vs N(0,5)
N(-10,20) vs N(10,5)
N(0,20) vs N(20,5)
N(10,20) vs N(30,5)
α_n
α_p
Risk aversion
1.00
0.75
0.50
0.25
0.00

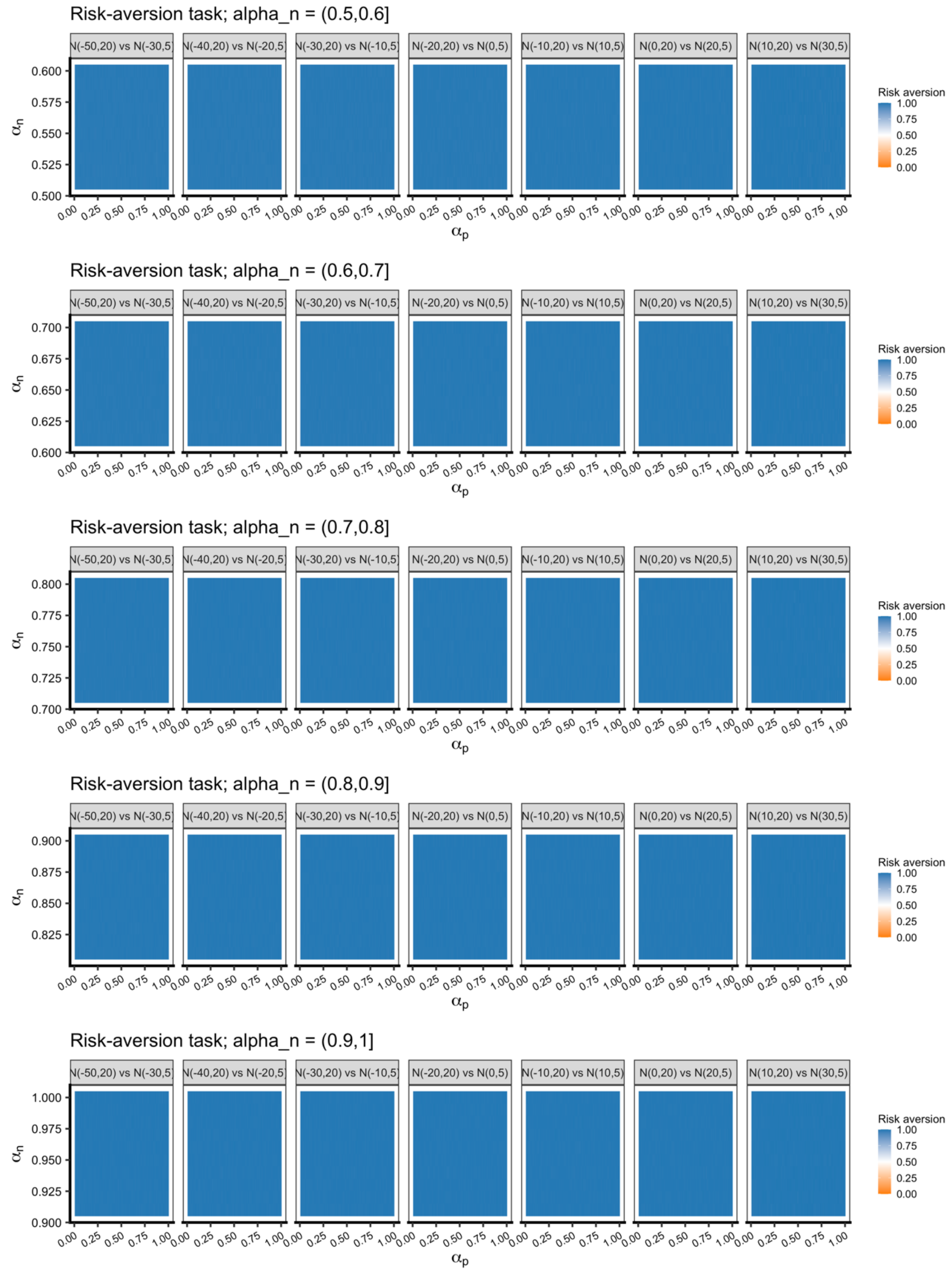

S23 Fig. Magnified heatmap of risk-aversion task. The heatmap of risk-aversion task of Fig 3. was magnified by dividing the y-axis (α_n) by 0.1. The figure illustrates that risk-neutral or risk-seeking tendency can be found around the area where α_n is close to zero, but not found in other area.
