# Supplementary material for: Risk preference as an outcome of evolutionarily adaptive learning mechanisms: An evolutionary simulation under diverse risky environments
Source: PLoS One. 2024 Aug 1;19(8):e0307991. doi: 10.1371/journal.pone.0307991 (PMC11293680; doi:10.1371/journal.pone.0307991)
Supplement: S1 Table — (PDF) [file pone.0307991.s028.pdf]

S1 Table. Task configuration in the multiple-task simulation

risk seeking/aversion = 0/4								
	task 1		task 2		task 3		task 4	
simulation	safe	risky	safe	risky	safe	risky	safe	risky
1	N(16.6, 5)	N(-11.4, 17)	N(-3.3, 5)	N(-24.6, 7.9)	N(23.9, 5)	N(6.1, 20.5)	N(5.5, 5)	N(-17.9, 15.3)
2	N(-4, 5)	N(-6.4, 22.2)	N(17.5, 5)	N(9.1, 6.8)	N(5.5, 5)	N(-17.9, 15.3)	N(4.5, 5)	N(-25.8, 15.3)
3	N(38.7, 5)	N(-4.1, 15.2)	N(4.4, 5)	N(-0.2, 9)	N(-3.3, 5)	N(-8.3, 23.1)	N(14.2, 5)	N(5.8, 7.7)
4	N(16.6, 5)	N(-11.4, 17)	N(14.2, 5)	N(5.8, 7.7)	N(13.2, 5)	N(4, 11.6)	N(15.5, 5)	N(-9.9, 25.9)
5	N(16.4, 5)	N(-9.6, 29)	N(16.6, 5)	N(-11.4, 17)	N(16.6, 5)	N(13.6, 10.7)	N(10.4, 5)	N(-2.8, 28)
6	N(-2.3, 5)	N(-15.2, 19)	N(10.2, 5)	N(-7.8, 23.9)	N(4.5, 5)	N(-25.8, 15.3)	N(19, 5)	N(-6, 27)
7	N(-3.3, 5)	N(-8.3, 23.1)	N(-6.8, 5)	N(-20.9, 12.5)	N(2.2, 5)	N(-2.4, 22.6)	N(24.4, 5)	N(-20.6, 17.2)
8	N(4.5, 5)	N(-25.8, 15.3)	N(0.7, 5)	N(-20.6, 21.5)	N(1.8, 5)	N(-27.2, 10.2)	N(16.6, 5)	N(-11.4, 17)
9	N(17.5, 5)	N(9.1, 6.8)	N(19, 5)	N(-6, 27)	N(-11.5, 5)	N(-19.6, 29.6)	N(16.4, 5)	N(-9.6, 29)
10	N(10.7, 5)	N(-24.3, 24.7)	N(-3.5, 5)	N(-14.8, 26.7)	N(38.7, 5)	N(-4.1, 15.2)	N(17.5, 5)	N(9.1, 6.8)
11	N(21.7, 5)	N(-16.2, 19.6)	N(13.2, 5)	N(4, 11.6)	N(4.5, 5)	N(-25.8, 15.3)	N(47.4, 5)	N(-10.4, 17.7)
12	N(-3.5, 5)	N(-14.8, 26.7)	N(17.1, 5)	N(-22.1, 27.8)	N(24.4, 5)	N(-20.6, 17.2)	N(21.7, 5)	N(-16.2, 19.6)
13	N(-4, 5)	N(-6.4, 22.2)	N(10.4, 5)	N(-6.7, 12.4)	N(10.9, 5)	N(-9.4, 11.8)	N(-25.1, 5)	N(-41.6, 11.2)
14	N(-0.5, 5)	N(-13.7, 21.9)	N(-22.1, 5)	N(-36.1, 5.7)	N(13.2, 5)	N(4, 11.6)	N(15.5, 5)	N(-9.9, 25.9)
15	N(19, 5)	N(-6, 27)	N(0.7, 5)	N(-20.6, 21.5)	N(-3.3, 5)	N(-24.6, 7.9)	N(2.8, 5)	N(-16.3, 17.6)
16	N(23.9, 5)	N(6.1, 20.5)	N(22.1, 5)	N(15.5, 18.5)	N(16.1, 5)	N(-18, 27.7)	N(-18.6, 5)	N(-19.6, 23.9)
17	N(5.7, 5)	N(2.2, 13.4)	N(-4, 5)	N(-6.4, 22.2)	N(-2.3, 5)	N(-15.2, 19)	N(5.5, 5)	N(-17.9, 15.3)
18	N(18.5, 5)	N(14.2, 28.8)	N(16.1, 5)	N(-18, 27.7)	N(1.8, 5)	N(-27.2, 10.2)	N(2.2, 5)	N(-2.4, 22.6)
19	N(47.4, 5)	N(-10.4, 17.7)	N(0.7, 5)	N(-20.6, 21.5)	N(21.7, 5)	N(-16.2, 19.6)	N(0.5, 5)	N(-9.3, 20.6)
20	N(10.4, 5)	N(-6.7, 12.4)	N(16.6, 5)	N(-11.4, 17)	N(47.4, 5)	N(-10.4, 17.7)	N(23.9, 5)	N(6.1, 20.5)
21	N(-18.6, 5)	N(-19.6, 23.9)	N(16.4, 5)	N(-9.6, 29)	N(-2.3, 5)	N(-15.2, 19)	N(2.2, 5)	N(-2.4, 22.6)
22	N(-3.3, 5)	N(-8.3, 23.1)	N(24.4, 5)	N(-20.6, 17.2)	N(18.5, 5)	N(14.2, 28.8)	N(-2.3, 5)	N(-15.2, 19)
23	N(-4.2, 5)	N(-8.8, 11.3)	N(-18.6, 5)	N(-19.6, 23.9)	N(23.9, 5)	N(6.1, 20.5)	N(14.8, 5)	N(2.4, 21.3)
24	N(38.7, 5)	N(-4.1, 15.2)	N(17.5, 5)	N(9.1, 6.8)	N(10.7, 5)	N(-24.3, 24.7)	N(-2.3, 5)	N(-15.2, 19)
25	N(10.9, 5)	N(-9.4, 11.8)	N(24.4, 5)	N(-20.6, 17.2)	N(-22.1, 5)	N(-36.1, 5.7)	N(-2.3, 5)	N(-15.2, 19)
26	N(10.4, 5)	N(-2.8, 28)	N(1.8, 5)	N(-27.2, 10.2)	N(-3.3, 5)	N(-24.6, 7.9)	N(-11.5, 5)	N(-19.6, 29.6)
27	N(16.1, 5)	N(9.5, 23.4)	N(14.2, 5)	N(5.8, 7.7)	N(13.2, 5)	N(4, 11.6)	N(19, 5)	N(-6, 27)
28	N(-4.2, 5)	N(-8.8, 11.3)	N(16.1, 5)	N(-18, 27.7)	N(19, 5)	N(-6, 27)	N(-0.5, 5)	N(-13.7, 21.9)
29	N(-4.2, 5)	N(-8.8, 11.3)	N(24.4, 5)	N(-20.6, 17.2)	N(-4, 5)	N(-6.4, 22.2)	N(21.7, 5)	N(-16.2, 19.6)
30	N(22.1, 5)	N(15.5, 18.5)	N(10.2, 5)	N(-7.8, 23.9)	N(-18.6, 5)	N(-19.6, 23.9)	N(13.2, 5)	N(4, 11.6)
31	N(38.7, 5)	N(-4.1, 15.2)	N(33.1, 5)	N(-12.6, 15)	N(14.8, 5)	N(2.4, 21.3)	N(-0.5, 5)	N(-13.7, 21.9)
32	N(47.4, 5)	N(-10.4, 17.7)	N(14.8, 5)	N(2.4, 21.3)	N(10.7, 5)	N(-24.3, 24.7)	N(13.2, 5)	N(4, 11.6)
33	N(-14.8, 5)	N(-18.9, 27.9)	N(-0.5, 5)	N(-13.7, 21.9)	N(-3.5, 5)	N(-14.8, 26.7)	N(14.9, 5)	N(11.9, 14.9)
34	N(2.2, 5)	N(-2.4, 22.6)	N(-25.1, 5)	N(-41.6, 11.2)	N(5.7, 5)	N(2.2, 13.4)	N(14.9, 5)	N(11.9, 14.9)
35	N(-3.3, 5)	N(-8.3, 23.1)	N(5.5, 5)	N(-17.9, 15.3)	N(-14.8, 5)	N(-18.9, 27.9)	N(-6.8, 5)	N(-20.9, 12.5)
36	N(13.2, 5)	N(4, 11.6)	N(1.8, 5)	N(-27.2, 10.2)	N(-6.8, 5)	N(-20.9, 12.5)	N(-4, 5)	N(-6.4, 22.2)
37	N(14.9, 5)	N(11.9, 14.9)	N(-3.3, 5)	N(-8.3, 23.1)	N(24.4, 5)	N(-20.6, 17.2)	N(-3.5, 5)	N(-14.8, 26.7)
38	N(10.4, 5)	N(-6.7, 12.4)	N(-14.8, 5)	N(-18.9, 27.9)	N(-0.5, 5)	N(-13.7, 21.9)	N(14.8, 5)	N(2.4, 21.3)
39	N(22.1, 5)	N(15.5, 18.5)	N(-0.5, 5)	N(-13.7, 21.9)	N(47.4, 5)	N(-10.4, 17.7)	N(1.8, 5)	N(-27.2, 10.2)
40	N(14.9, 5)	N(11.9, 14.9)	N(-3.3, 5)	N(-24.6, 7.9)	N(-3.5, 5)	N(-14.8, 26.7)	N(24.4, 5)	N(-20.6, 17.2)
41	N(16.6, 5)	N(-11.4, 17)	N(-0.5, 5)	N(-13.7, 21.9)	N(-18.6, 5)	N(-19.6, 23.9)	N(-4, 5)	N(-6.4, 22.2)
42	N(-18.6, 5)	N(-19.6, 23.9)	N(10.2, 5)	N(-7.8, 23.9)	N(22.1, 5)	N(15.5, 18.5)	N(10.9, 5)	N(-9.4, 11.8)
43	N(-14.8, 5)	N(-18.9, 27.9)	N(16.4, 5)	N(-9.6, 29)	N(-2.3, 5)	N(-15.2, 19)	N(-6.8, 5)	N(-20.9, 12.5)
44	N(16.1, 5)	N(9.5, 23.4)	N(2.8, 5)	N(-16.3, 17.6)	N(0.5, 5)	N(-9.3, 20.6)	N(4.5, 5)	N(-25.8, 15.3)
45	N(14.2, 5)	N(5.8, 7.7)	N(-3.3, 5)	N(-8.3, 23.1)	N(16.1, 5)	N(9.5, 23.4)	N(-22.1, 5)	N(-36.1, 5.7)
46	N(10.9, 5)	N(-9.4, 11.8)	N(10.4, 5)	N(-6.7, 12.4)	N(16.4, 5)	N(-9.6, 29)	N(4.4, 5)	N(-0.2, 9)
47	N(10.7, 5)	N(-24.3, 24.7)	N(16.4, 5)	N(-9.6, 29)	N(24.4, 5)	N(-20.6, 17.2)	N(14.8, 5)	N(2.4, 21.3)
48	N(10.9, 5)	N(-9.4, 11.8)	N(1.8, 5)	N(-27.2, 10.2)	N(-4.2, 5)	N(-8.8, 11.3)	N(-2.3, 5)	N(-15.2, 19)
49	N(5.7, 5)	N(2.2, 13.4)	N(-3.3, 5)	N(-24.6, 7.9)	N(-4.2, 5)	N(-8.8, 11.3)	N(-20.1, 5)	N(-22.4, 15.4)
50	N(-18.6, 5)	N(-19.6, 23.9)	N(-0.5, 5)	N(-13.7, 21.9)	N(5.7, 5)	N(2.2, 13.4)	N(2.8, 5)	N(-16.3, 17.6)

51	N(-2.3, 5)	N(-15.2, 19)	N(10.2, 5)	N(-7.8, 23.9)	N(5.7, 5)	N(2.2, 13.4)	N(2.2, 5)	N(-2.4, 22.6)
52	N(-6.8, 5)	N(-20.9, 12.5)	N(-4, 5)	N(-6.4, 22.2)	N(2.8, 5)	N(-16.3, 17.6)	N(15.5, 5)	N(-9.9, 25.9)
53	N(-3.3, 5)	N(-8.3, 23.1)	N(5.5, 5)	N(-17.9, 15.3)	N(16.1, 5)	N(-18, 27.7)	N(16.1, 5)	N(9.5, 23.4)
54	N(14.2, 5)	N(5.8, 7.7)	N(16.1, 5)	N(9.5, 23.4)	N(-4, 5)	N(-6.4, 22.2)	N(17.5, 5)	N(9.1, 6.8)
55	N(15.5, 5)	N(-9.9, 25.9)	N(33.1, 5)	N(-12.6, 15)	N(22.1, 5)	N(15.5, 18.5)	N(-22.1, 5)	N(-36.1, 5.7)
56	N(22.1, 5)	N(15.5, 18.5)	N(-3.3, 5)	N(-8.3, 23.1)	N(16.6, 5)	N(-11.4, 17)	N(-14.8, 5)	N(-18.9, 27.9)
57	N(-25.1, 5)	N(-41.6, 11.2)	N(4.5, 5)	N(-25.8, 15.3)	N(10.4, 5)	N(-2.8, 28)	N(5.7, 5)	N(2.2, 13.4)
58	N(14.2, 5)	N(5.8, 7.7)	N(2.8, 5)	N(-16.3, 17.6)	N(16.1, 5)	N(-18, 27.7)	N(16.6, 5)	N(-11.4, 17)
59	N(33.1, 5)	N(-12.6, 15)	N(-3.3, 5)	N(-24.6, 7.9)	N(10.4, 5)	N(-6.7, 12.4)	N(2.8, 5)	N(-16.3, 17.6)
60	N(14.8, 5)	N(2.4, 21.3)	N(19, 5)	N(-6, 27)	N(4.5, 5)	N(-25.8, 15.3)	N(-18.6, 5)	N(-19.6, 23.9)
61	N(16.4, 5)	N(-9.6, 29)	N(10.4, 5)	N(-2.8, 28)	N(1.8, 5)	N(-27.2, 10.2)	N(4.5, 5)	N(-25.8, 15.3)
62	N(16.4, 5)	N(-9.6, 29)	N(47.4, 5)	N(-10.4, 17.7)	N(2.8, 5)	N(-16.3, 17.6)	N(17.5, 5)	N(9.1, 6.8)
63	N(4.5, 5)	N(-25.8, 15.3)	N(14.9, 5)	N(11.9, 14.9)	N(14.8, 5)	N(2.4, 21.3)	N(0.5, 5)	N(-9.3, 20.6)
64	N(-14.8, 5)	N(-18.9, 27.9)	N(38.7, 5)	N(-4.1, 15.2)	N(16.1, 5)	N(9.5, 23.4)	N(17.1, 5)	N(-22.1, 27.8)
65	N(-6.8, 5)	N(-20.9, 12.5)	N(33.1, 5)	N(-12.6, 15)	N(5.5, 5)	N(-17.9, 15.3)	N(14.8, 5)	N(2.4, 21.3)
66	N(-0.5, 5)	N(-13.7, 21.9)	N(-4.4, 5)	N(-11.1, 11.3)	N(-3.5, 5)	N(-14.8, 26.7)	N(47.4, 5)	N(-10.4, 17.7)
67	N(22.1, 5)	N(15.5, 18.5)	N(-25.1, 5)	N(-41.6, 11.2)	N(4.4, 5)	N(-0.2, 9)	N(38.7, 5)	N(-4.1, 15.2)
68	N(14.9, 5)	N(11.9, 14.9)	N(1.8, 5)	N(-27.2, 10.2)	N(16.6, 5)	N(13.6, 10.7)	N(-20.1, 5)	N(-22.4, 15.4)
69	N(-4, 5)	N(-6.4, 22.2)	N(21.7, 5)	N(-16.2, 19.6)	N(-18.6, 5)	N(-19.6, 23.9)	N(38.7, 5)	N(-4.1, 15.2)
70	N(14.2, 5)	N(5.8, 7.7)	N(-2.3, 5)	N(-15.2, 19)	N(15.5, 5)	N(-9.9, 25.9)	N(-14.8, 5)	N(-18.9, 27.9)
71	N(14.9, 5)	N(11.9, 14.9)	N(2.2, 5)	N(-2.4, 22.6)	N(-14.8, 5)	N(-18.9, 27.9)	N(-4.4, 5)	N(-11.1, 11.3)
72	N(14.9, 5)	N(11.9, 14.9)	N(17.1, 5)	N(-22.1, 27.8)	N(-14.8, 5)	N(-18.9, 27.9)	N(15.5, 5)	N(-9.9, 25.9)
73	N(-18.6, 5)	N(-19.6, 23.9)	N(16.6, 5)	N(13.6, 10.7)	N(16.1, 5)	N(9.5, 23.4)	N(33.1, 5)	N(-12.6, 15)
74	N(10.9, 5)	N(-9.4, 11.8)	N(10.4, 5)	N(-2.8, 28)	N(-6.8, 5)	N(-20.9, 12.5)	N(-2.3, 5)	N(-15.2, 19)
75	N(10.7, 5)	N(-24.3, 24.7)	N(13.2, 5)	N(4, 11.6)	N(10.4, 5)	N(-6.7, 12.4)	N(22.1, 5)	N(15.5, 18.5)
76	N(5.7, 5)	N(2.2, 13.4)	N(17.5, 5)	N(9.1, 6.8)	N(10.4, 5)	N(-6.7, 12.4)	N(-4.4, 5)	N(-11.1, 11.3)
77	N(10.4, 5)	N(-6.7, 12.4)	N(17.5, 5)	N(9.1, 6.8)	N(-3.3, 5)	N(-8.3, 23.1)	N(16.1, 5)	N(9.5, 23.4)
78	N(10.4, 5)	N(-6.7, 12.4)	N(-22.1, 5)	N(-36.1, 5.7)	N(17.5, 5)	N(9.1, 6.8)	N(-14.8, 5)	N(-18.9, 27.9)
79	N(-0.5, 5)	N(-13.7, 21.9)	N(17.5, 5)	N(9.1, 6.8)	N(-3.5, 5)	N(-14.8, 26.7)	N(16.1, 5)	N(-18, 27.7)
80	N(16.1, 5)	N(-18, 27.7)	N(24.4, 5)	N(-20.6, 17.2)	N(4.4, 5)	N(-0.2, 9)	N(0.5, 5)	N(-9.3, 20.6)
81	N(16.4, 5)	N(-9.6, 29)	N(16.6, 5)	N(-11.4, 17)	N(-4.4, 5)	N(-11.1, 11.3)	N(-3.5, 5)	N(-14.8, 26.7)
82	N(10.4, 5)	N(-6.7, 12.4)	N(13.2, 5)	N(4, 11.6)	N(16.6, 5)	N(-11.4, 17)	N(17.5, 5)	N(9.1, 6.8)
83	N(0.5, 5)	N(-9.3, 20.6)	N(-11.5, 5)	N(-19.6, 29.6)	N(1.8, 5)	N(-27.2, 10.2)	N(4.5, 5)	N(-25.8, 15.3)
84	N(17.5, 5)	N(9.1, 6.8)	N(-4.2, 5)	N(-8.8, 11.3)	N(16.6, 5)	N(-11.4, 17)	N(47.4, 5)	N(-10.4, 17.7)
85	N(17.5, 5)	N(9.1, 6.8)	N(-20.1, 5)	N(-22.4, 15.4)	N(0.7, 5)	N(-20.6, 21.5)	N(10.2, 5)	N(-7.8, 23.9)
86	N(17.5, 5)	N(9.1, 6.8)	N(10.7, 5)	N(-24.3, 24.7)	N(13.2, 5)	N(4, 11.6)	N(16.4, 5)	N(-9.6, 29)
87	N(-22.1, 5)	N(-36.1, 5.7)	N(16.6, 5)	N(13.6, 10.7)	N(-18.6, 5)	N(-19.6, 23.9)	N(16.1, 5)	N(-18, 27.7)
88	N(21.7, 5)	N(-16.2, 19.6)	N(-0.5, 5)	N(-13.7, 21.9)	N(4.4, 5)	N(-0.2, 9)	N(2.8, 5)	N(-16.3, 17.6)
89	N(2.8, 5)	N(-16.3, 17.6)	N(-0.5, 5)	N(-13.7, 21.9)	N(16.1, 5)	N(9.5, 23.4)	N(14.9, 5)	N(11.9, 14.9)
90	N(23.9, 5)	N(6.1, 20.5)	N(2.2, 5)	N(-2.4, 22.6)	N(-6.8, 5)	N(-20.9, 12.5)	N(0.5, 5)	N(-9.3, 20.6)
91	N(14.2, 5)	N(5.8, 7.7)	N(5.7, 5)	N(2.2, 13.4)	N(10.4, 5)	N(-6.7, 12.4)	N(-3.3, 5)	N(-8.3, 23.1)
92	N(16.1, 5)	N(-18, 27.7)	N(10.9, 5)	N(-9.4, 11.8)	N(21.7, 5)	N(-16.2, 19.6)	N(23.9, 5)	N(6.1, 20.5)
93	N(33.1, 5)	N(-12.6, 15)	N(14.8, 5)	N(2.4, 21.3)	N(22.1, 5)	N(15.5, 18.5)	N(5.7, 5)	N(2.2, 13.4)
94	N(14.9, 5)	N(11.9, 14.9)	N(10.4, 5)	N(-6.7, 12.4)	N(10.9, 5)	N(-9.4, 11.8)	N(-4.4, 5)	N(-11.1, 11.3)
95	N(19, 5)	N(-6, 27)	N(21.7, 5)	N(-16.2, 19.6)	N(33.1, 5)	N(-12.6, 15)	N(-0.5, 5)	N(-13.7, 21.9)
96	N(-3.3, 5)	N(-8.3, 23.1)	N(-20.1, 5)	N(-22.4, 15.4)	N(2.2, 5)	N(-2.4, 22.6)	N(16.1, 5)	N(-18, 27.7)
97	N(24.4, 5)	N(-20.6, 17.2)	N(23.9, 5)	N(6.1, 20.5)	N(16.4, 5)	N(-9.6, 29)	N(0.7, 5)	N(-20.6, 21.5)
98	N(10.4, 5)	N(-2.8, 28)	N(38.7, 5)	N(-4.1, 15.2)	N(47.4, 5)	N(-10.4, 17.7)	N(13.2, 5)	N(4, 11.6)
99	N(-6.8, 5)	N(-20.9, 12.5)	N(13.2, 5)	N(4, 11.6)	N(10.4, 5)	N(-2.8, 28)	N(2.8, 5)	N(-16.3, 17.6)
100	N(5.5, 5)	N(-17.9, 15.3)	N(13.2, 5)	N(4, 11.6)	N(14.2, 5)	N(5.8, 7.7)	N(15.5, 5)	N(-9.9, 25.9)

risk seeking/aversion = 1/3								
	task 1		task 2		task 3		task 4	
simulation	safe	risky	safe	risky	safe	risky	safe	risky
1	N(-7.4, 5)	N(13, 21)	N(16.5, 5)	N(5.2, 17.7)	N(16.5, 5)	N(0.4, 10.2)	N(25, 5)	N(-39.1, 25)
2	N(0.2, 5)	N(6.4, 24.6)	N(8.5, 5)	N(-21.1, 16.8)	N(-8.8, 5)	N(-25.9, 22.5)	N(-2.9, 5)	N(-23, 21.6)
3	N(7.7, 5)	N(12.7, 14.1)	N(-3.3, 5)	N(-17.2, 10.9)	N(-5.8, 5)	N(-24.8, 22.5)	N(0.2, 5)	N(-7.3, 7.4)
4	N(-1.5, 5)	N(23, 7.4)	N(-2.2, 5)	N(-18.2, 21.4)	N(-13.9, 5)	N(-18, 30)	N(-5, 5)	N(-5.1, 17)
5	N(3.4, 5)	N(3.6, 19.5)	N(10.5, 5)	N(-1.2, 24.3)	N(-4.3, 5)	N(-21.2, 19.8)	N(23.4, 5)	N(23.1, 13.5)
6	N(-9.9, 5)	N(23.5, 10.9)	N(-2.2, 5)	N(-18.2, 21.4)	N(-3.3, 5)	N(-17.2, 10.9)	N(10, 5)	N(-3.9, 27)
7	N(-7.2, 5)	N(-0.3, 24.8)	N(6.9, 5)	N(-8.5, 17.1)	N(-6.2, 5)	N(-19.7, 15)	N(16.7, 5)	N(10.7, 11.1)
8	N(-4.3, 5)	N(10.9, 25)	N(16.5, 5)	N(5.2, 17.7)	N(10, 5)	N(-3.9, 27)	N(13.6, 5)	N(8.4, 10.9)
9	N(6.2, 5)	N(19.7, 15)	N(23.4, 5)	N(23.1, 13.5)	N(41.4, 5)	N(-6.1, 26.3)	N(13.6, 5)	N(8.4, 10.9)
10	N(-41.4, 5)	N(6.1, 26.3)	N(-0.2, 5)	N(-6.4, 24.6)	N(14.9, 5)	N(-2.1, 27.4)	N(1.2, 5)	N(-27, 27.8)
11	N(-7.2, 5)	N(-0.3, 24.8)	N(-12.4, 5)	N(-25.2, 23.5)	N(10, 5)	N(-3.9, 27)	N(21, 5)	N(9, 12.2)
12	N(-16.7, 5)	N(-10.7, 11.1)	N(-20.9, 5)	N(-22.9, 19.8)	N(-2.2, 5)	N(-18.2, 21.4)	N(31.1, 5)	N(-1.7, 27.6)
13	N(20.9, 5)	N(22.9, 19.8)	N(12.8, 5)	N(-4.5, 5.6)	N(22.4, 5)	N(2.9, 5.9)	N(-20.9, 5)	N(-22.9, 19.8)
14	N(-25, 5)	N(39.1, 25)	N(14.6, 5)	N(-4.3, 10.2)	N(22.4, 5)	N(2.9, 5.9)	N(9.9, 5)	N(-23.5, 10.9)
15	N(-7.4, 5)	N(13, 21)	N(11.9, 5)	N(6.6, 24.8)	N(22.4, 5)	N(2.9, 5.9)	N(18.9, 5)	N(-9.6, 25.5)
16	N(-6.9, 5)	N(8.5, 17.1)	N(-2.2, 5)	N(-18.2, 21.4)	N(-6.2, 5)	N(-35.9, 14.4)	N(-2.9, 5)	N(-23, 21.6)
17	N(-23.4, 5)	N(-23.1, 13.5)	N(-3.3, 5)	N(-17.2, 10.9)	N(31.1, 5)	N(-1.7, 27.6)	N(13.7, 5)	N(0.8, 11.2)
18	N(-11.9, 5)	N(-6.6, 24.8)	N(41.4, 5)	N(-6.1, 26.3)	N(14.6, 5)	N(-4.3, 10.2)	N(12.7, 5)	N(6.1, 19.1)
19	N(12.4, 5)	N(25.2, 23.5)	N(16.5, 5)	N(0.4, 10.2)	N(7.4, 5)	N(-13, 21)	N(1.2, 5)	N(-27, 27.8)
20	N(-9.9, 5)	N(23.5, 10.9)	N(16.7, 5)	N(10.7, 11.1)	N(4.3, 5)	N(-10.9, 25)	N(1.2, 5)	N(-27, 27.8)
21	N(-22.4, 5)	N(-2.9, 5.9)	N(-5.8, 5)	N(-24.8, 22.5)	N(0.2, 5)	N(-7.3, 7.4)	N(6.9, 5)	N(-8.5, 17.1)
22	N(4.3, 5)	N(21.2, 19.8)	N(23.1, 5)	N(-0.4, 16.8)	N(8.5, 5)	N(-21.1, 16.8)	N(13.6, 5)	N(8.4, 10.9)
23	N(-14.9, 5)	N(2.1, 27.4)	N(11.9, 5)	N(6.6, 24.8)	N(1.5, 5)	N(-23, 7.4)	N(16.7, 5)	N(10.7, 11.1)
24	N(-13.6, 5)	N(-8.4, 10.9)	N(-6.2, 5)	N(-19.7, 15)	N(-3.3, 5)	N(-17.2, 10.9)	N(-3.4, 5)	N(-3.6, 19.5)
25	N(-16.7, 5)	N(-10.7, 11.1)	N(11.9, 5)	N(6.6, 24.8)	N(18.9, 5)	N(-9.6, 25.5)	N(-5, 5)	N(-5.1, 17)
26	N(-6.9, 5)	N(8.5, 17.1)	N(7.4, 5)	N(-13, 21)	N(-2.2, 5)	N(-18.2, 21.4)	N(16.5, 5)	N(-24, 29.4)
27	N(-4.3, 5)	N(10.9, 25)	N(-6.2, 5)	N(-35.9, 14.4)	N(1.5, 5)	N(-23, 7.4)	N(10.5, 5)	N(-1.2, 24.3)
28	N(7.7, 5)	N(12.7, 14.1)	N(7.4, 5)	N(-13, 21)	N(-2.9, 5)	N(-23, 21.6)	N(-20.9, 5)	N(-22.9, 19.8)
29	N(-14.9, 5)	N(2.1, 27.4)	N(-7.7, 5)	N(-12.7, 14.1)	N(-6.2, 5)	N(-19.7, 15)	N(-3.3, 5)	N(-17.2, 10.9)
30	N(5, 5)	N(5.1, 17)	N(13.6, 5)	N(8.4, 10.9)	N(16.7, 5)	N(10.7, 11.1)	N(23.4, 5)	N(23.1, 13.5)
31	N(-16.5, 5)	N(-5.2, 17.7)	N(-13.9, 5)	N(-18, 30)	N(25, 5)	N(-39.1, 25)	N(9.9, 5)	N(-23.5, 10.9)
32	N(-31.1, 5)	N(1.7, 27.6)	N(16.5, 5)	N(-24, 29.4)	N(-6.2, 5)	N(-35.9, 14.4)	N(13.6, 5)	N(8.4, 10.9)
33	N(0.4, 5)	N(30.3, 24.9)	N(10.5, 5)	N(-1.2, 24.3)	N(11.9, 5)	N(6.6, 24.8)	N(9.9, 5)	N(-23.5, 10.9)
34	N(6.2, 5)	N(35.9, 14.4)	N(14.9, 5)	N(-2.1, 27.4)	N(11.9, 5)	N(6.6, 24.8)	N(12.8, 5)	N(-4.5, 5.6)
35	N(0.4, 5)	N(30.3, 24.9)	N(4.3, 5)	N(-10.9, 25)	N(7.4, 5)	N(-13, 21)	N(13.6, 5)	N(8.4, 10.9)
36	N(-16.5, 5)	N(-5.2, 17.7)	N(10, 5)	N(-3.9, 27)	N(16.5, 5)	N(0.4, 10.2)	N(-2.9, 5)	N(-23, 21.6)
37	N(-8.5, 5)	N(21.1, 16.8)	N(-4.3, 5)	N(-21.2, 19.8)	N(14.6, 5)	N(-4.3, 10.2)	N(-6.2, 5)	N(-35.9, 14.4)
38	N(20.9, 5)	N(22.9, 19.8)	N(4.3, 5)	N(-10.9, 25)	N(23.1, 5)	N(-0.4, 16.8)	N(7.2, 5)	N(0.3, 24.8)
39	N(-23.1, 5)	N(0.4, 16.8)	N(-3.4, 5)	N(-3.6, 19.5)	N(23.1, 5)	N(-0.4, 16.8)	N(8.5, 5)	N(-21.1, 16.8)
40	N(3.4, 5)	N(3.6, 19.5)	N(16.5, 5)	N(0.4, 10.2)	N(10.5, 5)	N(-1.2, 24.3)	N(9.2, 5)	N(-12, 15.6)
41	N(2.9, 5)	N(23, 21.6)	N(-13.8, 5)	N(-16.9, 21.5)	N(4.3, 5)	N(-10.9, 25)	N(21, 5)	N(9, 12.2)
42	N(-16.5, 5)	N(-0.4, 10.2)	N(13.7, 5)	N(0.8, 11.2)	N(9.9, 5)	N(-23.5, 10.9)	N(34.7, 5)	N(-4.1, 24.8)
43	N(-19.5, 5)	N(-1.9, 29.4)	N(-0.4, 5)	N(-30.3, 24.9)	N(-8.8, 5)	N(-25.9, 22.5)	N(-5, 5)	N(-5.1, 17)
44	N(-7.2, 5)	N(-0.3, 24.8)	N(9.9, 5)	N(-23.5, 10.9)	N(21, 5)	N(9, 12.2)	N(-5, 5)	N(-5.1, 17)
45	N(-21, 5)	N(-9, 12.2)	N(21, 5)	N(9, 12.2)	N(-5, 5)	N(-5.1, 17)	N(19.5, 5)	N(1.9, 29.4)
46	N(-16.7, 5)	N(-10.7, 11.1)	N(10.5, 5)	N(-1.2, 24.3)	N(-4.3, 5)	N(-21.2, 19.8)	N(-2.2, 5)	N(-18.2, 21.4)
47	N(-23.1, 5)	N(0.4, 16.8)	N(23.1, 5)	N(-0.4, 16.8)	N(1.5, 5)	N(-23, 7.4)	N(13.6, 5)	N(8.4, 10.9)
48	N(-1.2, 5)	N(27, 27.8)	N(11.9, 5)	N(6.6, 24.8)	N(-2.2, 5)	N(-18.2, 21.4)	N(10, 5)	N(-3.9, 27)
49	N(-31.1, 5)	N(1.7, 27.6)	N(18.9, 5)	N(-9.6, 25.5)	N(-7.7, 5)	N(-12.7, 14.1)	N(-13.8, 5)	N(-16.9, 21.5)
50	N(-41.4, 5)	N(6.1, 26.3)	N(-3.3, 5)	N(-17.2, 10.9)	N(-6.2, 5)	N(-35.9, 14.4)	N(22.4, 5)	N(2.9, 5.9)

51	N(-16.7, 5)	N(-10.7, 11.1)	N(-12.4, 5)	N(-25.2, 23.5)	N(-13.8, 5)	N(-16.9, 21.5)	N(11.9, 5)	N(6.6, 24.8)
52	N(-16.5, 5)	N(-5.2, 17.7)	N(25, 5)	N(-39.1, 25)	N(16.5, 5)	N(5.2, 17.7)	N(12.8, 5)	N(-4.5, 5.6)
53	N(3.3, 5)	N(17.2, 10.9)	N(14.9, 5)	N(-2.1, 27.4)	N(4.3, 5)	N(-10.9, 25)	N(-0.2, 5)	N(-6.4, 24.6)
54	N(-13.7, 5)	N(-0.8, 11.2)	N(-20.9, 5)	N(-22.9, 19.8)	N(-3.3, 5)	N(-17.2, 10.9)	N(1.5, 5)	N(-23, 7.4)
55	N(0.2, 5)	N(6.4, 24.6)	N(-3.4, 5)	N(-3.6, 19.5)	N(9.2, 5)	N(-12, 15.6)	N(16.7, 5)	N(10.7, 11.1)
56	N(-25, 5)	N(39.1, 25)	N(-12.4, 5)	N(-25.2, 23.5)	N(34.7, 5)	N(-4.1, 24.8)	N(7.2, 5)	N(0.3, 24.8)
57	N(-34.7, 5)	N(4.1, 24.8)	N(-2.2, 5)	N(-18.2, 21.4)	N(-6.2, 5)	N(-35.9, 14.4)	N(41.4, 5)	N(-6.1, 26.3)
58	N(-11.9, 5)	N(-6.6, 24.8)	N(1.2, 5)	N(-27, 27.8)	N(-8.8, 5)	N(-25.9, 22.5)	N(25, 5)	N(-39.1, 25)
59	N(13.9, 5)	N(18, 30)	N(1.2, 5)	N(-27, 27.8)	N(12.8, 5)	N(-4.5, 5.6)	N(11.9, 5)	N(6.6, 24.8)
60	N(-7.4, 5)	N(13, 21)	N(34.7, 5)	N(-4.1, 24.8)	N(7.4, 5)	N(-13, 21)	N(13.6, 5)	N(8.4, 10.9)
61	N(-1.5, 5)	N(23, 7.4)	N(23.4, 5)	N(23.1, 13.5)	N(-6.2, 5)	N(-35.9, 14.4)	N(14.9, 5)	N(-2.1, 27.4)
62	N(-11.9, 5)	N(-6.6, 24.8)	N(19.5, 5)	N(1.9, 29.4)	N(13.6, 5)	N(8.4, 10.9)	N(-2.9, 5)	N(-23, 21.6)
63	N(-6.9, 5)	N(8.5, 17.1)	N(-2.9, 5)	N(-23, 21.6)	N(14.9, 5)	N(-2.1, 27.4)	N(22.4, 5)	N(2.9, 5.9)
64	N(-22.4, 5)	N(-2.9, 5.9)	N(19.5, 5)	N(1.9, 29.4)	N(1.5, 5)	N(-23, 7.4)	N(14.6, 5)	N(-4.3, 10.2)
65	N(-16.5, 5)	N(24, 29.4)	N(-13.8, 5)	N(-16.9, 21.5)	N(-13.9, 5)	N(-18, 30)	N(12.8, 5)	N(-4.5, 5.6)
66	N(13.8, 5)	N(16.9, 21.5)	N(16.7, 5)	N(10.7, 11.1)	N(-0.2, 5)	N(-6.4, 24.6)	N(4.3, 5)	N(-10.9, 25)
67	N(-34.7, 5)	N(4.1, 24.8)	N(1.5, 5)	N(-23, 7.4)	N(4.3, 5)	N(-10.9, 25)	N(-20.9, 5)	N(-22.9, 19.8)
68	N(6.2, 5)	N(35.9, 14.4)	N(14.6, 5)	N(-4.3, 10.2)	N(-4.3, 5)	N(-21.2, 19.8)	N(13.7, 5)	N(0.8, 11.2)
69	N(2.2, 5)	N(18.2, 21.4)	N(16.5, 5)	N(5.2, 17.7)	N(7.2, 5)	N(0.3, 24.8)	N(-12.4, 5)	N(-25.2, 23.5)
70	N(-9.9, 5)	N(23.5, 10.9)	N(21, 5)	N(9, 12.2)	N(10.5, 5)	N(-1.2, 24.3)	N(16.5, 5)	N(5.2, 17.7)
71	N(-12.7, 5)	N(-6.1, 19.1)	N(-4.3, 5)	N(-21.2, 19.8)	N(21, 5)	N(9, 12.2)	N(8.5, 5)	N(-21.1, 16.8)
72	N(13.9, 5)	N(18, 30)	N(-5, 5)	N(-5.1, 17)	N(23.4, 5)	N(23.1, 13.5)	N(14.6, 5)	N(-4.3, 10.2)
73	N(0.4, 5)	N(30.3, 24.9)	N(-3.3, 5)	N(-17.2, 10.9)	N(-2.2, 5)	N(-18.2, 21.4)	N(14.6, 5)	N(-4.3, 10.2)
74	N(-16.5, 5)	N(24, 29.4)	N(21, 5)	N(9, 12.2)	N(14.6, 5)	N(-4.3, 10.2)	N(23.4, 5)	N(23.1, 13.5)
75	N(5.8, 5)	N(24.8, 22.5)	N(-13.8, 5)	N(-16.9, 21.5)	N(-7.7, 5)	N(-12.7, 14.1)	N(-20.9, 5)	N(-22.9, 19.8)
76	N(-22.4, 5)	N(-2.9, 5.9)	N(-6.2, 5)	N(-19.7, 15)	N(19.5, 5)	N(1.9, 29.4)	N(6.9, 5)	N(-8.5, 17.1)
77	N(20.9, 5)	N(22.9, 19.8)	N(9.2, 5)	N(-12, 15.6)	N(-20.9, 5)	N(-22.9, 19.8)	N(16.5, 5)	N(5.2, 17.7)
78	N(-8.5, 5)	N(21.1, 16.8)	N(1.5, 5)	N(-23, 7.4)	N(12.8, 5)	N(-4.5, 5.6)	N(23.4, 5)	N(23.1, 13.5)
79	N(-16.5, 5)	N(24, 29.4)	N(-0.4, 5)	N(-30.3, 24.9)	N(-2.2, 5)	N(-18.2, 21.4)	N(1.5, 5)	N(-23, 7.4)
80	N(-6.9, 5)	N(8.5, 17.1)	N(18.9, 5)	N(-9.6, 25.5)	N(-7.7, 5)	N(-12.7, 14.1)	N(12.7, 5)	N(6.1, 19.1)
81	N(5.8, 5)	N(24.8, 22.5)	N(8.5, 5)	N(-21.1, 16.8)	N(14.9, 5)	N(-2.1, 27.4)	N(13.7, 5)	N(0.8, 11.2)
82	N(-18.9, 5)	N(9.6, 25.5)	N(-12.4, 5)	N(-25.2, 23.5)	N(-0.4, 5)	N(-30.3, 24.9)	N(34.7, 5)	N(-4.1, 24.8)
83	N(-22.4, 5)	N(-2.9, 5.9)	N(-2.9, 5)	N(-23, 21.6)	N(-4.3, 5)	N(-21.2, 19.8)	N(-20.9, 5)	N(-22.9, 19.8)
84	N(-18.9, 5)	N(9.6, 25.5)	N(7.4, 5)	N(-13, 21)	N(4.3, 5)	N(-10.9, 25)	N(-5.8, 5)	N(-24.8, 22.5)
85	N(-16.5, 5)	N(-5.2, 17.7)	N(31.1, 5)	N(-1.7, 27.6)	N(23.4, 5)	N(23.1, 13.5)	N(14.6, 5)	N(-4.3, 10.2)
86	N(7.7, 5)	N(12.7, 14.1)	N(-3.3, 5)	N(-17.2, 10.9)	N(6.9, 5)	N(-8.5, 17.1)	N(22.4, 5)	N(2.9, 5.9)
87	N(6.2, 5)	N(19.7, 15)	N(7.2, 5)	N(0.3, 24.8)	N(10.5, 5)	N(-1.2, 24.3)	N(34.7, 5)	N(-4.1, 24.8)
88	N(4.3, 5)	N(21.2, 19.8)	N(19.5, 5)	N(1.9, 29.4)	N(-13.8, 5)	N(-16.9, 21.5)	N(-2.2, 5)	N(-18.2, 21.4)
89	N(7.7, 5)	N(12.7, 14.1)	N(-6.2, 5)	N(-35.9, 14.4)	N(16.5, 5)	N(0.4, 10.2)	N(16.5, 5)	N(-24, 29.4)
90	N(-23.1, 5)	N(0.4, 16.8)	N(1.5, 5)	N(-23, 7.4)	N(-5, 5)	N(-5.1, 17)	N(-7.7, 5)	N(-12.7, 14.1)
91	N(6.2, 5)	N(19.7, 15)	N(6.9, 5)	N(-8.5, 17.1)	N(1.5, 5)	N(-23, 7.4)	N(16.7, 5)	N(10.7, 11.1)
92	N(-16.5, 5)	N(-0.4, 10.2)	N(1.5, 5)	N(-23, 7.4)	N(1.2, 5)	N(-27, 27.8)	N(-0.2, 5)	N(-6.4, 24.6)
93	N(-18.9, 5)	N(9.6, 25.5)	N(1.2, 5)	N(-27, 27.8)	N(-2.9, 5)	N(-23, 21.6)	N(-13.9, 5)	N(-18, 30)
94	N(-10.5, 5)	N(1.2, 24.3)	N(41.4, 5)	N(-6.1, 26.3)	N(9.2, 5)	N(-12, 15.6)	N(-20.9, 5)	N(-22.9, 19.8)
95	N(-12.7, 5)	N(-6.1, 19.1)	N(16.5, 5)	N(5.2, 17.7)	N(-0.2, 5)	N(-6.4, 24.6)	N(21, 5)	N(9, 12.2)
96	N(-16.5, 5)	N(-0.4, 10.2)	N(0.2, 5)	N(-7.3, 7.4)	N(11.9, 5)	N(6.6, 24.8)	N(6.9, 5)	N(-8.5, 17.1)
97	N(-41.4, 5)	N(6.1, 26.3)	N(41.4, 5)	N(-6.1, 26.3)	N(-8.8, 5)	N(-25.9, 22.5)	N(12.8, 5)	N(-4.5, 5.6)
98	N(20.9, 5)	N(22.9, 19.8)	N(14.6, 5)	N(-4.3, 10.2)	N(31.1, 5)	N(-1.7, 27.6)	N(16.7, 5)	N(10.7, 11.1)
99	N(0.2, 5)	N(6.4, 24.6)	N(-12.4, 5)	N(-25.2, 23.5)	N(-2.9, 5)	N(-23, 21.6)	N(9.9, 5)	N(-23.5, 10.9)
100	N(-14.6, 5)	N(4.3, 10.2)	N(9.2, 5)	N(-12, 15.6)	N(31.1, 5)	N(-1.7, 27.6)	N(-5.8, 5)	N(-24.8, 22.5)

risk seeking/aversion = 2/2								
	task 1		task 2		task 3		task 4	
simulation	safe	risky	safe	risky	safe	risky	safe	risky
1	N(4, 5)	N(18.3, 21.4)	N(13.7, 5)	N(21.3, 8.3)	N(19.1, 5)	N(3.8, 13.3)	N(2.7, 5)	N(-28.1, 20.7)
2	N(15.2, 5)	N(31.2, 21.2)	N(6.6, 5)	N(33.9, 9.8)	N(11.7, 5)	N(-17, 27.1)	N(29, 5)	N(-4, 14.9)
3	N(-2.6, 5)	N(9.5, 11.2)	N(-3.8, 5)	N(-2.4, 8.7)	N(-15.2, 5)	N(-31.2, 21.2)	N(-16.3, 5)	N(-25.6, 25)
4	N(-15.5, 5)	N(24.1, 25.6)	N(-22.9, 5)	N(-0.4, 22.5)	N(23.6, 5)	N(-11.7, 25.7)	N(14.1, 5)	N(-19.7, 28.9)
5	N(-15.8, 5)	N(29.2, 27.8)	N(-22.9, 5)	N(-0.4, 22.5)	N(-24.2, 5)	N(-26.6, 5.5)	N(24.5, 5)	N(19.6, 14.2)
6	N(-13.5, 5)	N(7.9, 24.1)	N(-11.1, 5)	N(2.8, 11.6)	N(32.8, 5)	N(13.2, 19.2)	N(18.9, 5)	N(-16, 26.9)
7	N(24.2, 5)	N(26.6, 5.5)	N(-3.8, 5)	N(-2.4, 8.7)	N(-6.7, 5)	N(-12.4, 19.9)	N(11.7, 5)	N(-17, 27.1)
8	N(-11.3, 5)	N(-6.3, 22.8)	N(-2.7, 5)	N(28.1, 20.7)	N(-4.8, 5)	N(-34.3, 25.9)	N(-16.3, 5)	N(-25.6, 25)
9	N(15.2, 5)	N(31.2, 21.2)	N(-2.7, 5)	N(28.1, 20.7)	N(33, 5)	N(11.9, 10.7)	N(-8.8, 5)	N(-11.9, 29.5)
10	N(6.7, 5)	N(12.4, 19.9)	N(-33, 5)	N(-11.9, 10.7)	N(11.7, 5)	N(-17, 27.1)	N(10.3, 5)	N(-15.9, 25.2)
11	N(-7.9, 5)	N(10.1, 27.6)	N(-9.5, 5)	N(26.2, 16)	N(15.8, 5)	N(-29.2, 27.8)	N(1.6, 5)	N(-17, 5.9)
12	N(-14.8, 5)	N(-14.4, 8.1)	N(-24.5, 5)	N(-19.6, 14.2)	N(11.4, 5)	N(10, 21.1)	N(-24.2, 5)	N(-26.6, 5.5)
13	N(-33.1, 5)	N(35.7, 26.7)	N(-14.1, 5)	N(19.7, 28.9)	N(9.5, 5)	N(-26.2, 16)	N(-2.7, 5)	N(-23.6, 18.4)
14	N(-15.8, 5)	N(29.2, 27.8)	N(-1.6, 5)	N(17, 5.9)	N(-2.8, 5)	N(-20.8, 8.7)	N(11.1, 5)	N(-2.8, 11.6)
15	N(-19.4, 5)	N(33.9, 19.4)	N(4, 5)	N(18.3, 21.4)	N(-2.8, 5)	N(-20.8, 8.7)	N(19.1, 5)	N(3.8, 13.3)
16	N(-7.9, 5)	N(10.1, 27.6)	N(6.7, 5)	N(12.4, 19.9)	N(17.8, 5)	N(-9.8, 12.7)	N(14.1, 5)	N(-19.7, 28.9)
17	N(-14.1, 5)	N(19.7, 28.9)	N(35, 5)	N(49.7, 9.4)	N(3.2, 5)	N(1.5, 27.4)	N(14.1, 5)	N(-19.7, 28.9)
18	N(2.8, 5)	N(20.8, 8.7)	N(-9.4, 5)	N(-6.6, 20)	N(-2.7, 5)	N(-23.6, 18.4)	N(15.3, 5)	N(-4, 23.6)
19	N(-33, 5)	N(-11.9, 10.7)	N(-9.5, 5)	N(26.2, 16)	N(11.1, 5)	N(-2.8, 11.6)	N(15.3, 5)	N(-4, 23.6)
20	N(-10.3, 5)	N(15.9, 25.2)	N(4.2, 5)	N(21.7, 26.6)	N(-6.7, 5)	N(-12.4, 19.9)	N(15.3, 5)	N(-4, 23.6)
21	N(6.6, 5)	N(33.9, 9.8)	N(6.7, 5)	N(12.4, 19.9)	N(11.7, 5)	N(-17, 27.1)	N(33, 5)	N(11.9, 10.7)
22	N(6.7, 5)	N(12.4, 19.9)	N(-15.3, 5)	N(4, 23.6)	N(-4.8, 5)	N(-34.3, 25.9)	N(10.3, 5)	N(-15.9, 25.2)
23	N(-14.1, 5)	N(19.7, 28.9)	N(-4.3, 5)	N(15.3, 8.7)	N(11.4, 5)	N(10, 21.1)	N(-13.7, 5)	N(-21.3, 8.3)
24	N(-19.1, 5)	N(-3.8, 13.3)	N(4.2, 5)	N(21.7, 26.6)	N(-16.3, 5)	N(-25.6, 25)	N(10.3, 5)	N(-15.9, 25.2)
25	N(16.3, 5)	N(25.6, 25)	N(-10.3, 5)	N(15.9, 25.2)	N(15.3, 5)	N(-4, 23.6)	N(-15.2, 5)	N(-31.2, 21.2)
26	N(4.8, 5)	N(34.3, 25.9)	N(-18.9, 5)	N(16, 26.9)	N(17.8, 5)	N(-9.8, 12.7)	N(3.2, 5)	N(1.5, 27.4)
27	N(-18.9, 5)	N(16, 26.9)	N(-9.5, 5)	N(26.2, 16)	N(-4.2, 5)	N(-21.7, 26.6)	N(4.3, 5)	N(-15.3, 8.7)
28	N(-11.4, 5)	N(-10, 21.1)	N(-4.5, 5)	N(8.3, 9.5)	N(-2.8, 5)	N(-20.8, 8.7)	N(-8.8, 5)	N(-11.9, 29.5)
29	N(8.8, 5)	N(11.9, 29.5)	N(-9.5, 5)	N(26.2, 16)	N(23.6, 5)	N(-11.7, 25.7)	N(6.4, 5)	N(-14.8, 18.9)
30	N(-4.3, 5)	N(15.3, 8.7)	N(-23.6, 5)	N(11.7, 25.7)	N(32.8, 5)	N(13.2, 19.2)	N(-4, 5)	N(-18.3, 21.4)
31	N(-15.5, 5)	N(24.1, 25.6)	N(-19.1, 5)	N(-3.8, 13.3)	N(17.8, 5)	N(-9.8, 12.7)	N(24.5, 5)	N(19.6, 14.2)
32	N(-14.1, 5)	N(19.7, 28.9)	N(-32.8, 5)	N(-13.2, 19.2)	N(11.1, 5)	N(-2.8, 11.6)	N(9.4, 5)	N(6.6, 20)
33	N(2.8, 5)	N(20.8, 8.7)	N(-19.1, 5)	N(-3.8, 13.3)	N(-16.3, 5)	N(-25.6, 25)	N(-35, 5)	N(-49.7, 9.4)
34	N(35, 5)	N(49.7, 9.4)	N(4.8, 5)	N(34.3, 25.9)	N(11.7, 5)	N(-17, 27.1)	N(6.4, 5)	N(-14.8, 18.9)
35	N(-15.8, 5)	N(29.2, 27.8)	N(4.8, 5)	N(34.3, 25.9)	N(11.4, 5)	N(10, 21.1)	N(-2.7, 5)	N(-23.6, 18.4)
36	N(6.6, 5)	N(33.9, 9.8)	N(18.1, 5)	N(19.7, 12)	N(23.6, 5)	N(-11.7, 25.7)	N(17.8, 5)	N(-9.8, 12.7)
37	N(-4.5, 5)	N(8.3, 9.5)	N(-23.6, 5)	N(11.7, 25.7)	N(24.5, 5)	N(19.6, 14.2)	N(-4.2, 5)	N(-21.7, 26.6)
38	N(-9.4, 5)	N(-6.6, 20)	N(6.7, 5)	N(12.4, 19.9)	N(32.8, 5)	N(13.2, 19.2)	N(-24.2, 5)	N(-26.6, 5.5)
39	N(-23.5, 5)	N(2.2, 9.3)	N(-4.5, 5)	N(8.3, 9.5)	N(15.3, 5)	N(-4, 23.6)	N(13.5, 5)	N(-7.9, 24.1)
40	N(18.1, 5)	N(19.7, 12)	N(-23.6, 5)	N(11.7, 25.7)	N(15.8, 5)	N(-29.2, 27.8)	N(5.2, 5)	N(0, 11.1)
41	N(-15.5, 5)	N(24.1, 25.6)	N(-5.2, 5)	N(0, 11.1)	N(11.3, 5)	N(6.3, 22.8)	N(30.2, 5)	N(27.4, 23.6)
42	N(2.8, 5)	N(20.8, 8.7)	N(-17.8, 5)	N(9.8, 12.7)	N(-2.7, 5)	N(-23.6, 18.4)	N(3.8, 5)	N(2.4, 8.7)
43	N(-11.7, 5)	N(17, 27.1)	N(-19.4, 5)	N(33.9, 19.4)	N(11.7, 5)	N(-17, 27.1)	N(4.3, 5)	N(-15.3, 8.7)
44	N(-13.5, 5)	N(7.9, 24.1)	N(-11.3, 5)	N(-6.3, 22.8)	N(13.5, 5)	N(-7.9, 24.1)	N(3.8, 5)	N(2.4, 8.7)
45	N(-3.2, 5)	N(-1.5, 27.4)	N(-29, 5)	N(4, 14.9)	N(18.9, 5)	N(-16, 26.9)	N(22.9, 5)	N(0.4, 22.5)
46	N(-13.5, 5)	N(7.9, 24.1)	N(-15.8, 5)	N(29.2, 27.8)	N(22.9, 5)	N(0.4, 22.5)	N(3.2, 5)	N(1.5, 27.4)
47	N(13.7, 5)	N(21.3, 8.3)	N(-15.5, 5)	N(24.1, 25.6)	N(-18.1, 5)	N(-19.7, 12)	N(-4.2, 5)	N(-21.7, 26.6)
48	N(-30.2, 5)	N(-27.4, 23.6)	N(-11.1, 5)	N(2.8, 11.6)	N(2.6, 5)	N(-9.5, 11.2)	N(19.1, 5)	N(3.8, 13.3)
49	N(16.3, 5)	N(25.6, 25)	N(-14.8, 5)	N(-14.4, 8.1)	N(-6.7, 5)	N(-12.4, 19.9)	N(13.5, 5)	N(-7.9, 24.1)
50	N(16.3, 5)	N(25.6, 25)	N(2.7, 5)	N(23.6, 18.4)	N(-6.6, 5)	N(-33.9, 9.8)	N(30.2, 5)	N(27.4, 23.6)

51	N(-13.5, 5)	N(7.9, 24.1)	N(-30.2, 5)	N(-27.4, 23.6)	N(2.6, 5)	N(-9.5, 11.2)	N(6.4, 5)	N(-14.8, 18.9)
52	N(-30.2, 5)	N(-27.4, 23.6)	N(24.2, 5)	N(26.6, 5.5)	N(-4, 5)	N(-18.3, 21.4)	N(10.3, 5)	N(-15.9, 25.2)
53	N(6.7, 5)	N(12.4, 19.9)	N(-29, 5)	N(4, 14.9)	N(14.1, 5)	N(-19.7, 28.9)	N(15.5, 5)	N(-24.1, 25.6)
54	N(-3.2, 5)	N(-1.5, 27.4)	N(-14.1, 5)	N(19.7, 28.9)	N(-24.2, 5)	N(-26.6, 5.5)	N(11.1, 5)	N(-2.8, 11.6)
55	N(-19.4, 5)	N(33.9, 19.4)	N(15.2, 5)	N(31.2, 21.2)	N(4.3, 5)	N(-15.3, 8.7)	N(33.1, 5)	N(-35.7, 26.7)
56	N(-3.2, 5)	N(-1.5, 27.4)	N(-11.3, 5)	N(-6.3, 22.8)	N(2.6, 5)	N(-9.5, 11.2)	N(19.4, 5)	N(-33.9, 19.4)
57	N(24.2, 5)	N(26.6, 5.5)	N(-3.8, 5)	N(-2.4, 8.7)	N(-6.7, 5)	N(-12.4, 19.9)	N(10.3, 5)	N(-15.9, 25.2)
58	N(-24.5, 5)	N(-19.6, 14.2)	N(18.1, 5)	N(19.7, 12)	N(-13.7, 5)	N(-21.3, 8.3)	N(14.8, 5)	N(14.4, 8.1)
59	N(-24.5, 5)	N(-19.6, 14.2)	N(-7.9, 5)	N(10.1, 27.6)	N(-8.8, 5)	N(-11.9, 29.5)	N(1.6, 5)	N(-17, 5.9)
60	N(35, 5)	N(49.7, 9.4)	N(-1.6, 5)	N(17, 5.9)	N(30.2, 5)	N(27.4, 23.6)	N(19.1, 5)	N(3.8, 13.3)
61	N(4.8, 5)	N(34.3, 25.9)	N(8.8, 5)	N(11.9, 29.5)	N(19.1, 5)	N(3.8, 13.3)	N(17.8, 5)	N(-9.8, 12.7)
62	N(-17.8, 5)	N(9.8, 12.7)	N(-3.2, 5)	N(-1.5, 27.4)	N(-4, 5)	N(-18.3, 21.4)	N(-13.7, 5)	N(-21.3, 8.3)
63	N(35, 5)	N(49.7, 9.4)	N(-5.2, 5)	N(0, 11.1)	N(-13.7, 5)	N(-21.3, 8.3)	N(-35, 5)	N(-49.7, 9.4)
64	N(-11.1, 5)	N(2.8, 11.6)	N(16.3, 5)	N(25.6, 25)	N(-13.7, 5)	N(-21.3, 8.3)	N(-16.3, 5)	N(-25.6, 25)
65	N(35, 5)	N(49.7, 9.4)	N(13.7, 5)	N(21.3, 8.3)	N(-2.8, 5)	N(-20.8, 8.7)	N(11.1, 5)	N(-2.8, 11.6)
66	N(-3.2, 5)	N(-1.5, 27.4)	N(-23.6, 5)	N(11.7, 25.7)	N(-8.8, 5)	N(-11.9, 29.5)	N(-13.7, 5)	N(-21.3, 8.3)
67	N(-33.1, 5)	N(35.7, 26.7)	N(4, 5)	N(18.3, 21.4)	N(15.3, 5)	N(-4, 23.6)	N(33, 5)	N(11.9, 10.7)
68	N(4.8, 5)	N(34.3, 25.9)	N(2.8, 5)	N(20.8, 8.7)	N(33, 5)	N(11.9, 10.7)	N(10.3, 5)	N(-15.9, 25.2)
69	N(18.1, 5)	N(19.7, 12)	N(-1.6, 5)	N(17, 5.9)	N(11.7, 5)	N(-17, 27.1)	N(1.6, 5)	N(-17, 5.9)
70	N(-3.2, 5)	N(-1.5, 27.4)	N(-2.6, 5)	N(9.5, 11.2)	N(11.3, 5)	N(6.3, 22.8)	N(4.3, 5)	N(-15.3, 8.7)
71	N(-15.8, 5)	N(29.2, 27.8)	N(-9.5, 5)	N(26.2, 16)	N(-35, 5)	N(-49.7, 9.4)	N(11.4, 5)	N(10, 21.1)
72	N(-19.4, 5)	N(33.9, 19.4)	N(-5.2, 5)	N(0, 11.1)	N(15.3, 5)	N(-4, 23.6)	N(-35, 5)	N(-49.7, 9.4)
73	N(8.8, 5)	N(11.9, 29.5)	N(4, 5)	N(18.3, 21.4)	N(11.4, 5)	N(10, 21.1)	N(-15.2, 5)	N(-31.2, 21.2)
74	N(-14.1, 5)	N(19.7, 28.9)	N(-5.2, 5)	N(0, 11.1)	N(-35, 5)	N(-49.7, 9.4)	N(-4, 5)	N(-18.3, 21.4)
75	N(8.8, 5)	N(11.9, 29.5)	N(-22.9, 5)	N(-0.4, 22.5)	N(-13.7, 5)	N(-21.3, 8.3)	N(-24.2, 5)	N(-26.6, 5.5)
76	N(35, 5)	N(49.7, 9.4)	N(6.6, 5)	N(33.9, 9.8)	N(-2.7, 5)	N(-23.6, 18.4)	N(1.6, 5)	N(-17, 5.9)
77	N(-4.5, 5)	N(8.3, 9.5)	N(35, 5)	N(49.7, 9.4)	N(19.1, 5)	N(3.8, 13.3)	N(2.7, 5)	N(-28.1, 20.7)
78	N(-11.7, 5)	N(17, 27.1)	N(-23.6, 5)	N(11.7, 25.7)	N(2.6, 5)	N(-9.5, 11.2)	N(2.7, 5)	N(-28.1, 20.7)
79	N(-4.5, 5)	N(8.3, 9.5)	N(-23.6, 5)	N(11.7, 25.7)	N(11.3, 5)	N(6.3, 22.8)	N(6.4, 5)	N(-14.8, 18.9)
80	N(-30.2, 5)	N(-27.4, 23.6)	N(-10.3, 5)	N(15.9, 25.2)	N(-4.8, 5)	N(-34.3, 25.9)	N(-15.2, 5)	N(-31.2, 21.2)
81	N(2.7, 5)	N(23.6, 18.4)	N(6.6, 5)	N(33.9, 9.8)	N(11.7, 5)	N(-17, 27.1)	N(2.7, 5)	N(-28.1, 20.7)
82	N(-15.5, 5)	N(24.1, 25.6)	N(-7.9, 5)	N(10.1, 27.6)	N(3.2, 5)	N(1.5, 27.4)	N(5.2, 5)	N(0, 11.1)
83	N(-7.9, 5)	N(10.1, 27.6)	N(-6.4, 5)	N(14.8, 18.9)	N(15.3, 5)	N(-4, 23.6)	N(-4.8, 5)	N(-34.3, 25.9)
84	N(15.2, 5)	N(31.2, 21.2)	N(-2.7, 5)	N(28.1, 20.7)	N(19.1, 5)	N(3.8, 13.3)	N(-4.8, 5)	N(-34.3, 25.9)
85	N(-19.1, 5)	N(-3.8, 13.3)	N(-11.3, 5)	N(-6.3, 22.8)	N(-35, 5)	N(-49.7, 9.4)	N(9.4, 5)	N(6.6, 20)
86	N(-15.5, 5)	N(24.1, 25.6)	N(8.8, 5)	N(11.9, 29.5)	N(32.8, 5)	N(13.2, 19.2)	N(-18.1, 5)	N(-19.7, 12)
87	N(-3.2, 5)	N(-1.5, 27.4)	N(-5.2, 5)	N(0, 11.1)	N(6.4, 5)	N(-14.8, 18.9)	N(-4.2, 5)	N(-21.7, 26.6)
88	N(-11.7, 5)	N(17, 27.1)	N(16.3, 5)	N(25.6, 25)	N(3.8, 5)	N(2.4, 8.7)	N(2.7, 5)	N(-28.1, 20.7)
89	N(-32.8, 5)	N(-13.2, 19.2)	N(-14.1, 5)	N(19.7, 28.9)	N(33.1, 5)	N(-35.7, 26.7)	N(-4, 5)	N(-18.3, 21.4)
90	N(-3.8, 5)	N(-2.4, 8.7)	N(15.2, 5)	N(31.2, 21.2)	N(30.2, 5)	N(27.4, 23.6)	N(32.8, 5)	N(13.2, 19.2)
91	N(-9.5, 5)	N(26.2, 16)	N(-15.8, 5)	N(29.2, 27.8)	N(-6.6, 5)	N(-33.9, 9.8)	N(19.1, 5)	N(3.8, 13.3)
92	N(-32.8, 5)	N(-13.2, 19.2)	N(-4.5, 5)	N(8.3, 9.5)	N(5.2, 5)	N(0, 11.1)	N(15.3, 5)	N(-4, 23.6)
93	N(-3.2, 5)	N(-1.5, 27.4)	N(-29, 5)	N(4, 14.9)	N(14.1, 5)	N(-19.7, 28.9)	N(9.4, 5)	N(6.6, 20)
94	N(4, 5)	N(18.3, 21.4)	N(-11.7, 5)	N(17, 27.1)	N(14.1, 5)	N(-19.7, 28.9)	N(6.4, 5)	N(-14.8, 18.9)
95	N(-5.2, 5)	N(0, 11.1)	N(-24.5, 5)	N(-19.6, 14.2)	N(33.1, 5)	N(-35.7, 26.7)	N(11.3, 5)	N(6.3, 22.8)
96	N(2.7, 5)	N(23.6, 18.4)	N(-18.9, 5)	N(16, 26.9)	N(-16.3, 5)	N(-25.6, 25)	N(9.5, 5)	N(-26.2, 16)
97	N(18.1, 5)	N(19.7, 12)	N(13.7, 5)	N(21.3, 8.3)	N(19.4, 5)	N(-33.9, 19.4)	N(-4, 5)	N(-18.3, 21.4)
98	N(-24.5, 5)	N(-19.6, 14.2)	N(-15.8, 5)	N(29.2, 27.8)	N(-24.2, 5)	N(-26.6, 5.5)	N(33, 5)	N(11.9, 10.7)
99	N(-33, 5)	N(-11.9, 10.7)	N(-18.9, 5)	N(16, 26.9)	N(-2.7, 5)	N(-23.6, 18.4)	N(9.4, 5)	N(6.6, 20)
100	N(6.6, 5)	N(33.9, 9.8)	N(-10.3, 5)	N(15.9, 25.2)	N(-15.2, 5)	N(-31.2, 21.2)	N(-35, 5)	N(-49.7, 9.4)

	risk seeking/aversion = 3/1							
	task 1		task 2		task 3		task 4	
simulation	safe	risky	safe	risky	safe	risky	safe	risky
1	N(-36.2, 5)	N(-11.8, 26.2)	N(0, 5)	N(15, 19.6)	N(11.6, 5)	N(36.9, 9.5)	N(-18.5, 5)	N(-25.3, 22.6)
2	N(-16.6, 5)	N(6.2, 6.9)	N(-31, 5)	N(-18.8, 24.4)	N(11.6, 5)	N(36.9, 9.5)	N(-6.7, 5)	N(-23.6, 20)
3	N(-10.1, 5)	N(5.4, 11.5)	N(-16.9, 5)	N(-3.9, 12.9)	N(-23.8, 5)	N(-18.7, 7.1)	N(23.8, 5)	N(18.7, 7.1)
4	N(-15.5, 5)	N(-9.3, 18.3)	N(10.2, 5)	N(19.2, 10.3)	N(-16.9, 5)	N(-3.9, 12.9)	N(-11.6, 5)	N(-36.9, 9.5)
5	N(-14.9, 5)	N(-7.2, 25.2)	N(-21.8, 5)	N(4.5, 16.6)	N(-16.6, 5)	N(6.2, 6.9)	N(-2.4, 5)	N(-12.1, 23.7)
6	N(10.2, 5)	N(19.2, 10.3)	N(-15.5, 5)	N(-9.3, 18.3)	N(10.6, 5)	N(13, 24.8)	N(26, 5)	N(4.5, 18.3)
7	N(-21, 5)	N(5.3, 8.4)	N(-0.8, 5)	N(7, 16.7)	N(-10.1, 5)	N(6.8, 6.8)	N(31.2, 5)	N(23.3, 25.3)
8	N(24.7, 5)	N(55.2, 11.7)	N(-10.1, 5)	N(5.4, 11.5)	N(-4.9, 5)	N(24, 9.8)	N(21, 5)	N(-5.3, 8.4)
9	N(-8.3, 5)	N(13.3, 22.6)	N(-10.1, 5)	N(6.8, 6.8)	N(-10.4, 5)	N(22.3, 12.7)	N(16.9, 5)	N(3.9, 12.9)
10	N(-14.9, 5)	N(-7.2, 25.2)	N(-4.9, 5)	N(24, 9.8)	N(-8.2, 5)	N(-5.3, 25.7)	N(-16.8, 5)	N(-23, 15.8)
11	N(11.6, 5)	N(36.9, 9.5)	N(4.2, 5)	N(12.3, 7.3)	N(1.5, 5)	N(19.6, 24.9)	N(15.5, 5)	N(9.3, 18.3)
12	N(0, 5)	N(15, 19.6)	N(-21.1, 5)	N(12.5, 22.8)	N(-15.5, 5)	N(-9.3, 18.3)	N(3.9, 5)	N(-13.6, 24.8)
13	N(-15.8, 5)	N(-10.3, 16.7)	N(10.6, 5)	N(13, 24.8)	N(-3.9, 5)	N(13.6, 24.8)	N(10.1, 5)	N(-5.4, 11.5)
14	N(-8.3, 5)	N(13.3, 22.6)	N(1.5, 5)	N(19.6, 24.9)	N(-21.8, 5)	N(4.5, 16.6)	N(11.1, 5)	N(3.3, 23.1)
15	N(10.2, 5)	N(19.2, 10.3)	N(24.7, 5)	N(55.2, 11.7)	N(4.1, 5)	N(4.5, 27.3)	N(8.2, 5)	N(5.3, 25.7)
16	N(18.5, 5)	N(25.3, 22.6)	N(-15.8, 5)	N(-10.3, 16.7)	N(-17.4, 5)	N(48.8, 29.5)	N(-28.8, 5)	N(-32.2, 16.8)
17	N(-30.1, 5)	N(32.9, 18)	N(-0.8, 5)	N(7, 16.7)	N(-21.8, 5)	N(4.5, 16.6)	N(22.4, 5)	N(11.2, 29.2)
18	N(-8.3, 5)	N(13.3, 22.6)	N(-11.1, 5)	N(-3.3, 23.1)	N(2.4, 5)	N(12.1, 23.7)	N(31.2, 5)	N(23.3, 25.3)
19	N(-19.6, 5)	N(3.1, 12.2)	N(-11.1, 5)	N(-3.3, 23.1)	N(4.2, 5)	N(12.3, 7.3)	N(10.1, 5)	N(-6.8, 6.8)
20	N(-36.2, 5)	N(-11.8, 26.2)	N(-11.1, 5)	N(-3.3, 23.1)	N(24.7, 5)	N(55.2, 11.7)	N(15.8, 5)	N(10.3, 16.7)
21	N(-31.2, 5)	N(-23.3, 25.3)	N(2.4, 5)	N(12.1, 23.7)	N(-0.3, 5)	N(23.8, 11.8)	N(23.8, 5)	N(18.7, 7.1)
22	N(-26, 5)	N(-4.5, 18.3)	N(-10.4, 5)	N(22.3, 12.7)	N(-17.4, 5)	N(48.8, 29.5)	N(11.1, 5)	N(3.3, 23.1)
23	N(-21.1, 5)	N(12.5, 22.8)	N(-20.4, 5)	N(1.2, 25.9)	N(-19.6, 5)	N(3.1, 12.2)	N(-1.5, 5)	N(-19.6, 24.9)
24	N(4.1, 5)	N(4.5, 27.3)	N(-22.4, 5)	N(-11.2, 29.2)	N(18.5, 5)	N(25.3, 22.6)	N(-24.7, 5)	N(-55.2, 11.7)
25	N(-23.2, 5)	N(-9, 20.8)	N(-15.5, 5)	N(-9.3, 18.3)	N(4.2, 5)	N(12.3, 7.3)	N(-2.4, 5)	N(-12.1, 23.7)
26	N(-31, 5)	N(-18.8, 24.4)	N(18.5, 5)	N(25.3, 22.6)	N(16.8, 5)	N(23, 15.8)	N(-16.8, 5)	N(-23, 15.8)
27	N(-15.8, 5)	N(-10.3, 16.7)	N(-28, 5)	N(47, 29.1)	N(4.1, 5)	N(4.5, 27.3)	N(8.3, 5)	N(-13.3, 22.6)
28	N(10.6, 5)	N(13, 24.8)	N(-21.8, 5)	N(4.5, 16.6)	N(6.7, 5)	N(23.6, 20)	N(21.1, 5)	N(-12.5, 22.8)
29	N(-0.3, 5)	N(23.8, 11.8)	N(0.8, 5)	N(1.5, 7.2)	N(28.8, 5)	N(32.2, 16.8)	N(21, 5)	N(-5.3, 8.4)
30	N(-28, 5)	N(47, 29.1)	N(-13.6, 5)	N(12.3, 14.9)	N(18.5, 5)	N(25.3, 22.6)	N(16.6, 5)	N(-6.2, 6.9)
31	N(-16.9, 5)	N(-3.9, 12.9)	N(-10.1, 5)	N(5.4, 11.5)	N(-0.3, 5)	N(23.8, 11.8)	N(0.3, 5)	N(-23.8, 11.8)
32	N(-15.5, 5)	N(-9.3, 18.3)	N(-3.9, 5)	N(13.6, 24.8)	N(-21.1, 5)	N(12.5, 22.8)	N(0, 5)	N(-15, 19.6)
33	N(-31, 5)	N(-18.8, 24.4)	N(10.2, 5)	N(19.2, 10.3)	N(11.6, 5)	N(36.9, 9.5)	N(10.4, 5)	N(-22.3, 12.7)
34	N(-8.3, 5)	N(13.3, 22.6)	N(-17.4, 5)	N(48.8, 29.5)	N(2.4, 5)	N(12.1, 23.7)	N(14.9, 5)	N(7.2, 25.2)
35	N(11.6, 5)	N(36.9, 9.5)	N(1.5, 5)	N(19.6, 24.9)	N(-10.1, 5)	N(6.8, 6.8)	N(-10.6, 5)	N(-13, 24.8)
36	N(-21, 5)	N(5.3, 8.4)	N(-16.6, 5)	N(6.2, 6.9)	N(6.7, 5)	N(23.6, 20)	N(28, 5)	N(-47, 29.1)
37	N(-21, 5)	N(5.3, 8.4)	N(-8.3, 5)	N(13.3, 22.6)	N(4.2, 5)	N(12.3, 7.3)	N(31.2, 5)	N(23.3, 25.3)
38	N(-15.5, 5)	N(-9.3, 18.3)	N(-22.4, 5)	N(-11.2, 29.2)	N(28.8, 5)	N(32.2, 16.8)	N(10.4, 5)	N(-22.3, 12.7)
39	N(-31, 5)	N(-18.8, 24.4)	N(0, 5)	N(15, 19.6)	N(-21.8, 5)	N(4.5, 16.6)	N(21.1, 5)	N(-12.5, 22.8)
40	N(-14.9, 5)	N(-7.2, 25.2)	N(-4.9, 5)	N(24, 9.8)	N(-16.9, 5)	N(-3.9, 12.9)	N(-10.2, 5)	N(-19.2, 10.3)
41	N(-0.3, 5)	N(23.8, 11.8)	N(24.7, 5)	N(55.2, 11.7)	N(-21, 5)	N(5.3, 8.4)	N(-18.5, 5)	N(-25.3, 22.6)
42	N(7.6, 5)	N(21.2, 21.1)	N(-23.1, 5)	N(13.3, 13.9)	N(-3.9, 5)	N(13.6, 24.8)	N(36.2, 5)	N(11.8, 26.2)
43	N(-0.8, 5)	N(7, 16.7)	N(10.2, 5)	N(19.2, 10.3)	N(-15.5, 5)	N(-9.3, 18.3)	N(-10.6, 5)	N(-13, 24.8)
44	N(-10.1, 5)	N(5.4, 11.5)	N(-13.6, 5)	N(12.3, 14.9)	N(2.4, 5)	N(12.1, 23.7)	N(22.4, 5)	N(11.2, 29.2)
45	N(1.2, 5)	N(9, 19.6)	N(-30.1, 5)	N(32.9, 18)	N(-23.1, 5)	N(13.3, 13.9)	N(36.2, 5)	N(11.8, 26.2)
46	N(-3.9, 5)	N(13.6, 24.8)	N(-19.6, 5)	N(3.1, 12.2)	N(-8.3, 5)	N(13.3, 22.6)	N(23.8, 5)	N(18.7, 7.1)
47	N(-31.2, 5)	N(-23.3, 25.3)	N(-20.4, 5)	N(1.2, 25.9)	N(18.5, 5)	N(25.3, 22.6)	N(-7.6, 5)	N(-21.2, 21.1)
48	N(-10.1, 5)	N(5.4, 11.5)	N(0, 5)	N(15, 19.6)	N(-28, 5)	N(47, 29.1)	N(0.3, 5)	N(-23.8, 11.8)
49	N(-4.9, 5)	N(24, 9.8)	N(18.5, 5)	N(25.3, 22.6)	N(-8.2, 5)	N(-5.3, 25.7)	N(16.6, 5)	N(-6.2, 6.9)
50	N(10.6, 5)	N(13, 24.8)	N(-0.8, 5)	N(7, 16.7)	N(0, 5)	N(15, 19.6)	N(-0.8, 5)	N(-1.5, 7.2)

51	N(1.5, 5)	N(19.6, 24.9)	N(-18.9, 5)	N(8.5, 29.2)	N(-3.9, 5)	N(13.6, 24.8)	N(18.9, 5)	N(-8.5, 29.2)
52	N(16.8, 5)	N(23, 15.8)	N(-10.4, 5)	N(22.3, 12.7)	N(0.8, 5)	N(1.5, 7.2)	N(-28.8, 5)	N(-32.2, 16.8)
53	N(-8.2, 5)	N(-5.3, 25.7)	N(-21, 5)	N(5.3, 8.4)	N(-17.4, 5)	N(48.8, 29.5)	N(21.8, 5)	N(-4.5, 16.6)
54	N(-8.3, 5)	N(13.3, 22.6)	N(-15.8, 5)	N(-10.3, 16.7)	N(-13.6, 5)	N(12.3, 14.9)	N(20.4, 5)	N(-1.2, 25.9)
55	N(-21.8, 5)	N(4.5, 16.6)	N(2.2, 5)	N(9.7, 17.8)	N(10.6, 5)	N(13, 24.8)	N(15.5, 5)	N(9.3, 18.3)
56	N(-11.1, 5)	N(-3.3, 23.1)	N(-19.6, 5)	N(3.1, 12.2)	N(2.2, 5)	N(9.7, 17.8)	N(0.8, 5)	N(-7, 16.7)
57	N(-16.9, 5)	N(-3.9, 12.9)	N(10.6, 5)	N(13, 24.8)	N(4.1, 5)	N(4.5, 27.3)	N(16.9, 5)	N(3.9, 12.9)
58	N(-11.1, 5)	N(-3.3, 23.1)	N(-17.4, 5)	N(48.8, 29.5)	N(10.2, 5)	N(19.2, 10.3)	N(3.9, 5)	N(-13.6, 24.8)
59	N(2.2, 5)	N(9.7, 17.8)	N(-11.1, 5)	N(-3.3, 23.1)	N(-3.9, 5)	N(13.6, 24.8)	N(22.4, 5)	N(11.2, 29.2)
60	N(-17.4, 5)	N(48.8, 29.5)	N(-23.1, 5)	N(13.3, 13.9)	N(24.7, 5)	N(55.2, 11.7)	N(10.1, 5)	N(-5.4, 11.5)
61	N(16.8, 5)	N(23, 15.8)	N(-0.8, 5)	N(7, 16.7)	N(-26, 5)	N(-4.5, 18.3)	N(-11.6, 5)	N(-36.9, 9.5)
62	N(-21.8, 5)	N(4.5, 16.6)	N(-15.5, 5)	N(-9.3, 18.3)	N(-10.1, 5)	N(5.4, 11.5)	N(30.1, 5)	N(-32.9, 18)
63	N(-10.1, 5)	N(5.4, 11.5)	N(-0.3, 5)	N(23.8, 11.8)	N(-30.1, 5)	N(32.9, 18)	N(15.5, 5)	N(9.3, 18.3)
64	N(6.7, 5)	N(23.6, 20)	N(11.6, 5)	N(36.9, 9.5)	N(-10.4, 5)	N(22.3, 12.7)	N(23.8, 5)	N(18.7, 7.1)
65	N(-11.1, 5)	N(-3.3, 23.1)	N(-16.9, 5)	N(-3.9, 12.9)	N(-21, 5)	N(5.3, 8.4)	N(-10.6, 5)	N(-13, 24.8)
66	N(-14.9, 5)	N(-7.2, 25.2)	N(-19.6, 5)	N(3.1, 12.2)	N(-21.1, 5)	N(12.5, 22.8)	N(0, 5)	N(-15, 19.6)
67	N(-16.6, 5)	N(6.2, 6.9)	N(-8.2, 5)	N(-5.3, 25.7)	N(1.2, 5)	N(9, 19.6)	N(21, 5)	N(-5.3, 8.4)
68	N(-31.2, 5)	N(-23.3, 25.3)	N(-21.8, 5)	N(4.5, 16.6)	N(7.6, 5)	N(21.2, 21.1)	N(15.5, 5)	N(9.3, 18.3)
69	N(-0.3, 5)	N(23.8, 11.8)	N(-0.8, 5)	N(7, 16.7)	N(24.7, 5)	N(55.2, 11.7)	N(-18.5, 5)	N(-25.3, 22.6)
70	N(-0.3, 5)	N(23.8, 11.8)	N(-4.9, 5)	N(24, 9.8)	N(4.2, 5)	N(12.3, 7.3)	N(10.1, 5)	N(-5.4, 11.5)
71	N(-8.2, 5)	N(-5.3, 25.7)	N(6.7, 5)	N(23.6, 20)	N(18.5, 5)	N(25.3, 22.6)	N(19.6, 5)	N(-3.1, 12.2)
72	N(2.2, 5)	N(9.7, 17.8)	N(-8.3, 5)	N(13.3, 22.6)	N(-4.9, 5)	N(24, 9.8)	N(-1.5, 5)	N(-19.6, 24.9)
73	N(-10.1, 5)	N(6.8, 6.8)	N(10.2, 5)	N(19.2, 10.3)	N(-15.5, 5)	N(-9.3, 18.3)	N(10.1, 5)	N(-6.8, 6.8)
74	N(16.8, 5)	N(23, 15.8)	N(-8.2, 5)	N(-5.3, 25.7)	N(28.8, 5)	N(32.2, 16.8)	N(19.6, 5)	N(-3.1, 12.2)
75	N(-16.9, 5)	N(-3.9, 12.9)	N(10.6, 5)	N(13, 24.8)	N(-21.1, 5)	N(12.5, 22.8)	N(0.8, 5)	N(-7, 16.7)
76	N(-0.8, 5)	N(7, 16.7)	N(-28, 5)	N(47, 29.1)	N(-31.2, 5)	N(-23.3, 25.3)	N(10.4, 5)	N(-22.3, 12.7)
77	N(-31, 5)	N(-18.8, 24.4)	N(16.8, 5)	N(23, 15.8)	N(0.8, 5)	N(1.5, 7.2)	N(23.8, 5)	N(18.7, 7.1)
78	N(4.2, 5)	N(12.3, 7.3)	N(-10.4, 5)	N(22.3, 12.7)	N(-8.2, 5)	N(-5.3, 25.7)	N(17.4, 5)	N(-48.8, 29.5)
79	N(-18.9, 5)	N(8.5, 29.2)	N(-36.2, 5)	N(-11.8, 26.2)	N(-21.8, 5)	N(4.5, 16.6)	N(0.8, 5)	N(-7, 16.7)
80	N(-22.4, 5)	N(-11.2, 29.2)	N(28.8, 5)	N(32.2, 16.8)	N(2.2, 5)	N(9.7, 17.8)	N(26, 5)	N(4.5, 18.3)
81	N(0, 5)	N(15, 19.6)	N(-10.1, 5)	N(6.8, 6.8)	N(-15.5, 5)	N(-9.3, 18.3)	N(-1.5, 5)	N(-19.6, 24.9)
82	N(-21.8, 5)	N(4.5, 16.6)	N(18.5, 5)	N(25.3, 22.6)	N(-19.6, 5)	N(3.1, 12.2)	N(19.6, 5)	N(-3.1, 12.2)
83	N(-0.3, 5)	N(23.8, 11.8)	N(-10.1, 5)	N(6.8, 6.8)	N(-3.9, 5)	N(13.6, 24.8)	N(3.9, 5)	N(-13.6, 24.8)
84	N(4.2, 5)	N(12.3, 7.3)	N(-8.2, 5)	N(-5.3, 25.7)	N(2.4, 5)	N(12.1, 23.7)	N(16.9, 5)	N(3.9, 12.9)
85	N(2.4, 5)	N(12.1, 23.7)	N(6.7, 5)	N(23.6, 20)	N(10.6, 5)	N(13, 24.8)	N(-24.7, 5)	N(-55.2, 11.7)
86	N(-36.2, 5)	N(-11.8, 26.2)	N(1.5, 5)	N(19.6, 24.9)	N(10.6, 5)	N(13, 24.8)	N(23.8, 5)	N(18.7, 7.1)
87	N(-0.8, 5)	N(7, 16.7)	N(-8.2, 5)	N(-5.3, 25.7)	N(-23.1, 5)	N(13.3, 13.9)	N(-6.7, 5)	N(-23.6, 20)
88	N(-3.9, 5)	N(13.6, 24.8)	N(-21.1, 5)	N(12.5, 22.8)	N(-0.3, 5)	N(23.8, 11.8)	N(31, 5)	N(18.8, 24.4)
89	N(-15.5, 5)	N(-9.3, 18.3)	N(2.2, 5)	N(9.7, 17.8)	N(-21.8, 5)	N(4.5, 16.6)	N(-1.5, 5)	N(-19.6, 24.9)
90	N(-13.6, 5)	N(12.3, 14.9)	N(-10.4, 5)	N(22.3, 12.7)	N(7.6, 5)	N(21.2, 21.1)	N(15.8, 5)	N(10.3, 16.7)
91	N(-23.2, 5)	N(-9, 20.8)	N(-20.4, 5)	N(1.2, 25.9)	N(-26, 5)	N(-4.5, 18.3)	N(0.3, 5)	N(-23.8, 11.8)
92	N(-16.9, 5)	N(-3.9, 12.9)	N(-10.4, 5)	N(22.3, 12.7)	N(-23.1, 5)	N(13.3, 13.9)	N(-4.2, 5)	N(-12.3, 7.3)
93	N(28.8, 5)	N(32.2, 16.8)	N(-30.1, 5)	N(32.9, 18)	N(-10.1, 5)	N(5.4, 11.5)	N(26, 5)	N(4.5, 18.3)
94	N(-15.8, 5)	N(-10.3, 16.7)	N(1.2, 5)	N(9, 19.6)	N(-0.8, 5)	N(7, 16.7)	N(21.1, 5)	N(-12.5, 22.8)
95	N(11.6, 5)	N(36.9, 9.5)	N(-15.8, 5)	N(-10.3, 16.7)	N(-22.4, 5)	N(-11.2, 29.2)	N(15.8, 5)	N(10.3, 16.7)
96	N(11.6, 5)	N(36.9, 9.5)	N(-16.9, 5)	N(-3.9, 12.9)	N(2.2, 5)	N(9.7, 17.8)	N(3.9, 5)	N(-13.6, 24.8)
97	N(-16.6, 5)	N(6.2, 6.9)	N(-10.1, 5)	N(5.4, 11.5)	N(1.2, 5)	N(9, 19.6)	N(21, 5)	N(-5.3, 8.4)
98	N(16.8, 5)	N(23, 15.8)	N(2.2, 5)	N(9.7, 17.8)	N(-20.4, 5)	N(1.2, 25.9)	N(31.2, 5)	N(23.3, 25.3)
99	N(24.7, 5)	N(55.2, 11.7)	N(-21.1, 5)	N(12.5, 22.8)	N(-21.8, 5)	N(4.5, 16.6)	N(10.1, 5)	N(-6.8, 6.8)
100	N(6.7, 5)	N(23.6, 20)	N(28.8, 5)	N(32.2, 16.8)	N(-23.1, 5)	N(13.3, 13.9)	N(19.6, 5)	N(-3.1, 12.2)

risk seeking/aversion = 4/0								
	task 1		task 2		task 3		task 4	
simulation	safe	risky	safe	risky	safe	risky	safe	risky
1	N(16.2, 5)	N(25.5, 9.3)	N(-26.4, 5)	N(-10.2, 11.6)	N(-25.2, 5)	N(48.4, 27.2)	N(-8.2, 5)	N(27, 20.4)
2	N(-25.2, 5)	N(48.4, 27.2)	N(-7, 5)	N(7.5, 12.5)	N(10.5, 5)	N(23.5, 29.4)	N(-1, 5)	N(24, 12.2)
3	N(6.8, 5)	N(26.1, 24.4)	N(-11.3, 5)	N(5.1, 16.3)	N(-33.5, 5)	N(14.4, 17.7)	N(-7.4, 5)	N(5.8, 11.4)
4	N(7.4, 5)	N(18.2, 22.9)	N(-9.4, 5)	N(17.3, 5.8)	N(-22.1, 5)	N(-19.4, 28.4)	N(-8.2, 5)	N(27, 20.4)
5	N(16.2, 5)	N(25.5, 9.3)	N(1.3, 5)	N(1.4, 27.9)	N(-1.8, 5)	N(17.1, 5.4)	N(-12.3, 5)	N(6.9, 23.4)
6	N(4.8, 5)	N(17.8, 9.1)	N(-9.4, 5)	N(17.3, 5.8)	N(6.7, 5)	N(33.9, 17)	N(1.3, 5)	N(1.4, 27.9)
7	N(-18.3, 5)	N(-13, 17.6)	N(0.3, 5)	N(4.7, 9.6)	N(-1, 5)	N(34, 19.6)	N(16.2, 5)	N(25.5, 9.3)
8	N(-24.2, 5)	N(-8.3, 5.5)	N(-14.8, 5)	N(-7.8, 5.9)	N(-14, 5)	N(7.3, 9.6)	N(-31.4, 5)	N(-22.9, 8.1)
9	N(-14, 5)	N(7.3, 9.6)	N(-22.1, 5)	N(-19.4, 28.4)	N(-0.5, 5)	N(4.2, 18.9)	N(-26.4, 5)	N(-10.2, 11.6)
10	N(-24.2, 5)	N(-8.3, 5.5)	N(6.8, 5)	N(26.1, 24.4)	N(-18.3, 5)	N(-13, 17.6)	N(0.9, 5)	N(8.3, 29.8)
11	N(-24.2, 5)	N(-8.3, 5.5)	N(-16.2, 5)	N(-15.2, 22)	N(-25.2, 5)	N(48.4, 27.2)	N(-31.9, 5)	N(29.8, 23.6)
12	N(8.9, 5)	N(20.1, 6)	N(-14.8, 5)	N(-7.8, 5.9)	N(-5.6, 5)	N(18.8, 5)	N(1.3, 5)	N(1.4, 27.9)
13	N(-11.1, 5)	N(14.3, 8.3)	N(0.3, 5)	N(4.7, 9.6)	N(-5.9, 5)	N(15.9, 29.8)	N(10.5, 5)	N(23.5, 29.4)
14	N(-22.1, 5)	N(-19.4, 28.4)	N(18.1, 5)	N(29.7, 18.6)	N(-59.2, 5)	N(-48.3, 7.8)	N(-1, 5)	N(34, 19.6)
15	N(4.2, 5)	N(23.9, 24.7)	N(6.7, 5)	N(33.9, 17)	N(-12.3, 5)	N(6.9, 23.4)	N(6.5, 5)	N(8.2, 5)
16	N(4.8, 5)	N(17.8, 9.1)	N(18.1, 5)	N(29.7, 18.6)	N(13.8, 5)	N(29.6, 16.7)	N(12.8, 5)	N(36.4, 6.3)
17	N(4.2, 5)	N(23.9, 24.7)	N(10.5, 5)	N(23.5, 29.4)	N(6.7, 5)	N(33.9, 17)	N(13.8, 5)	N(29.6, 16.7)
18	N(-33.9, 5)	N(9.2, 21.7)	N(-1, 5)	N(34, 19.6)	N(-33.5, 5)	N(14.4, 17.7)	N(-18.3, 5)	N(-13, 17.6)
19	N(-9.4, 5)	N(17.3, 5.8)	N(12.8, 5)	N(36.4, 6.3)	N(26.9, 5)	N(37.7, 19.2)	N(7.4, 5)	N(18.2, 22.9)
20	N(-14, 5)	N(7.3, 9.6)	N(-14.8, 5)	N(-7.8, 5.9)	N(18.1, 5)	N(29.7, 18.6)	N(35.9, 5)	N(40.7, 25.4)
21	N(-16.2, 5)	N(-15.2, 22)	N(-25.2, 5)	N(48.4, 27.2)	N(18.1, 5)	N(29.7, 18.6)	N(35.9, 5)	N(40.7, 25.4)
22	N(-59.2, 5)	N(-48.3, 7.8)	N(-22.1, 5)	N(-19.4, 28.4)	N(-5.6, 5)	N(18.8, 5)	N(-7.4, 5)	N(5.8, 11.4)
23	N(7.4, 5)	N(18.2, 22.9)	N(6.7, 5)	N(33.9, 17)	N(-11.1, 5)	N(14.3, 8.3)	N(-31.4, 5)	N(-22.9, 8.1)
24	N(-59.2, 5)	N(-48.3, 7.8)	N(-7.4, 5)	N(5.8, 11.4)	N(-5.6, 5)	N(18.8, 5)	N(-1, 5)	N(24, 12.2)
25	N(1.3, 5)	N(1.4, 27.9)	N(-12.3, 5)	N(6.9, 23.4)	N(-11.9, 5)	N(-10.6, 8.1)	N(-26.4, 5)	N(-10.2, 11.6)
26	N(12.8, 5)	N(36.4, 6.3)	N(16.2, 5)	N(25.5, 9.3)	N(-14, 5)	N(7.3, 9.6)	N(-40.5, 5)	N(-7.5, 24.1)
27	N(-1, 5)	N(24, 12.2)	N(0.9, 5)	N(8.3, 29.8)	N(1.3, 5)	N(1.4, 27.9)	N(-14, 5)	N(7.3, 9.6)
28	N(8.9, 5)	N(20.1, 6)	N(-22.1, 5)	N(-19.4, 28.4)	N(2.1, 5)	N(23.4, 13.5)	N(-31.9, 5)	N(29.8, 23.6)
29	N(10.5, 5)	N(23.5, 29.4)	N(-1, 5)	N(24, 12.2)	N(-7.4, 5)	N(5.8, 11.4)	N(0.3, 5)	N(4.7, 9.6)
30	N(-11.9, 5)	N(-3.2, 24.5)	N(-33.5, 5)	N(14.4, 17.7)	N(-16.2, 5)	N(-15.2, 22)	N(6.5, 5)	N(8.2, 5)
31	N(-26.4, 5)	N(-10.2, 11.6)	N(-1, 5)	N(24, 12.2)	N(-11.3, 5)	N(5.1, 16.3)	N(26.9, 5)	N(37.7, 19.2)
32	N(-12.3, 5)	N(6.9, 23.4)	N(-31.4, 5)	N(-22.9, 8.1)	N(4.8, 5)	N(17.8, 9.1)	N(-11.9, 5)	N(-3.2, 24.5)
33	N(-11.3, 5)	N(5.1, 16.3)	N(-1, 5)	N(24, 12.2)	N(-40.5, 5)	N(-7.5, 24.1)	N(-2, 5)	N(30.4, 13.2)
34	N(1.3, 5)	N(1.4, 27.9)	N(-9.4, 5)	N(17.3, 5.8)	N(-7.4, 5)	N(5.8, 11.4)	N(-31.4, 5)	N(-22.9, 8.1)
35	N(6.5, 5)	N(8.2, 5)	N(13.8, 5)	N(29.6, 16.7)	N(-1.8, 5)	N(17.1, 5.4)	N(-9.4, 5)	N(17.3, 5.8)
36	N(12.8, 5)	N(36.4, 6.3)	N(-16.2, 5)	N(-15.2, 22)	N(10.5, 5)	N(23.5, 29.4)	N(-1, 5)	N(34, 19.6)
37	N(-31.4, 5)	N(-22.9, 8.1)	N(6.5, 5)	N(8.2, 5)	N(4.2, 5)	N(23.9, 24.7)	N(-18.3, 5)	N(-13, 17.6)
38	N(0.3, 5)	N(4.7, 9.6)	N(-31.4, 5)	N(-22.9, 8.1)	N(-11.3, 5)	N(5.1, 16.3)	N(35.9, 5)	N(40.7, 25.4)
39	N(-9.4, 5)	N(17.3, 5.8)	N(-11.9, 5)	N(-10.6, 8.1)	N(35.9, 5)	N(40.7, 25.4)	N(2.1, 5)	N(23.4, 13.5)
40	N(-40.5, 5)	N(-7.5, 24.1)	N(-25.2, 5)	N(48.4, 27.2)	N(2.1, 5)	N(23.4, 13.5)	N(35.9, 5)	N(40.7, 25.4)
41	N(-11.9, 5)	N(-10.6, 8.1)	N(-5.9, 5)	N(15.9, 29.8)	N(-11.3, 5)	N(5.1, 16.3)	N(4.2, 5)	N(23.9, 24.7)
42	N(8.9, 5)	N(20.1, 6)	N(-11.9, 5)	N(-10.6, 8.1)	N(-16.2, 5)	N(-15.2, 22)	N(6.5, 5)	N(8.2, 5)
43	N(4.8, 5)	N(17.8, 9.1)	N(-33.5, 5)	N(14.4, 17.7)	N(6.7, 5)	N(33.9, 17)	N(-8.2, 5)	N(27, 20.4)
44	N(-12.3, 5)	N(6.9, 23.4)	N(-7.4, 5)	N(5.8, 11.4)	N(18.1, 5)	N(29.7, 18.6)	N(6.5, 5)	N(8.2, 5)
45	N(13.6, 5)	N(14.2, 28.6)	N(-14.8, 5)	N(-7.8, 5.9)	N(-22.1, 5)	N(-19.4, 28.4)	N(-33.5, 5)	N(14.4, 17.7)
46	N(6.8, 5)	N(26.1, 24.4)	N(0.9, 5)	N(8.3, 29.8)	N(4.8, 5)	N(17.8, 9.1)	N(-1.8, 5)	N(17.1, 5.4)
47	N(-33.9, 5)	N(9.2, 21.7)	N(-0.5, 5)	N(4.2, 18.9)	N(12.8, 5)	N(36.4, 6.3)	N(4.2, 5)	N(23.9, 24.7)
48	N(-31.4, 5)	N(-22.9, 8.1)	N(35.9, 5)	N(40.7, 25.4)	N(0.9, 5)	N(8.3, 29.8)	N(26.9, 5)	N(37.7, 19.2)
49	N(6.8, 5)	N(26.1, 24.4)	N(-12.3, 5)	N(6.9, 23.4)	N(-33.9, 5)	N(9.2, 21.7)	N(-25.2, 5)	N(48.4, 27.2)
50	N(4.2, 5)	N(23.9, 24.7)	N(0.3, 5)	N(4.7, 9.6)	N(-33.5, 5)	N(14.4, 17.7)	N(-8.2, 5)	N(27, 20.4)

51	N(-11.4, 5)	N(39.6, 27.3)	N(-14.8, 5)	N(-7.8, 5.9)	N(0.9, 5)	N(8.3, 29.8)	N(-11.9, 5)	N(-10.6, 8.1)
52	N(6.7, 5)	N(33.9, 17)	N(-11.9, 5)	N(-3.2, 24.5)	N(26.9, 5)	N(37.7, 19.2)	N(13.8, 5)	N(29.6, 16.7)
53	N(1.3, 5)	N(1.4, 27.9)	N(-2, 5)	N(30.4, 13.2)	N(13.6, 5)	N(14.2, 28.6)	N(-9.4, 5)	N(17.3, 5.8)
54	N(-7, 5)	N(7.5, 12.5)	N(18.1, 5)	N(29.7, 18.6)	N(35.9, 5)	N(40.7, 25.4)	N(-12.3, 5)	N(6.9, 23.4)
55	N(-14.8, 5)	N(-7.8, 5.9)	N(13.6, 5)	N(14.2, 28.6)	N(-11.9, 5)	N(-3.2, 24.5)	N(-1.8, 5)	N(17.1, 5.4)
56	N(2.1, 5)	N(23.4, 13.5)	N(13.6, 5)	N(14.2, 28.6)	N(-1, 5)	N(24, 12.2)	N(-26.4, 5)	N(-10.2, 11.6)
57	N(-1.8, 5)	N(17.1, 5.4)	N(26.9, 5)	N(37.7, 19.2)	N(7.4, 5)	N(18.2, 22.9)	N(-9.4, 5)	N(17.3, 5.8)
58	N(7.4, 5)	N(18.2, 22.9)	N(-11.9, 5)	N(-3.2, 24.5)	N(-1, 5)	N(34, 19.6)	N(6.8, 5)	N(26.1, 24.4)
59	N(4.8, 5)	N(17.8, 9.1)	N(12.8, 5)	N(36.4, 6.3)	N(10.5, 5)	N(23.5, 29.4)	N(-33.9, 5)	N(9.2, 21.7)
60	N(0.3, 5)	N(4.7, 9.6)	N(7.4, 5)	N(18.2, 22.9)	N(0.9, 5)	N(8.3, 29.8)	N(2.1, 5)	N(23.4, 13.5)
61	N(-24.2, 5)	N(-8.3, 5.5)	N(10.5, 5)	N(23.5, 29.4)	N(-31.4, 5)	N(-22.9, 8.1)	N(-0.5, 5)	N(4.2, 18.9)
62	N(-31.4, 5)	N(-22.9, 8.1)	N(-7, 5)	N(7.5, 12.5)	N(6.8, 5)	N(26.1, 24.4)	N(7.4, 5)	N(18.2, 22.9)
63	N(16.2, 5)	N(25.5, 9.3)	N(-11.3, 5)	N(5.1, 16.3)	N(-18.3, 5)	N(-13, 17.6)	N(26.9, 5)	N(37.7, 19.2)
64	N(13.6, 5)	N(14.2, 28.6)	N(-14, 5)	N(7.3, 9.6)	N(-11.9, 5)	N(-10.6, 8.1)	N(-1.8, 5)	N(17.1, 5.4)
65	N(26.9, 5)	N(37.7, 19.2)	N(0.3, 5)	N(4.7, 9.6)	N(8.9, 5)	N(20.1, 6)	N(-31.9, 5)	N(29.8, 23.6)
66	N(16.2, 5)	N(25.5, 9.3)	N(2.1, 5)	N(23.4, 13.5)	N(6.7, 5)	N(33.9, 17)	N(6.5, 5)	N(8.2, 5)
67	N(-11.4, 5)	N(39.6, 27.3)	N(-2, 5)	N(30.4, 13.2)	N(-31.4, 5)	N(-22.9, 8.1)	N(6.8, 5)	N(26.1, 24.4)
68	N(-11.3, 5)	N(5.1, 16.3)	N(-5.6, 5)	N(18.8, 5)	N(0.9, 5)	N(8.3, 29.8)	N(-16.2, 5)	N(-15.2, 22)
69	N(7.4, 5)	N(18.2, 22.9)	N(-31.4, 5)	N(-22.9, 8.1)	N(-33.5, 5)	N(14.4, 17.7)	N(-11.9, 5)	N(-10.6, 8.1)
70	N(-40.5, 5)	N(-7.5, 24.1)	N(-11.1, 5)	N(14.3, 8.3)	N(6.5, 5)	N(8.2, 5)	N(-1, 5)	N(24, 12.2)
71	N(12.8, 5)	N(36.4, 6.3)	N(6.7, 5)	N(33.9, 17)	N(13.6, 5)	N(14.2, 28.6)	N(-22.1, 5)	N(-19.4, 28.4)
72	N(-12.3, 5)	N(6.9, 23.4)	N(-14, 5)	N(7.3, 9.6)	N(2.1, 5)	N(23.4, 13.5)	N(-9.4, 5)	N(17.3, 5.8)
73	N(-22.1, 5)	N(-19.4, 28.4)	N(10.5, 5)	N(23.5, 29.4)	N(-1.8, 5)	N(17.1, 5.4)	N(-59.2, 5)	N(-48.3, 7.8)
74	N(-11.9, 5)	N(-3.2, 24.5)	N(-8.2, 5)	N(27, 20.4)	N(13.6, 5)	N(14.2, 28.6)	N(-1, 5)	N(24, 12.2)
75	N(13.8, 5)	N(29.6, 16.7)	N(-1.8, 5)	N(17.1, 5.4)	N(-25.2, 5)	N(48.4, 27.2)	N(18.1, 5)	N(29.7, 18.6)
76	N(-31.4, 5)	N(-22.9, 8.1)	N(-11.4, 5)	N(39.6, 27.3)	N(6.8, 5)	N(26.1, 24.4)	N(-33.9, 5)	N(9.2, 21.7)
77	N(-1, 5)	N(34, 19.6)	N(2.1, 5)	N(23.4, 13.5)	N(-11.1, 5)	N(14.3, 8.3)	N(-11.9, 5)	N(-10.6, 8.1)
78	N(-22.1, 5)	N(-19.4, 28.4)	N(6.7, 5)	N(33.9, 17)	N(13.8, 5)	N(29.6, 16.7)	N(10.5, 5)	N(23.5, 29.4)
79	N(-31.4, 5)	N(-22.9, 8.1)	N(1.3, 5)	N(1.4, 27.9)	N(-22.1, 5)	N(-19.4, 28.4)	N(6.8, 5)	N(26.1, 24.4)
80	N(7.4, 5)	N(18.2, 22.9)	N(-0.5, 5)	N(4.2, 18.9)	N(6.8, 5)	N(26.1, 24.4)	N(-1, 5)	N(24, 12.2)
81	N(-7, 5)	N(7.5, 12.5)	N(-7.4, 5)	N(5.8, 11.4)	N(-11.9, 5)	N(-3.2, 24.5)	N(-5.6, 5)	N(18.8, 5)
82	N(-7, 5)	N(7.5, 12.5)	N(2.1, 5)	N(23.4, 13.5)	N(-59.2, 5)	N(-48.3, 7.8)	N(18.1, 5)	N(29.7, 18.6)
83	N(7.4, 5)	N(18.2, 22.9)	N(-8.2, 5)	N(27, 20.4)	N(-1.8, 5)	N(17.1, 5.4)	N(6.7, 5)	N(33.9, 17)
84	N(-5.6, 5)	N(18.8, 5)	N(-7, 5)	N(7.5, 12.5)	N(-11.9, 5)	N(-10.6, 8.1)	N(0.3, 5)	N(4.7, 9.6)
85	N(0.9, 5)	N(8.3, 29.8)	N(-5.9, 5)	N(15.9, 29.8)	N(13.8, 5)	N(29.6, 16.7)	N(26.9, 5)	N(37.7, 19.2)
86	N(6.8, 5)	N(26.1, 24.4)	N(35.9, 5)	N(40.7, 25.4)	N(6.5, 5)	N(8.2, 5)	N(12.8, 5)	N(36.4, 6.3)
87	N(12.8, 5)	N(36.4, 6.3)	N(-59.2, 5)	N(-48.3, 7.8)	N(4.8, 5)	N(17.8, 9.1)	N(-25.2, 5)	N(48.4, 27.2)
88	N(13.8, 5)	N(29.6, 16.7)	N(-24.2, 5)	N(-8.3, 5.5)	N(0.3, 5)	N(4.7, 9.6)	N(-11.1, 5)	N(14.3, 8.3)
89	N(-33.9, 5)	N(9.2, 21.7)	N(0.9, 5)	N(8.3, 29.8)	N(-59.2, 5)	N(-48.3, 7.8)	N(-5.6, 5)	N(18.8, 5)
90	N(-1, 5)	N(24, 12.2)	N(0.9, 5)	N(8.3, 29.8)	N(13.8, 5)	N(29.6, 16.7)	N(16.2, 5)	N(25.5, 9.3)
91	N(-31.9, 5)	N(29.8, 23.6)	N(-1.8, 5)	N(17.1, 5.4)	N(-11.3, 5)	N(5.1, 16.3)	N(-0.5, 5)	N(4.2, 18.9)
92	N(-24.2, 5)	N(-8.3, 5.5)	N(-5.6, 5)	N(18.8, 5)	N(6.5, 5)	N(8.2, 5)	N(-14.8, 5)	N(-7.8, 5.9)
93	N(-11.3, 5)	N(5.1, 16.3)	N(1.3, 5)	N(1.4, 27.9)	N(8.9, 5)	N(20.1, 6)	N(2.1, 5)	N(23.4, 13.5)
94	N(13.6, 5)	N(14.2, 28.6)	N(-5.6, 5)	N(18.8, 5)	N(-16.2, 5)	N(-15.2, 22)	N(-33.9, 5)	N(9.2, 21.7)
95	N(-33.5, 5)	N(14.4, 17.7)	N(-2, 5)	N(30.4, 13.2)	N(-40.5, 5)	N(-7.5, 24.1)	N(-0.5, 5)	N(4.2, 18.9)
96	N(0.9, 5)	N(8.3, 29.8)	N(-14.8, 5)	N(-7.8, 5.9)	N(2.1, 5)	N(23.4, 13.5)	N(1.3, 5)	N(1.4, 27.9)
97	N(-8.2, 5)	N(27, 20.4)	N(-18.3, 5)	N(-13, 17.6)	N(-14, 5)	N(7.3, 9.6)	N(-11.1, 5)	N(14.3, 8.3)
98	N(-24.2, 5)	N(-8.3, 5.5)	N(-7.4, 5)	N(5.8, 11.4)	N(-22.1, 5)	N(-19.4, 28.4)	N(-2, 5)	N(30.4, 13.2)
99	N(-1.8, 5)	N(17.1, 5.4)	N(0.3, 5)	N(4.7, 9.6)	N(-11.9, 5)	N(-3.2, 24.5)	N(-12.3, 5)	N(6.9, 23.4)
100	N(10.5, 5)	N(23.5, 29.4)	N(-18.3, 5)	N(-13, 17.6)	N(8.9, 5)	N(20.1, 6)	N(-25.2, 5)	N(48.4, 27.2)

Note. An option is denoted as $N(\mu_2, \sigma_2)$ for a safe option and $N(\mu_1, \sigma_1)$ for a risky-option.
