# Supplementary material for: Risk preference as an outcome of evolutionarily adaptive learning mechanisms: An evolutionary simulation under diverse risky environments
Source: PLoS One. 2024 Aug 1;19(8):e0307991. doi: 10.1371/journal.pone.0307991 (PMC11293680; doi:10.1371/journal.pone.0307991)
Supplement: S3 Table — (PDF) [file pone.0307991.s030.pdf]

S3 Table. Frequency of agents who showed more risk aversion in the gain domain than in the loss domain when the model used a single learning rate

condition	rate gain > loss in first generation	rate gain > loss in last generation
risk seeking/aversion = 0/4	0.41841	0.22026
risk seeking/aversion = 1/3	0.41580	0.43601
risk seeking/aversion = 2/2	0.41653	0.50326
risk seeking/aversion = 3/1	0.41899	0.52546
risk seeking/aversion = 4/0	0.41794	0.52296

Note. Values indicate the frequency of agents whose difference in the risk-aversion rate between the gain and loss domains was positive (that is, the rate of the positive area in S16 Fig.b and d). The difference in risk aversion was calculated by subtracting the mean rate of risk aversion across the two loss-domain tasks from the mean rate across the two gain-domain tasks for each agent. The second and third column indicates the values of the agents in the first generation and last generation, respectively.
