# Supplementary material for: Risk preference as an outcome of evolutionarily adaptive learning mechanisms: An evolutionary simulation under diverse risky environments
Source: PLoS One. 2024 Aug 1;19(8):e0307991. doi: 10.1371/journal.pone.0307991 (PMC11293680; doi:10.1371/journal.pone.0307991)
Supplement: S4 Table — (PDF) [file pone.0307991.s031.pdf]

S4 Table. Summary of statistics of Cohen's *d* in the single-task simulation.

Task	D	Min	Max	Mean	Median	SD
Risk-aversion task	-20	−1.850	−0.243	−1.046	−1.109	0.374
Risk-aversion task	-10	−4.091	−0.721	−1.396	−1.306	0.545
Risk-seeking task	10	0.396	9.558	5.219	5.320	2.492
Risk-seeking task	20	−0.978	7.338	3.118	2.860	2.199
