# Supplementary material for: Risk preference as an outcome of evolutionarily adaptive learning mechanisms: An evolutionary simulation under diverse risky environments
Source: PLoS One. 2024 Aug 1;19(8):e0307991. doi: 10.1371/journal.pone.0307991 (PMC11293680; doi:10.1371/journal.pone.0307991)
Supplement: S5 Table — (PDF) [file pone.0307991.s032.pdf]

S5 Table. Detailed value of Cohen's *d* and effect size in the single-task simulation.

Risk-aversion task (D = -20)

SD of Risky Op	Exp Value (risky vs safe)	Cohen's d	Effect
10	-50 vs -30	–1.270	large
10	-40 vs -20	–1.366	large
10	-30 vs -10	–1.134	large
10	-20 vs 0	–0.821	large
10	-10 vs 10	–0.292	small
10	0 vs 20	–0.371	small
10	10 vs 30	–1.850	large
15	-50 vs -30	–1.377	large
15	-40 vs -20	–1.274	large
15	-30 vs -10	–1.130	large
15	-20 vs 0	–1.004	large
15	-10 vs 10	–0.691	medium
15	0 vs 20	–0.444	small
15	10 vs 30	–1.357	large
20	-50 vs -30	–1.425	large
20	-40 vs -20	–1.292	large
20	-30 vs -10	–1.041	large
20	-20 vs 0	–1.109	large
20	-10 vs 10	–0.588	medium
20	0 vs 20	–0.243	small
20	10 vs 30	–0.981	large
25	-50 vs -30	–1.388	large
25	-40 vs -20	–1.470	large
25	-30 vs -10	–0.994	large
25	-20 vs 0	–1.021	large
25	-10 vs 10	–1.023	large
25	0 vs 20	–0.670	medium
25	10 vs 30	–0.943	large
30	-50 vs -30	–1.549	large
30	-40 vs -20	–1.387	large
30	-30 vs -10	–1.333	large
30	-20 vs 0	–1.126	large
30	-10 vs 10	–0.999	large
30	0 vs 20	–0.513	medium
30	10 vs 30	–1.131	large

Risk-seeking task (D = +20)

SD of Risky Op	Exp Value (risky vs safe)	Cohen's d	Effect
10	-10 vs -30	2.405	large
10	0 vs -20	3.006	large
10	10 vs -10	2.985	large
10	20 vs 0	1.550	large
10	30 vs 10	–0.978	large
10	40 vs 20	–0.677	medium
10	50 vs 30	–0.668	medium
15	-10 vs -30	5.895	large
15	0 vs -20	5.014	large
15	10 vs -10	3.721	large
15	20 vs 0	2.212	large
15	30 vs 10	0.856	large
15	40 vs 20	0.628	medium
15	50 vs 30	0.454	small
20	-10 vs -30	7.338	large
20	0 vs -20	5.691	large
20	10 vs -10	4.336	large
20	20 vs 0	2.424	large
20	30 vs 10	1.714	large
20	40 vs 20	1.490	large
20	50 vs 30	1.400	large
25	-10 vs -30	7.333	large
25	0 vs -20	5.534	large
25	10 vs -10	4.687	large
25	20 vs 0	2.975	large
25	30 vs 10	2.271	large
25	40 vs 20	2.088	large
25	50 vs 30	2.744	large
30	-10 vs -30	7.059	large
30	0 vs -20	5.975	large
30	10 vs -10	4.946	large
30	20 vs 0	3.151	large
30	30 vs 10	2.772	large
30	40 vs 20	2.860	large
30	50 vs 30	3.949	large

Risk-aversion task (D = -10)

SD of Risky Op	Exp Value (risky vs safe)	Cohen's d	Effect
10	-40 vs -30	−1.580	large
10	-30 vs -20	−1.848	large
10	-20 vs -10	−1.145	large
10	-10 vs 0	−1.066	large
10	0 vs 10	−0.866	large
10	10 vs 20	−1.500	large
10	20 vs 30	−4.091	large
15	-40 vs -30	−1.459	large
15	-30 vs -20	−1.553	large
15	-20 vs -10	−1.380	large
15	-10 vs 0	−1.095	large
15	0 vs 10	−1.223	large
15	10 vs 20	−0.983	large
15	20 vs 30	−1.956	large
20	-40 vs -30	−1.590	large
20	-30 vs -20	−1.453	large
20	-20 vs -10	−1.280	large
20	-10 vs 0	−1.245	large
20	0 vs 10	−1.102	large
20	10 vs 20	−0.920	large
20	20 vs 30	−1.687	large
25	-40 vs -30	−1.510	large
25	-30 vs -20	−1.570	large
25	-20 vs -10	−1.634	large
25	-10 vs 0	−1.069	large
25	0 vs 10	−1.306	large
25	10 vs 20	−0.721	medium
25	20 vs 30	−1.279	large
30	-40 vs -30	−1.491	large
30	-30 vs -20	−1.489	large
30	-20 vs -10	−1.552	large
30	-10 vs 0	−1.233	large
30	0 vs 10	−1.037	large
30	10 vs 20	−0.788	medium
30	20 vs 30	−1.141	large

Risk-seeking task (D = +10)

SD of Risky Op	Exp Value (risky vs safe)	Cohen's d	Effect
10	-20 vs -30	8.571	large
10	-10 vs -20	6.483	large
10	0 vs -10	5.404	large
10	10 vs 0	2.868	large
10	20 vs 10	0.636	medium
10	30 vs 20	0.532	medium
10	40 vs 30	0.396	small
15	-20 vs -30	8.930	large
15	-10 vs -20	8.422	large
15	0 vs -10	5.763	large
15	10 vs 0	3.318	large
15	20 vs 10	2.143	large
15	30 vs 20	2.973	large
15	40 vs 30	4.273	large
20	-20 vs -30	9.558	large
20	-10 vs -20	8.017	large
20	0 vs -10	5.954	large
20	10 vs 0	3.435	large
20	20 vs 10	3.161	large
20	30 vs 20	3.837	large
20	40 vs 30	4.802	large
25	-20 vs -30	9.192	large
25	-10 vs -20	7.710	large
25	0 vs -10	5.723	large
25	10 vs 0	3.869	large
25	20 vs 10	3.669	large
25	30 vs 20	4.271	large
25	40 vs 30	5.320	large
30	-20 vs -30	8.662	large
30	-10 vs -20	8.279	large
30	0 vs -10	6.737	large
30	10 vs 0	4.659	large
30	20 vs 10	4.052	large
30	30 vs 20	5.603	large
30	40 vs 30	5.456	large

Note. Effect indicates the magnitude of effect size based on the Cohen's criteria [2], by which the magnitude was classified into none (~0.2), small (0.2~0.5), medium (0.5~0.8), and large (0.8~).

Reference

2. Cohen J. Statistical power analysis for the behavioral sciences (2nd Ed.). Hillsdale, NJ: Lawrence Erlbaum Associates; 1988.
