# Supplementary material for: Risk preference as an outcome of evolutionarily adaptive learning mechanisms: An evolutionary simulation under diverse risky environments
Source: PLoS One. 2024 Aug 1;19(8):e0307991. doi: 10.1371/journal.pone.0307991 (PMC11293680; doi:10.1371/journal.pone.0307991)
Supplement: S6 Table — (PDF) [file pone.0307991.s033.pdf]

S6 Table. Summary of statistics of Cohen's *d* in the multiple-task simulation.

Multiple-Task Simulation

Simulation Condition	Min	Max	Mean	Median	SD
risk seeking/aversion = 0/4	–5.283	1.541	–1.235	–1.158	1.321
risk seeking/aversion = 1/3	–5.043	7.241	1.733	1.578	2.039
risk seeking/aversion = 2/2	–4.417	9.210	2.333	2.016	2.297
risk seeking/aversion = 3/1	–1.644	10.815	3.178	2.875	2.225
risk seeking/aversion = 4/0	–1.976	12.104	4.266	3.964	2.524
