# Supplementary material for: Risk preference as an outcome of evolutionarily adaptive learning mechanisms: An evolutionary simulation under diverse risky environments
Source: PLoS One. 2024 Aug 1;19(8):e0307991. doi: 10.1371/journal.pone.0307991 (PMC11293680; doi:10.1371/journal.pone.0307991)
Supplement: S7 Table — (PDF) [file pone.0307991.s034.pdf]

S7 Table. Frequency of effect size in the multiple-task simulation.

Multiple-Task Simulation

	Effect			
Simulation Condition	large	medium	small	none
risk seeking/aversion = 0/4	63	16	15	6
risk seeking/aversion = 1/3	84	11	3	2
risk seeking/aversion = 2/2	91	4	3	2
risk seeking/aversion = 3/1	94	4	0	2
risk seeking/aversion = 4/0	95	2	3	0

Note. The value is the frequency of an effect. Effect indicates the magnitude of effect size based on the Cohen’s criteria [2], by which the magnitude was classified into none (~0.2), small (0.2~0.5), medium (0.5~0.8), and large (0.8~).
