# Supplementary material for: Risk preference as an outcome of evolutionarily adaptive learning mechanisms: An evolutionary simulation under diverse risky environments
Source: PLoS One. 2024 Aug 1;19(8):e0307991. doi: 10.1371/journal.pone.0307991 (PMC11293680; doi:10.1371/journal.pone.0307991)
Supplement: S8 Table — (PDF) [file pone.0307991.s035.pdf]

S8 Table. Summary of statistics of SD ratio in the single-task simulation.

Task	D	Min	Max	Mean	Median	SD
Risk-aversion task	-20	1.081	3.553	2.059	1.699	0.810
Risk-aversion task	-10	1.281	3.642	2.213	1.853	0.816
Risk-seeking task	10	1.497	4.622	3.139	3.222	0.898
Risk-seeking task	20	0.540	4.166	2.540	2.664	1.073
