# Supplementary material for: Risk preference as an outcome of evolutionarily adaptive learning mechanisms: An evolutionary simulation under diverse risky environments
Source: PLoS One. 2024 Aug 1;19(8):e0307991. doi: 10.1371/journal.pone.0307991 (PMC11293680; doi:10.1371/journal.pone.0307991)
Supplement: S9 Table — (PDF) [file pone.0307991.s036.pdf]

S9 Table. Summary of estimated parameters of logistic regression analysis.

Risk-aversion task (D = -20, beta = 0.25)

Task	Intercept	alpha n	alpha p
N(-50,20) vs N(-30,5)	1.933	5.114	−0.518
N(-40,20) vs N(-20,5)	1.991	5.120	−0.557
N(-30,20) vs N(-10,5)	2.089	5.105	−0.627
N(-20,20) vs N(0,5)	2.280	4.927	−0.703
N(-10,20) vs N(10,5)	2.780	3.969	−0.677
N(0,20) vs N(20,5)	3.355	3.335	−0.885
N(10,20) vs N(30,5)	2.612	6.289	−1.571

Risk-seeking task (D = +20, beta = 0.25)

Task	Intercept	alpha n	alpha p
N(-10,20) vs N(-30,5)	−3.583	5.992	−0.975
N(0,20) vs N(-20,5)	−3.697	6.165	−0.990
N(10,20) vs N(-10,5)	−3.800	6.312	−1.002
N(20,20) vs N(0,5)	−3.888	6.444	−1.011
N(30,20) vs N(10,5)	−3.763	6.148	−0.843
N(40,20) vs N(20,5)	−2.270	3.474	0.067
N(50,20) vs N(30,5)	−1.035	1.674	0.509

Note. Dependent variable was the number of times of 500 trials that an agent chose the safe option. Independent variable was α_n and α_p. The logistic regression model was fitted to the data of Fig 3 for each task. The column "Intercept", "alpha n", and "alpha p" indicate the estimated value of intercept, partial regression coefficient of α_n and that of α_p, respectively. It should be noted that because the value of the independent variable ranged from 0.01 to 1.00 in the equal increments, it is possible to compare the estimated coefficient. The table shows that the coefficient of α_n was consistently larger than that of α_p, supporting that the α_n has larger impact on the choice than α_p.
