# Supplementary material for: Risk preference as an outcome of evolutionarily adaptive learning mechanisms: An evolutionary simulation under diverse risky environments
Source: PLoS One. 2024 Aug 1;19(8):e0307991. doi: 10.1371/journal.pone.0307991 (PMC11293680; doi:10.1371/journal.pone.0307991)
Supplement: Attachment — Submitted filename: ResponseLetter_Homma.docx [file pone.0307991.s037.docx]

PONE-D-23-31441

Title: Risk preference as an outcome of evolutionarily adaptive learning mechanisms: an evolutionary simulation under diverse risky environments

Authors: Shogo Homma and Masanori Takezawa

Dear Prof. Akaishi,

We would like to thank the reviewers and the editor for their comprehensive and valuable reviews. We have addressed all the editorial and referee’s comments and believe the revision made to the manuscript greatly improved its clarity and richness. Throughout the response letter, we have indicated the line numbers of the revised manuscript with history to refer to which part we have revised. The revised parts are colored in red in the manuscript with history.

While revising the manuscript, we identified three typos in the previous manuscript and made the following corrections. We apologize for these changes.

1. Line 414: "the relationship $\alpha_{n}$ > $\alpha_{p}$ evolved" → "the relationship $\alpha_{p}$ > $\alpha_{n}$evolved"
2. Line 463: "This study proposes that " → "Our study proposes that".
   The sentence indicates the novel contribution of our study by contrasting the previous study. A word "This study" wrongly indicates the previous study referred in the previous sentence.
3. Line 564: Equation (3) "$V_{t}\left( a \right)=V_{t}(a)$" → "$V_{t+1}\left( a \right)=V_{t}(a)$"
   $V_{t}\left( a \right)$ is updated to that of next trial: $V_{t+1}\left( a \right)$.

COMMENTS BY EDITOR

When submitting your revision, we need you to address these additional requirements.
1. Please ensure that your manuscript meets PLOS ONE's style requirements, including those for file naming. The PLOS ONE style templates can be found at https://journals.plos.org/plosone/s/file?id=wjVg/PLOSOne_formatting_sample_main_body.pdf and https://journals.plos.org/plosone/s/file?id=ba62/PLOSOne_formatting_sample_title_authors_affiliations.pdf

We revised the title page, main body, and figures to meet the PLOS ONE style. We have listed how we revised them point by point below.

- We revised the affiliations to include Department, Institution, City, and Country
- We enlarged the font of Heading titles and made them bold.
- We made the caption title bold and set a figure title of Fig 4
- We made figure citation proper (e.g., Fig. 1a -> Fig 1a)
- We displayed the equation number
- We uploaded figures of the main text separately in TIFF format

2. We are unable to open your Supporting Information file [__MACOSX]. Please kindly revise as necessary and re-upload.

We are sorry for having uploaded unreadable file. We revised and uploaded the new Supporting Information file.

COMMENTS BY REVIEWER 1

The authors report the results from a series of agent-based model simulations where they studied the evolution of reinforcement learning parameters under multiple risky two-armed bandit tasks. It had been well-known that a reinforcement learning often shows risk aversion in some ranges of parameter combinations even if there is a positive risk premium in choosing risky options. Such a potentially suboptimal risk-aversion would not always emerge, and reinforcement learning could evolve to be risk-seeking if natural selection pressures act on a longer-term payoff obtained a single risky bandit task. However, if agents' fitnesses were determined by multiple different bandit tasks, it was unclear how reinforcement learning would evolve and what type of risk-taking biases would emerge through natural selection.

To address this, the authors assumed that agents had to perform four different bandit tasks whose risk profile was systematically varied. The authors found that agents evolved a sort of flexible payoff-maximising bias, that is, showing risk aversion when the risk premium was negative (i.e. the risk aversion tasks), while resulting in risk seeking when the risk premium was positive (i.e. the risk seeking tasks). For someone who has been studying reinforcement learning and risk taking behaviour, this result itself did not seem to be so surprising and it was just consistent with what we knew.

What was interesting though was that there were two learning rate parameters assumed, one was for positive RPEs and another for negative RPEs, which were evolving separately. It was also very nice that the authors had thoroughly compared the main results with both a simpler version of RL with a single learning rate and a more complex candidate model with a choice trace parameter (called a hybrid model). Then the authors found that it was mostly a negative learning rate that shaped agents' risk taking biases, which makes totally sense because avoiding underestimation of risky option is key to prevent the hot stove effect.

Overall, although the findings were not so surprising to someone in the decision science, the evolution of learning behaviour and how it relates to domain general risk taking biases would be of interest of wider audience including behavioural and evolutionary ecologists. The study is technically very sound and the authors present it in a concise way. However, before accepting this manuscript for publication, there are several key points that I believe the authors should address in a revision.

We really appreciate your valuable feedback and suggestions for improving our paper. We have addressed each comment below, and we consider that your comments significantly improved the quality of our paper.

Major points: (1) Relate more to the previous works on the hot stove effect

The authors should not overstate the novelty of their results, and should pay fair credits to previous works. For instance, the current manuscript reads that "Surprisingly, the evolved agents were successful in choosing the more rewarding option in both the risk-averse and risk-seeking tasks"; however, it was not surprising at all, I am afraid. The simple RL model considered here cannot become risk-seeking in any range of parameter combinations when the risk premium is negative (See for example Eq. (2) in Denrell (2007)). In other words, RLs always learn to become risk aversive irrespective of the task profile unless the risk premium was large enough. This implies in principle that RL agents who have successfully evolved to be risk seeking under the risk-seeking tasks, still should always be risk aversive under the risk-aversive tasks regardless of whatever the learning rates and the inverse temperature they evolved. In general, I would appreciate if the authors can point out how their findings can relate to the well-known hot stove effect, highlighting what is truly news in the literature and what is a replication of the previous literature (which is also nice things to see).

Thank you very much for your suggestions. First, we want to point out that Eq. (2) in Denrell (2007) assumes a risk-averse (safe) option has zero variance and cannot be directly applied to our setting, which assumes non-zero variance for a safe option. Second, Denrell (2007) investigated the functional role of the single learning rate model, while we investigated the two learning rate model. Despite these differences, we agree with your suggestions for the necessity of discussing our research by relating to Denrell (2007). Hence, we revised the Discussion (Lines 420-425, and Lines 427-434) – we now mentioned Denrell (2007) and discussed what is new in our study.

(2) The connection to the gain/loss domain was misleading

The authors investigated the behaviour of evolved agents by varying the positions of agents' initial q-values compared to the tasks' average reward value. When agents started the task with an optimistic prior belief, they tended to be more explorative at the beginning, which made it easier to overcome the hot stove effect when the risk premium was positive. On the other hand, when they had an pessimistic prior, they were prone to get stuck in the safer option. This result makes totally a sense, but has nothing to do with the gain/loss domain, let alone with the prospect theory that was about the decisions from description but not from experience. RL agents do not care about the "domain" of the payoff because what they learn is a difference between their current q-value and payoff just obtained (that is RPEs). They could have integrated the PT by assuming that learning were based on agents' subjective utilities rather than payoffs, that is, utility = f(payoff). But I guess that that direction of modelling would not be what they aimed to do here.

In prospect theory, the gain/loss is defined relative to the status quo, which varies depending on the context, and the identical outcome could be perceived either as gain or loss. In behavioral economics, relative and absolute gain/loss are strictly distinguished. When people change risk preferences in response to absolute signs of outcomes, it is called the *reflection effect* (Tversky & Kahneman, 1981). When people change their risk preferences in response to relative gain/loss, it is called the *framing effect*. Our manipulation of initial *Q* values could be interpreted as manipulating the initial status quo, and thus, our manipulation of the gain/loss tasks is comparable to the definition of the gain/loss domains in prospect theory. Our study showed that the direction of the observed behavioral pattern (Fig 4 in the main text) is consistent with the *framing effect* in prospect theory.

Here, we are not arguing that the prospect theory explains our findings or that our model can replace it. We are also not arguing that reinforcement learners subjectively care about the gain/loss domains defined above. We are simply pointing out the resemblance between the behavioral patterns observed in our study and those described in the prospect theory. It is surprising to us that similar behavioral patterns emerge in different contexts, and it has significant academic value to point out the resemblance.

Having said that, the point that the evolved agents' behaviour was consistent with what PT predicted was interesting. This might imply that human risk taking behaviour in the gain and loss domains may not always be a result of the biased subjective utility and probability as PT claimed. Instead, the same behavioural pattern could have been generated by simple RL mechanisms that do not care about whether it is the gain or loss domain.

So in sum, I disagree with what the authors discussed in lines 432 - 436. What we observed in this manuscript has no direct relations to PT. Instead, in a revision, the authors could discuss what it would mean that two different mechanisms (that is, PT and RL) happen to show a similar pattern. If the authors still believe that what was observed in their results could be relevant to PT, more detailed discussion would be needed. I think this might also be related to the description and experience gap:

Hertwig, R., & Erev, I. (2009). The description–experience gap in risky choice. Trends in cognitive sciences, 13(12), 517-523.

Prospect theory is known to be a descriptive theory because the famous s-shaped utility functions are obtained by fitting a function to the behavioral choice data. In other words, prospect theory the mere description but not explanation for the observed choice patterns. In contrast, priority heuristic (Brandstätter, Gigerenzer, and Hertwig, 2006) and decision sampling theory (Stewart, Chater, and Brown, 2006) are examples of an attempt to identify mechanisms producing choice patterns described by prospect theory. Hence, what should be contrasted with reinforcement learning, a mechanism producing choice behaviors in decision-by-experience settings, is not prospect theory but such process models.

Our wordings in the previous manuscript were insufficient to clarify this point. We changed all the wording such as “(behavioral patterns) predicted by prospect theory) to “described by prospect theory” across the manuscript. Although we already mentioned the the description-experience gap in the Discussion section (Lines 466-481 of the main text), we modifed the entire paragraph for emphasizing how our study is related to prospect theory (Lines 458-461 and Lines 466-481).

We really appreciate your feedback to clarify the implications in relation to prospect theory.

(3) What about the evolved variations in alphas?

The evolved variation in alpha_p was larger than that of alpha_n. It would be nice if the authors can explain or discuss what this pattern might imply.

Thank you for the comment about the difference in variance between the two learning rates. Because we reported the variance of $\alpha_{p}$ in the single-task simulations and multiple-task simulations, we have addressed both simulations. As for the variance in the single-task simulations (Fig 1b), we already discussed the implication of smaller variance in $\alpha_{n}$ in the Results section (Lines 212-213). We added a sentence in the Discussion section of the main text (Lines 483-485) to emphasize that a smaller variance of $\alpha_{n}$ suggests a stronger selection pressure on the parameter. As for the variation in the multiple-task simulations (Fig 2a), we observed a larger variability in the evolved $\alpha_{p}$ was correlated with a task property of a simulation: the location of two options (or normal distributions) (Fig 2b). This means that the evolution of $\alpha_{p}$ was not the mere product of the drift but was caused by adaptation to a task property. We revised the Results Section of the main text (Lines 258-261) to clarify what the correlation between the evolved $\alpha_{p}$ and the locations of the two options means. We believe that the revision improved the clarity of our results.

Minor comments:(4) Lines 110 - 112: As for the evolution of the Rescorla-Wagner type of learning rate, the authors could cite this:

P.C. Trimmer, J.M. McNamara, A.I. Houston, J.A.R. Marshall. Does natural selection favour the Rescorla-Wagner rule? Journal of Theoretical Biology, 302 (2012), pp. 39-52, 10.1016/j.jtbi.2012.02.014

Thank you for recommending a paper about the evolution of the learning rate of the RW model. Following your suggestion, we added the reference to the paper (Line 103 in the main text). Thank you again for the suggestion to enrich our introduction.

(5) Also, though they are not evolutionary studies, the following studies have investigated functional aspects of reinforcement learning mechanisms under different kinds of risks and social environments. In my view, one of key factors of the current study was that they focused on multiple tasks simultaneously. The citations below were analysing the function of RL under only a single environment. So I guess citing them could make it easier to highlight the uniqueness of the authors' current study using multiple bandits.

Denrell, J. (2005). Why most people disapprove of me: experience sampling in impression formation. Psychological review, 112(4), 951.

Toyokawa, W., & Gaissmaier, W. (2022). Conformist social learning leads to self-organised prevention against adverse bias in risky decision making. eLife, 11, e75308.

Following your suggestions, we highlighted our contribution focusing on multiple environments in the Discussion section and cited the two papers as previous studies focused on the function of reinforcement learning in a single risky or social environment (Lines 425-427 in the main text). Thank you very much for your constructive suggestions.

(6) On Fig. 2(C): To back up their argument that having at least one risk-seeking environment would promote the evolution of the flexible risk taking (that is, risk seeking only under the positive risk premium), they should also check how agents who had evolved under the risk aversion only condition (that is, the "0/4" condition) would behave in a risk seeking task. For example, the authors could show such data on Fig. 2C too (for instance, running a new risk-seeking task with "0/4" agents and show their risk aversion bias at the 3000th generation of the "0/4" panel). They might not be able to show risk seeking if their evolved environment was "0/4". The same could be done for the "4/0" condition too.

Thank you very much for raising the important issues. Following your suggestion, we added new data and updated Fig 2c in the main text (please see the old and new Figures below for comparison). We simulated how the agents in the first and last generations of 0/4 condition would behave in a randomly sampled risk-seeking task. Similarly, we simulated the behaviors of agents of 4/0 condition in a risk-aversion task. As you see in the new version of Figure 2c, the performance of the evolved agents of 0/4 condition gets worse in the risk-seeking tasks (i.e., more risk-averse). The decrease in performance was also observed in the agents of 4/0 condition in the risk-aversion tasks, too (i.e., more risk-seeking). These results show that agents need to experience both types of tasks in order to flexibly choose the more rewarding option. We revised the result (Lines 284-287 and Lines 291-294) and added the conclusion in the main manuscript (Lines 295-296) and detail of the simulation setting in the Figure caption (Lines 318-321). Your comment significantly enriched the arguments of our paper. We are grateful for the constructive suggestion.

**Old version of Fig 2c**

**New version of Fig 2c**

Red circles indicate the added data. The circles are not displayed in the manuscript.

COMMENTS BY REVIEWR 2

The study “Risk preference as an outcome of evolutionarily adaptive learning mechanisms: an evolutionary simulation under diverse risky environments” presents the evolution of positive and negative learning rates in various risky contexts. The authors conducted a series of simulations with reinforcement learning models and found that, over generations, the negative learning rate became higher than the positive learning rate when a safe option (i.e., with lower variance) was associated with a higher mean outcome. Conversely, the negative learning rate became smaller than the positive learning rate when the risky option (i.e., with higher variance) was associated with a higher mean outcome. These patterns were consistent not only within single risky contexts but also in environments with multiple risky contexts. The authors also established an association between learning rates and risk attitude through simulations with different combinations of learning parameter values. Furthermore, the evolved learning rates were applied to tasks where the mean of outcome distributions was controlled for risky and non-risky options. This revealed a heightened tendency for risk aversion in the gain domain compared to the loss domain, particularly when the evolved learning rates originated from tasks featuring at least one risk-seeking context. Overall, the authors conducted a systematic investigation into the evolution of learning rates and their relationship with risk attitudes. However, I have three major points (and a few minor ones) that could enhance the clarity of the conclusions.

First of all, thank you very much for your thorough review and valuable suggestions to clarify the results of our paper.

Major points: 1. Conceptual:

It remains unclear if risk preference is related to learning bias (e.g., the difference between positive and negative learning rate) or learning rate (either positive or negative) itself. If the authors are interested in the learning bias (as mentioned in the introduction and conclusion), then I would expect more statistical analysis on the difference between positive and negative learning rates (see also the next two points). For example, the correlation between learning bias and level of risk aversion. So far, the results and interpretation mainly focus on the role of each learning rate (e.g., how does each learning rate evolve; “a negative learning rate had more control over adaptive risky behavior than a positive learning rate” (Line 189)), which makes me wonder how much evidence supports the conclusion “the complicated aspect of risk preference can be captured by learning bias in reinforcement learning as an innate constraint shaped by an evolutionary process.” (Line 502-504)

We really appreciate the comment about the quite important issue we didn’t fully discuss in the previous manuscript. We used the term "learning bias" as "an innate constraint to influence the direction of learning", so considered both evolved $\alpha_{p}$ and $\alpha_{n}$ to be learning bias. We revised the main text (Lines 69-70) to clarify the definition. However, as you point out, the difference between $\alpha_{p}$ and $\alpha_{n}$ (i.e., ($\alpha_{n}$ - $\alpha_{p}$)/($\alpha_{n}$ + $\alpha_{p}$), hereafter called “Niv index” as the equation was introduced by Niv et al., (2012) ) can be considered as an index of learning bias. As we will describe the details of the results here and in the next comment, the two learning rates independently influenced the behaviors more than the Niv index. Therefore, we mainly reported the results of two learning rates. However, because we did not show the relationship between the Niv index and the behaviors, we have decided to add new figures in Supporting Information and modified the main text.

First, we created the histogram of the Niv index for the single-task simulation from the data of Fig 1b. The distribution of the Niv index in the last generation was skewed in the positive direction in risk-aversion tasks and the negative direction in the risk-seeking tasks. Note some exceptions were seen in risk-seeking tasks, as already mentioned in the main text (Lines 201-202). The corresponding risk aversion of the evolved agents in each task was already shown in Fig 1a in the main text. We added the figure in the Supporting Information as S17 Fig and the descriptions in the main text (Lines 213-217).

Next, we created the histogram of the Niv index for the multiple-task simulation from the data of Fig 2a (Only the first and last generations were extracted). The distribution of the Niv index in the last generation was skewed in the negative direction except 0/4 condition. The corresponding risk aversion of the evolved agents in each condition was already shown in Fig 2c in the main text. We added the figure in the Supporting Information as S18 Fig and the mention in the main text (Lines 274-277).

Finally, we created the scatter plots between the Niv index and risk tendency from the data in Fig 3. A positive correlation between them was observed. However, there was a large variance in the risky behavior even when the Niv index values were identical. This suggests that it is better to focus on the two learning rates rather than their difference to predict the behavior. We added the figure in Supporting Information (S19 Fig) and modified the main text (Lines 341-346). In order to avoid confused about the definition of learning bias, we revised the sentence (Lines 122-123) to avoid confusion.

2. Analysis:

One of the authors’ summaries is that “ is consistently influenced by natural selection unlike , and the value of influenced the agents’ behavior more strongly than ”. If this is the case, I have three follow-up questions:

a. Why did the authors focus on a positive learning rate rather than a negative learning rate (or learning bias: – ) in Figure 2b?

Thank you for the important question. The reason why we focused on the $\alpha_{p}$ in Fig 2b was the following. Results of the multiple-task simulations showed that the variance of evolved $\alpha_{p}$ was much larger than the variance of $\alpha_{n}$. Large variances in evolved populations are signals of no/weak selection pressure on the trait (here, $\alpha_{p}$). However, Fig 2b shows it is not the case: the evolution of $\alpha_{p}$ was influenced by the exogenous task property. In short, we used Fig 2b to illustrate there was a selection pressure on of $\alpha_{p}$. We revised the main text Lines 258-261 to clarify the implications of Fig 2b. Thank you again for your question to improve the clarity.

b. Although the choice pattern is dominated by the negative learning rate (αn), the variance of choice is similar to the variability of positive learning rate (αp) especially in the risk-seeking task in Figure1. Therefore, I wonder, is it possible that behavior is influenced by two learning rates. For example, the index of risk aversion is related to the difference between p and (Niv et al., 2012). The result will provide additional (and more straightforward) insight into “how evolved learning rates regulate behavioral patterns” (Line 174).

The conclusion that $\alpha_{n}$ had a stronger influence than $\alpha_{p}$ in a risky environment was derived from Fig 3. Please see the scatter plots of the relationship between the Niv index and risk aversion rate, which is calculated from the data of Fig 3 (Supporting Information, S19 Fig). By definition, the Niv index and the choice should be correlated, and it was confirmed in our study. However, there was a large variance in risk aversion rate even when the value of the Niv index was identical. Therefore, we analyzed the behaviors by focusing on the two learning rated rather than the Niv index. As we already mentioned in the previous response, we added the figure in Supporting Information (S19 Fig), and added the explanations in the main text (Lines 341-346).

3. More statistical results are required to support the arguments.

According to the visual inspection in Figure 1 and 3, the arguments “a negative learning rate had more control over adaptive risky behavior than a positive learning rate” and “the evolved showed larger variability depending on the simulation” are true for the risk-seeking task only. With the same figures, however, it is unclear whether the same arguments also apply to the risk-aversion task. Specifically, in the risk-aversion task

We really appreciate the valuable suggestion to support our results in a more elaborate and quantitative way. You discussed that two conclusions can be true only in risk-seeking tasks of the single-task simulation. However, we derived the second conclusion from the multiple-task simulation, so we didn’t discuss it here. The first conclusion was already mentioned in the Introduction section. However, the sentence was vague about the result it was referring to. Thus, we modified the sentence in Line 179 to clarify Fig 3 is mentioned here.

We can derive the conclusion from Fig 3 because a slight difference of $\alpha_{n}$ greatly changes the risky behavior compared to that of $\alpha_{p}$ (which is already mentioned in Lines 337-339 in the main text). However, the conclusion was not quantitatively tested. So, we conducted the additional analysis. Please see the next section about the details.

a. whether the difference between αn and αp is significant? If yes, then the current conclusion holds. Otherwise, the conclusion might be restricted to the risk-seeking task.

Although the first sentence was related to Fig 3, your suggestion about the additional analysis of Fig 1 is really helpful in supporting our conclusions. We conducted an additional analysis of Fig 1 by comparing the mean and variance of $\alpha_{p}$ and $\alpha_{n}$. Because computer simulations can easily increase the sample size, it is not informative to simply conduct null hypothesis testing by checking *p* values. Instead, we calculated the effect size, Cohen's *d*, between the evolved $\alpha_{p}$ and $\alpha_{n}$. We found that the values of effect size were sufficiently large in most tasks reported in Fig 1 (see S20 Fig and S4 and S5 Tables in Supporting Information). We mentioned them in the main text (Lines 202-204).

In addition, we conducted the same analysis for the multiple-task simulations. We created the histogram of Cohen's *d* for each condition. We found that Cohen’s *d* gets a large positive value when agents experience at least one risk-seeking task. We added the figure and tables in Supporting Information (S21 Fig and S6 and S7 Tables) and mentioned them in the main text (Lines 267-268).

b. whether the variability of αp is significantly larger than the variability of αn?

We also analyzed the variance difference between the evolved $\alpha_{p}$ and $\alpha_{n}$. We calculated the ratio of two variances for each task and checked which one was larger than the other. As a result, we confirmed that the variance of $\alpha_{p}$ is larger than that of $\alpha_{n}$in most tasks in Fig 1. We added new figures and tables in Supporting Information (S22 Fig and S8 Table) and mentioned them in the main text (Lines 211-212).

c. whether the impact of αn is larger than the impact of αp on the risk attitude? Again, this is not clear from the Figure 3.

Using the data in Fig 3, we conducted the logistic regression analysis for the frequency of risk aversion with $\alpha_{p}$ and $\alpha_{n}$ as predictor variables. Because we set the data range from 0.01 to 1.00 in equal increments in Fig 3, it is possible to compare the estimated value of the partial regression coefficient of two learning rates and see which has a larger impact on the risk-averse behavior. We found that the estimated coefficients of $\alpha_{n}$ were consistently larger than that of $\alpha_{p}$. This supports our conclusion that $\alpha_{n}$ has a larger impact on the learning of risky behavior. We added the tables of the estimated parameters of the regression analysis in the Supporting Information (S9 Table) and mentioned them in the main text (Lines 339-340). Thank you again for the constructive suggestion to support our conclusions in a more quantitative way, which surely improved our manuscript.

Minor points: 1. Could the authors elaborate on the relationship between risk sensitivity and probability matching more in the introduction section? It is difficult for me to understand why risk sensitivity could be used to explain probability matching.

Thank you for the question. We introduced probability matching as an example of the irrational behavioral pattern that the evolution of the learning algorithms paradigm could explain. Although it is unrelated to the problem of risk sensitivity, our discussion in the original manuscript was not clear enough in this regard. We added a sentence in the Introduction section (Lines 53-55).

2. Line 410-418. Please elucidate the unique role of the two learning rates when compared to a single learning rate model in shaping risk attitudes. Specifically, if a single learning rate model can generate equivalent choice patterns to what the two-learning-rate model predicts, it is essential to clarify the added value or unique capabilities of the learning bias that the single learning rate model cannot provide. Importantly, the explanation is helpful for readers to understand the relationship between risk preference and learning bias (which is not available in the single-learning-rate model).

Thank you for raising the important point. We already discussed the functional role of two learning rates in reinforcement learning in the Discussion section (Lines 435-445). We introduced the two learning rates because it is a more biologically plausible implementation as a value-computation system in the brain. However, it is also true that we did not find a unique role of the two learning rates at the functional/behavioral level in our simulations. We consider that this has two implications. First, our tasks might be too simple, so the two reinforcement learning models did not exhibit a difference in behavioral performance. Second, the evolution of two learning rates is affected by factors other than behavioral level: for example, the phylogenetic or physiological constraints. We modified the main text (Lines 442-445) to clarify our considerations on this point.

3. Figure3 suggests that risk attitudes are influenced by context, even when learning components, such as the positive learning rate (p) and positive learning rate (n), are 0 or close to 0. To gain a deeper insight into the significance of the learning mechanisms in shaping risk attitudes, I would like to know why the differences in risk attitudes between risk-aversion and risk-seeking tasks persist despite values are not (or slowly) updated.

In the simulations, we didn’t consider zero learning rate values because, as is evident from the value updating rule in Eq (1), *Q* values do not change at all in this case. On the other hand, a learning rate close to zero (e.g., the minimum value in Fig 3 was set to 0.01) has significant influences on the behaviors because even when learning is slow, *Q* values are gradually updated and influence the behavior as is shown in Fig 3.

4. Line 79-82. Please clarify the definition of optimal options in the sentence. If the optimal option refers to the option associated with a higher anticipated outcome (i.e., the non-risky option in the risk-aversion task and the risky option in the risk-seeking task), then the evolution of fitness should be observed. Is that the case?

You are right. In our simulations, the optimal option is defined based on expected value.

On the other hand, I might misunderstand how the authors computed the probability of producing offspring. Could the authors clarify why the baseline fitness is 20 rather than the mean of the payoff across agents? I am wondering if the probability can be larger than 1 if the mean of the payoff is larger than 20 (e.g., in the context of 30 vs 50 or -30 vs -50).

We introduced the baseline fitness because the fitness must be positive values or the selection algorithm cannot calculate a probability of reproducing offsprings. Furthermore, it is a common basic assumption of evolutionary game theory, evolutionary biology and other related evolutionary/biological disciplines to assume baseline fitnes for keeping a weak selection pressure. Please see *p*.40 of McElreath & Boyd (2007) for the detailed background.

Reference: McElreath, R & Boyd, R. (2007) *Mathematical Models of Social Evolution: A Guide for the Perplexed*. University of Chicago Press.

5. The current study establishes a connection between learning rates and risk preferences, particularly emphasizing that individuals tend to exhibit greater risk aversion when n > p, aligning with prior findings (Niv et al., 2012). However, from an evolutionary standpoint, it raises the question of how the authors interpret earlier research that suggests humans have a tendency toward positivity bias (i.e., p > n) and an inclination towards risk aversion.

Thank you for raising the important point that needs to be clarified. As you discussed, the evolution of positivity bias ($\alpha_{p}$ > $\alpha_{n}$), which is expected to generate a risk-seeking behavior (Niv et al., 2012), looks inconsistent with the common notion that humans have risk-averse tendency. However, these seemingly contradicting results were obtained in different settings. The positivity bias in our simulation was observed as the result of evolutionary adaptation to the diverse environments where *the expected values of two options were NOT identical*. Previous research that reported risk aversion used tasks where *the expected value of two options was identical*. We tested the evolved agents with the tasks where the expected value of two options was identical. We then found the evolved population of agents exhibited the risk-averse tendency (or pattern described by prospect theory) like human participants in the experiments.

We revised the manuscript to clarify this point (the main text Lines 515-519). We are grateful for your valuable comment.

6. Learning strategy can be context-dependent. Therefore, it would be great if the authors could provide further insights into the extent to which the findings from the current study can be extended to alternative scenarios. For instance, can these results be applied to situations where agents have access to both chosen and unchosen outcomes, or when agents begin making assumptions about the foregone outcome (Ben-Artzi et al., 2023; Ting et al., 2022; Bavard et al., 2021).

We agree that the accessibility to information on foregone payoff is an important issue, which we didn’t discuss in the previous manuscript. We added the following considerations in Discussion section (Lines 532-542).

For instance, Denrell (2007) explicitly discussed that the learning of risky behavior is prevented when agents can access the information about forgone payoff. In our simulation, evolved small $\alpha_{n}$ values caused learning of risky options in risk-seeking tasks (We’ve already discussed this in the main text Lines 482-498). If agents could access the forgone payoff information, the selection pressure on $\alpha_{n}$ would decrease. However, it is natural to assume that agents experience both types of situations where foregone payoff information is accessible and not accessible. Applying the logic of multi task situations in our study, it is expected that the evolved agents are tuned to the mixture of both situations.

As you suggested (“when agents begin making assumptions about the foregone outcome (Ben-Artzi et al., 2023; Ting et al., 2022; Bavard et al., 2021).”), humans have a cognitive capacity for counterfactual thinking and can infer about foregone payoffs even when it is not accessible. It is quite an intriguing question to ask about the evolution of counterfactual capacity in the population of reinforcement learners. However, we worry that such a question exceeds the scope and capacity of our study. We are thankful for your deep and thorough thoughts about potential avenues for extending our research.

7.Figures:

a. The font size in each figure could be enlarged.

We enlarged the character font of all figures in the main text.

b. What does each dot represent in Figure 2b?

Each dot in Fig 2b represents the mean value of a simulation. We conducted 100 simulations for each condition, so each panel of Fig 2b has 100 dots. Your comment made us notice that the caption of Fig 2b was insufficient on this point. We modified the figure caption (Lines 313-315), which explains what the dots, blue lines, and shade area of Fig 2b represent.

c. The description “… agents learned a risk-averse behavior in almost all parameter regions except for where was close to zero.” in Line 326 is not visible in Figure 3.

We created new figures from Fig 3 by dividing the $\alpha_{n}$ axis by 0.1 and magnifying the heatmap. We added the additional figures in the Supporting Information (S23 Fig) and mentioned it in the main text (Line 333). The figures clearly show that the risk-neutral or even risk-seeking choices were seen in the area where the $\alpha_{n}$ values are close to zero, but not seen in the other area.

8.Suggestions for wording:

a. Figure: rate of risk averse -> probability of choosing non-risky option or p(choosing safe option)

b. Figure: risk of the risky option -> variance of the risky option

c. Line 343: agents successfully chose the risk-averse behavior -> agents successfully chose the risky option

Thank you for your suggestions. We modified the wording in the following manner. Firstly, we replaced "non-risky option" with "safe option" across the main text, Supporting Information, and figures to make the meaning clearer (by contrasting risky option vs safe option). As for figures, we updated Fig 1 in the main text and Supporting Information (S1-S5 Figs, S10 Fig, and S1 Table). Secondly, we replaced the "risk of the risky option" to "variance of the risky option" (Fig 1 in the main text, and S1, S3, and S5 Fig in Supporting Information). To ensure that the numeric value of risk (10, 15, 20, ...) represents standard deviation, we added the caption of “Each panel shows the different standard deviation of the risky option ($\sigma_{1}$ of normal distribution).” (Lines 230-231). Accordingly, we updated the figures and the caption of Supporting Information (S1, S3, and S5 Figs). Thirdly, we revised a sentence "agents successfully chose the risk-averse behavior" to "agents successfully chose the safe option" (the main text Line 354).

Although we understand that "probability" or "proportion" are better wordings, please allow us to keep using "Rate of Risk Aversion" because we frequently use it in our main text, supporting information, and figures.
