# Supplementary material for: Risk preference as an outcome of evolutionarily adaptive learning mechanisms: An evolutionary simulation under diverse risky environments
Source: PLoS One. 2024 Aug 1;19(8):e0307991. doi: 10.1371/journal.pone.0307991 (PMC11293680; doi:10.1371/journal.pone.0307991)
Supplement: Attachment — Submitted filename: Review_PLOSONE_revision.docx [file pone.0307991.s038.docx]

The revision successfully clarifies the primary concern regarding the of positive and negative learning rates in shaping risk attitudes. I have a few suggestions for the authors to further minimize potential confusion around some terms used in the paper. Notably, even without implementing these suggestions, I believe this work could be published in **PLOS ONE**.

1. It would be beneficial for the authors to add a sentence in the Results section emphasizing that the "safe" option is not entirely free of risk. This clarification would help readers understand that the safe option still involves a certain level of uncertainty (albeit lower than the risky option). Specifically, when discussing the Results section, adding this context will make it easier to interpret comparisons such as N(30, 10) vs. N(10, 5) (i.e., Line 201).

2. The authors have included a few sentences in the Discussion section to highlight the similarities and differences between their work and previous studies. I would encourage the authors to also emphasize the similarities and differences in the simulation settings between their study and prior research. For instance, they could cite papers that used the same definition of risky alternatives and the applied changes in the variance of risky options.
